# Leitlinie S1 für das Management postviraler Zustände am Beispiel Post-COVID-19

**DOI:** 10.1007/s00508-023-02242-z

**Published:** 2023-08-09

**Authors:** Susanne Rabady, Kathryn Hoffmann, Martin Aigner, Johann Altenberger, Markus Brose, Ursula Costa, Doris-Maria Denk-Linnert, Samuel Gruber, Florian Götzinger, Raimund Helbok, Katharina Hüfner, Rembert Koczulla, Katharina Kurz, Bernd Lamprecht, Stefan Leis, Judith Löffler, Christian A. Müller, Hans Rittmannsberger, Paulus S. Rommer, Paul Sator, Volker Strenger, Walter Struhal, Eva Untersmayr, Karin Vonbank, Johannes Wancata, Thomas Weber, Maria Wendler, Ralf-Harun Zwick

**Affiliations:** 1 Department Allgemeine Gesundheitsstudien, Kompetenzzentrum für Allgemein- und Familienmedizin, Karl Landsteiner Privatuniversität für Gesundheitswissenschaften, Dr. Karl-Dorrek-Str. 30, 3500 Krems, Österreich; 2 Leiterin der Abteilung Primary Care Medicine, Medizinische Universität Wien, Währinger Gürtel 18–20, 1090 Wien, Österreich; 3 Abteilung für Psychiatrie und psychotherapeutische Medizin, Karl Landsteiner Privatuniversität für Gesundheitswissenschaften, Dr. Karl-Dorrek-Str. 30, 3500 Krems, Österreich; 4 Pensionsversicherungsanstalt, Rehabilitationszentrum Großgmain, Salzburger Str. 520, 5084 Großgmain, Österreich; 5 Department Allgemeine Gesundheitsstudien, Kompetenzzentrum für Allgemein- und Familienmedizin, Karl Landsteiner Privatuniversität für Gesundheitswissenschaften, Dr. Karl-Dorrek-Str. 30, 3500 Krems, Österreich; 6 Ergotherapie und Handlungswissenschaft, fhg – Zentrum für Gesundheitsberufe Tirol GmbH/fh, Innrain 98, 6020 Innsbruck, Österreich; 7 Klinische Abteilung für Allgemeine Hals‑, Nasen- und Ohrenkrankheiten, Klin. Abteilung Phoniatrie-Logopädie, Medizinische Universität Wien, Währinger Gürtel 18–20, 1090 Wien, Österreich; 8 Department Allgemeine Gesundheitsstudien, Kompetenzzentrum für Allgemein- und Familienmedizin, Karl Landsteiner Privatuniversität für Gesundheitswissenschaften, Dr. Karl-Dorrek-Str. 30, 3500 Krems, Österreich; 9 Abteilung für Kinderheilkunde, Klinik Ottakring, Montleartstr. 37, 1160 Wien, Österreich; 10 Universitätsklinik für Neurologie, Johannes Kepler Universität Linz, Standort Neuromed Campus & Med Campus Kepler Universitätsklinikum GmbH, 4020 Linz, Österreich; 11 Dep. für Psychiatrie, Psychotherapie, Psychosomatik und Medizinische Psychologie, Universitätsklinik für Psychiatrie II, Medizinische Universität Innsbruck, Anichstr. 35, 6020 Innsbruck, Österreich; 12 Fachbereich Medizin, Klinik für Pneumologie Marburg, Baldingerstr., 35035 Marburg, Deutschland; 13 Innere Medizin II, Medizinische Universität Innsbruck, Anichstr. 35, 6020 Innsbruck, Österreich; 14 Universitätsklinik für Innere Medizin mit Schwerpunkt Pneumologie, Kepler Universitätsklinikum, 4020 Linz, Österreich; 15 Universitätsklinik für Neurologie der PMU, MME Universitätsklinikum Salzburg Christian-Doppler-Klinik, Ignaz-Harrer-Str. 79, 5020 Salzburg, Österreich; 16 Innere Medizin II, Medizinische Universität Innsbruck, Anichstr. 35, 6020 Innsbruck, Österreich; 17 Klinische Abteilung für Allgemeine Hals‑, Nasen- und Ohrenkrankheiten, Klin. Abteilung für Allgemeine HNO, Medizinische Universität Wien, Währinger Gürtel 18–20, 1090 Wien, Österreich; 18 Pyhrn-Eisenwurzen Klinikum Steyr, Sierninger Str. 170, 4400 Steyr, Österreich; 19 Universitätsklinik für Neurologie, Medizinische Universität Wien, Währinger Gürtel 18–20, 1090 Wien, Österreich; 20 Dermatologische Abteilung, Klinik Hietzing, Wolkersbergenstr. 1, 1130 Wien, Österreich; 21 Klinische Abteilung für Allgemeinpädiatrie, Universitätsklinik für Kinder- und Jugendheilkunde, Medizinische Universität Graz, 8036 Graz, Österreich; 22 Klinische Abteilung für Neurologie, Universitätsklinikum Tulln, Karl Landsteiner Privatuniversität für Gesundheitswissenschaften, Alter Ziegelweg 10, 3430 Tulln an der Donau, Österreich; 23 Institut für Pathophysiologie und Allergieforschung Zentrum für Pathophysiologie, Infektiologie und Immunologie, Medizinische Universität Wien, Währinger Gürtel 18–20, 1090 Wien, Österreich; 24 Klinische Abteilung für Pulmologie, Medizinische Universität Wien, Währinger Gürtel 18–20, 1090 Wien, Österreich; 25 Klinische Abteilung für Sozialpsychiatrie, Medizinische Universität Wien, Währinger Gürtel 18–20, 1090 Wien, Österreich; 26 Kardiologische Abteilung Klinikum Wels-Grieskirchen, Grieskirchnerstr. 42, 4600 Wels, Österreich; 27 Ärztin für Allgemeinmedizin, 8046 Graz, Österreich; 28 Ludwig Boltzmann Institute for Rehabilitation Research, Kurbadstr. 14, 1100 Wien, Österreich

**Keywords:** Postinfektiöse Zustände, Langzeitfolgen, Infektionskrankheiten, Anhaltende Symptome, Spätfolgen, Post-infectious condition, Long term sequelae, Infectious diseases, Persisting symptoms, Long term consequences

## Abstract

Die vorliegende Leitlinie S1 ist die Aktualisierung und Weiterentwicklung der Leitlinie S1 Long COVID: Differenzialdiagnostik und Behandlungsstrategien. Sie fasst den Stand der Kenntnis zu postviralen Zuständen anhand des Beispiels Long/Post COVID zum Zeitpunkt des Redaktionsschlusses zusammen. Aufgrund der starken Dynamik der Wissensentwicklung versteht sie sich als „living guideline“. Der Schwerpunkt liegt auf der praktischen Anwendbarkeit auf der Ebene der hausärztlichen Primärversorgung, die als geeignete Stelle für den Erstzutritt und für die primäre Betreuung und Behandlung verstanden wird. Die Leitlinie gibt Empfehlungen zum Versorgungsgang, zu Differenzialdiagnostik der häufigsten Symptome, die in der Folge einer Infektion wie mit SARS-CoV‑2 auftreten können, zu therapeutischen Optionen, zu Patient:innenführung und -betreuung sowie zur Wiedereingliederung in den Alltag und zur Rehabilitation. Entsprechend des Krankheitsbildes ist die Leitlinie in einem interdisziplinären und interprofessionellen Prozess entstanden und gibt Empfehlungen zu Schnittstellen und Kooperationsmöglichkeiten.

## Abstract (en)

These S1 guidelines are an updated and expanded version of the S1 guidelines on long COVID differential diagnostic and management strategies. They summarize the state of knowledge on postviral conditions like long/post COVID at the time of writing. Due to the dynamic nature of knowledge development, they are intended to be “living guidelines”. The focus is on practical applicability at the level of primary care, which is understood to be the appropriate place for initial access and for primary care and treatment. The guidelines provide recommendations on the course of treatment, differential diagnostics of the most common symptoms that can result from infections like with SARS-CoV-2, treatment options, patient management and care, reintegration and rehabilitation. The guidelines have been developed through an interdisciplinary and interprofessional process and provide recommendations on interfaces and possibilities for collaboration.

Die vorliegende Neufassung ist eine umfassende Aktualisierung und Erweiterung. Neue wissenschaftliche Erkenntnisse sowie klinische Erfahrungen wurden eingearbeitet, weitere Änderungen erfolgten aufgrund veränderter gesetzlicher Hintergründe (z. B. Wegfall von Quarantänemaßnahmen).

1. Titel: Ausdehnung des Begriffs auf die übergeordnete Gruppe der postviralen Syndrome
2. Zielsetzung:
  Die Erstfassung konzentrierte sich weitgehend auf Differenzialdiagnostik von unspezifischen Symptomen nach einer Infektion mit SARS-CoV‑2 und das Management dieser Symptome innerhalb der ersten 12 Wochen nach Erkrankungsbeginn. Die vorliegende Aktualisierung beschreibt zusätzlich das Management spezifischer postviraler Zustände im Gefolge von COVID-19 über diesen Zeitraum hinaus und schlägt ein Abklärungs- und Betreuungsschema entsprechend den beteiligten Ebenen des Gesundheitssystems vor. Ziele und Nicht-Ziele wurden dementsprechend angepasst und präzisiert.
3. Eine Präambel mit Begriffseinordnung wurde vorangestellt, derzeit gebräuchliche Definitionen wurden ergänzt.
4. Im Bereich der gesamten Leitlinie wurden Hinweise zu Diagnostik und Management aus Sicht der Ergotherapie im Sinne einer integrierten Betreuung ergänzt.
5. Änderungen im Bereich Kapitel 8:
  - Ergänzende neue Unterkapitel: 8.8 Immunologie, 8.9 Postvirale Fatigue, 8.10 Dysfunktionen des autonomen Nervensystems
  - Unterkapitel 8.5 Dermatologie: keine Änderungen, 8.7 Kinder, 8.4 HNO: Aktualisierung der Datenlage
  - Unterkapitel 8.1 Pneumologie: Änderungen residuale Pneumonie; Veränderungen kl. Atemwege und Dysfunktionale Atmung neu
  - Unterkapitel 8.2 Kardiologie: Literatur aktualisiert, 8.2.2 Kardiologische Symptome im Zusammenhang mit COVID-19: Quantifizierung „klassischer“ Herzerkrankungen nach COVID-19 und deren Abgrenzung von nicht näher zuordenbaren Symptomen neu
  - Unterkapitel 8.3 Neurologie: Überarbeitung Pathophysiologie, Aufnahme Kopfschmerz als neurologisches Symptom (8.3.3, Differenzialdiagnostik 11.3).
  - Unterkapitel 8.6 Psychiatrie: Diagnostische und therapeutische Aspekte wurden im Psychiatriekapitel differenziert bearbeitet, und der Satz wurde eingefügt, dass Körper und Seele eine lebendige Einheit darstellen
6. Neu: Kapitel 9 Versorgungsweg
7. Kapitel Follow-up und Monitoring der Erstfassung: aufgegangen in Kapitel 9 Versorgungsweg der Neufassung
8. Kapitel 10 Grundlagen der Differenzialdiagnostik ergänzt durch:
  - Typische Symptome postviraler Zustände
  - Tabellarische Auflistung verfügbarer Skalen und Scores
9. Kapitel 11 Symptombezogene Abklärungsgänge
  - Umfassende Überarbeitung von 11.1, 11.7, 11.8 und 11.9
  - Neu: 11.3 Kopfschmerz
10. Kapitel 12 Behandlung
  - Informationen zu nicht-medikamentösen und medikamentösen Therapieformen ergänzt
  - Aktivierende Therapieformen im Vergleich zur Originalfassung stärker differenziert und beschrieben
  - Spezifische therapeutische Optionen für Symptome postviraler Zustände nach COVID-19 explizit beschrieben
11. Leitfaden Ergotherapie
  Diese Leitlinie wird durch einen ergotherapeutischen Leitfaden, approbiert durch Ergotherapie Austria, ergänzt, der parallel publiziert wird [1]. Dies wird der Notwendigkeit eines interprofessionellen Zugangs beim Management der Erkrankung gerecht und ermöglicht allen Beteiligten Einblick in die Arbeitsfelder und Aufgaben der jeweiligen Berufsgruppen.

## 1. Einleitung

### 1.1 Präambel

Nach COVID-19 können, wie nach einer Reihe anderer viraler Erkrankungen auch, anhaltende Folgezustände auftreten. Die Symptome sind vielfältig und variabel. Diese Folgezustände können sowohl nach schweren als auch nach sehr milden und moderaten Verläufen auftreten. Sie bestehen über einige Wochen bis hin zu vielen Monaten und manche wohl auch Jahre. Die Beschwerden können persistierend sein, oder aber rezidivierend, undulierend oder neu aufgetreten [2–4]. Davon abzugrenzen sind Krankheitserscheinungen als Ausdruck organisch-struktureller Veränderungen nach schwerer Erkrankung, aufgrund der Verschlechterung einer vorbestehenden Krankheit oder als neu aufgetretene Erkrankung mit oder ohne Kausalzusammenhang mit der Viruserkrankung.

Die beschriebenen Folgezustände von COVID-19 sind auch von anderen viralen Erkrankungen bekannt. Dieser Tatsache wurde mit der Erweiterung des Leitlinientitels für die Überarbeitung Rechnung getragen.

Zur Historie des Begriffs: anhaltende Symptome nach COVID-19 wurden ab März 2020 als „Long COVID“ bezeichnet, basierend auf einem Twitter-Hashtag der an COVID-Folgezuständen leidenden Elisa Perego [5]. Dieses Hashtag wurde sehr rasch als Patientenbegriff auf sozialen Medien und in der Presse übernommen. Parallel dazu entwickelte sich auf Facebook der Patientenbegriff „Long Haulers“ [6]. Später kam der Terminus Post COVID hinzu, der ebenfalls keine Krankheitsentität beschreibt. Diese Leitlinie ordnet die mit diesen Begriffen beschriebenen Symptomkomplexe, der Ätiologie entsprechend, den postviralen Zustandsbildern zu, verwendet aber aus Gründen der Verständlichkeit immer wieder die gewachsene rein deskriptive Begrifflichkeit Long/Post COVID, die ja auch in Leitlinien längst Eingang gefunden hat (s. dazu auch den Abschnitt Definition, Kap. 4). Es muss darauf hingewiesen werden, dass dieser Begriff per se einen lediglich deskriptiven Charakter hat und nicht als Endpunkt eines diagnostischen Prozesses zu verstehen ist, sondern als Startpunkt für einen solchen.

### 1.2 Allgemeines

Die postviralen Zustandsbilder nach COVID-19 haben nach derzeitigem Kenntnisstand keinen einheitlichen pathogenetischen Hintergrund. Es gibt daher auch keinen validierten Biomarker und kein Testverfahren für diesen Oberbegriff unterschiedlicher Krankheitsbilder und Syndrome, wohl aber für gewisse Subgruppen. Aus diesem Grund müssen die berichteten Symptome(nkomplexe) individuell eingeordnet und – wo immer möglich – bekannten Symptomkomplexen zugeordnet werden, wobei v. a. auch alternative Erklärungen ausgeschlossen werden müssen. Wenn dieser Ausschluss nicht oder nicht ausreichend sorgfältig stattfindet, ist mit einer falschen Zuordnung zu rechnen [7]. Die Symptomatik ist meist unspezifisch und mehrdeutig.

Sinnvollerweise erfolgen Diagnostik, Behandlung und kontinuierliche Betreuung daher durch Generalist:innen in hausärztlicher Funktion. Die Einbindung von und Kooperation mit den Spezialist:innen der relevanten Fachgebiete sowie Angehörigen der Gesundheits- und Sozialberufe ist sowohl in der Diagnostik als auch in Therapie und Patient:innenführung häufig nötig und/oder sinnvoll.

Eine deutliche spontane Abnahme der Symptomatik im Laufe der Zeit wird beobachtet [8]. Aktuelle Studien und ein Review mit Metaanalyse zeigen, dass etwa die Hälfte der Symptome nach 4 Monaten und ca. drei Viertel der Symptome nach 15 Monaten verschwunden sind [9, 10]. Damit resultieren aber auch, angesichts der sehr hohen Fallzahlen, viele Patient:innen mit persistierenden Beschwerden und anhaltendem Behandlungs- bzw. Betreuungsbedarf.

Multiprofessionelles und multidisziplinäres Zusammenwirken entsprechend einem individualisierten Behandlungsplan sind essenziell [11].

## 2. Zielsetzung der Leitlinie

A. Überblick über die bisherigen Erkenntnisse zu Folgezuständen nach COVID, geordnet nach Organsystemen bzw. funktionellen Einheiten
B. Beschreibung der Versorgungswege (Erstabklärung, Rehabilitation, Zuordnung der Aufgaben an die jeweiligen Berufsgruppen)
C. Leitung durch die primäre Abklärung und Zuordnung der mehrdeutigen Symptomatik, wobei der typischen hausärztlichen Vorgangsweise bei der Differenzialdiagnostik gefolgt wird. Zielsetzung dabei ist:
  - Ausschluss/Abklärung von Erkrankungen aus anderer Ursache
  - Erkennen von organisch-strukturellen Ursachen als kurz-, mittel- oder langfristige Folge der SARS-CoV-2-Infektion, der akuten COVID-19-Erkrankung und/oder ihrer Komplikationen
  - Erkennen einer Verschlechterung vorbestehender Grundkrankheiten im Gefolge von COVID-19
  - Abgrenzung anhaltender Störungen durch postvirale Folgezustände im Körper nach SARS-CoV-2-Infektion von organisch-strukturellen Ursachen
D. Empfehlungen zur Behandlung der zugeordneten Störungen und Beschwerden
E. Empfehlungen zu Betreuung und Coping
F. Empfehlungen zur Vermeidung iatrogener Verstärkung
G. Empfehlungen zur Vermeidung von Chronifizierung sowie
H. Empfehlungen zu Rehabilitationsbedarf und -optionen

In dieser Leitlinie nicht umfassend behandelt werden konnten folgende Themen:

A. Das Post-Intensive-Care-Syndrome (PICS)
B. Pathogenese, Diagnostik und Management des als ME/CFS beschriebenen Zustandsbildes: Diese können aufgrund ihrer hohen Komplexität in dieser Leitlinie nicht umfassend abgehandelt werden. Zu diesem Thema empfiehlt die Leitliniengruppe insbesondere für den niedergelassenen Bereich, die Leitlinie S3 Müdigkeit heranzuziehen, die ein ausführliches Kapitel zu diesem Thema enthält [12]. Wir weisen zudem auf die Leitlinie der AWMF hin, die dazu einen Abschnitt enthält [13], sowie auf die Leitlinie der NICE (National Institute for Health and Care Excellence) zu ME/CFS [14].
C. Medikamentöse in Erprobung befindliche Therapieoptionen werden berichtet, es werden aber keine Empfehlungen oder Nicht-Empfehlungen, soweit nicht in Studien belegbar, abgegeben.

## 3. Aufbau

1. Kapitel 1–8: Grundlagenwissen über postvirale Zustandsbilder nach COVID-19 („Long COVID/Post COVID“):
  Definition und Bedeutung, Charakteristika (1–6),
  Pathomechanismen (7),
  Organsysteme, funktionelle Komplexe: Auswirkungen von COVID-19 und spezifische Folgen (8)
2. Kapitel 9: Versorgungswege und Zuständigkeiten
3. Kapitel 10–11: Diagnostik von Symptomen nach einer Infektion mit COVID-19
4. Kapitel 12–13: Betreuung Begleitung, Behandlung, Rehabilitation

## 4. Definition „Long COVID“

Eine Vereinheitlichung der Terminologie bzw. eine Klassifizierung sind bisher noch nicht erreicht. In vielen Publikationen werden unterschiedliche Symptome im Gefolge von COVID-19 unter dem Begriff „Long COVID“ und/oder Post COVID gefasst, ohne dass eine ätiologische Zuordnung erfolgt: Dazu zählen Folgen schwerer Akuterkrankung und deren Komplikationen, Verschlechterung vorbestehender Grundkrankheiten, fortbestehende Symptome der Erkrankung selbst bzw. nicht zuordenbare Folgebeschwerden aus nicht vollständig geklärten Pathomechanismen und neu aufgetretene Erkrankungen [15–17]. Andere schränken den Begriff stärker auf diejenigen Symptome ein, die klinisch dem Krankheitsbild bei COVID-19 zuordenbar sind [18] und nicht mit einer alternativen Diagnose erklärt werden können und/oder organisch-strukturelle Folge schwerer Erkrankung [19, 20] sind.

Seit Oktober 2021 existiert ein ICD-10-Code („International Statistical Classification of Diseases and Related Health Problems“; U09.9, post COVID-19 condition, nicht näher bezeichnet), der rein deskriptiv und daher sehr umfassend ist.

**Figure 1:**
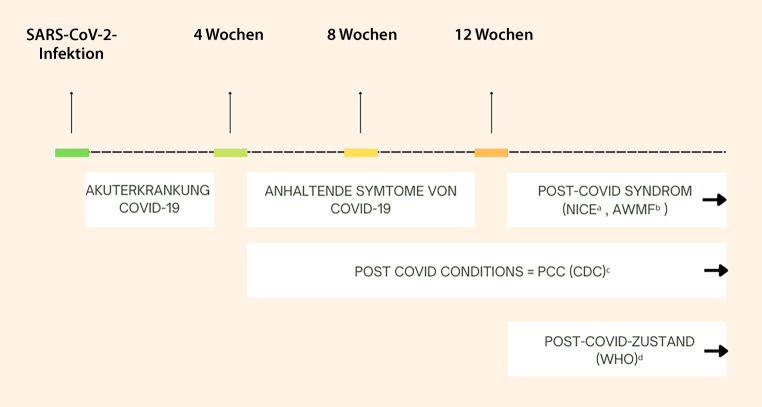

Es existieren derzeit einige unterschiedliche Definitionen parallel (Überblick Abb. 1). Die Autor:innengruppe hat sich entschieden, im Rahmen dieser Leitlinie der Definition von NICE (National Institute for Health and Care Excellence) zu folgen, da sich die vorliegende Leitlinie vor allem mit der Differenzialdiagnostik von Symptomen befasst, die situationsabhängig schon vor Ablauf der WHO-Zeitgrenze (2 Monate) einsetzen sollte.

Terminologie der NICE-Leitlinie [18]:

- Akuterkrankung **COVID-19**: Befunde und Symptome von COVID-19 bis zu 4 Wochen
- Anhaltende **Symptome von COVID-19**: 4 bis 12 Wochen
- **Post-COVID-Syndrom**: Befunde und Symptome, die während oder nach einer Infektion mit SARS-CoV‑2 entstehen und zu den bei COVID-19 beobachteten Symptomen passen, die mehr als 12 Wochen bestehen und bei denen keine andere erkennbare Ursache vorliegt

### Weitere wichtige und häufig verwendete Definitionen

**WHO**^19^ Definition für **Post-COVID Condition (in Folge als „Post-COVID-Zustand“ übersetzt)**, die in einem globalen Delphi-Konsensusprozess erzielt wurde

- in der Regel Manifestation bis 3 Monate nach Beginn der COVID-19/SARS-CoV-2-Infektion
- Symptome, die über mindestens 2 Monate bestehen, nicht durch eine andere Diagnose erklärt werden können
- Symptome haben generell Bedeutung für die Alltagsfunktion der Betroffenen
- Symptome, die evtl. nach initialer Erholung von der akuten COVID-19-Episode aufgetreten sind oder seit der initialen Erkrankung persistieren
- Symptome, die fluktuieren oder im Verlauf rezidivieren

**CDC** verwendet die Termini Long COVID und Post-Covid Conditions (PCC) simultan für alle Symptome, die mit einer Erkrankung an COVID-19 vor zumindest 4 Wochen assoziiert sind ohne zeitliche Abgrenzung [21].

Die Leitlinie der AWMF führt zusätzlich 4 Subtypen für Post-COVID-Patient:innen ein [13].

Entsprechend den uneinheitlichen Begriffen in der Literatur werden in dieser Leitlinie mehrere der gängigen Begriffe synonym verwendet:

Anhaltende Symptome von COVID-19, anhaltende Folgen von COVID-19, Post-COVID-Zustand (PCC – Post COVID Condition), postvirales Zustandsbild nach COVID-19, Post COVID, Post-COVID-Syndrom, Long COVID, postviraler Zustand nach COVID. „post-acute sequelae of COVID-19“ (PASC), „chronisches COVID Syndrom (CCS)“ oder „COVID-19 long-hauler“.

## 5. Epidemiologie und Bedeutung

Eine Quantifizierung des Problems ist bisher nicht gelungen – die Angaben für die Häufigkeit von Long COVID in der derzeit verfügbaren Literatur schwanken zwischen den Extremwerten von 2,3 % [23] und über 80 % [24–26]. Eine englische Metaanalyse [27] zeigt eine äußerst niedrige Prävalenz von nur 0,3 % unter Personen nach COVID-19 innerhalb der Primärversorgung.

Diese hohe Variabilität ist mit großer Wahrscheinlichkeit auf die Verwendung der unterschiedlichen Definitionskriterien, Beobachtungsgruppen und angewendeten Methoden der Erhebungen zurückzuführen, wohl auch auf eine sehr unterschiedliche Erfassung [27], s. dazu die umfassende Evidenzsynthese des Robert Koch-Instituts (RKI) [28, 29].

Viele Studien haben beispielsweise ihre Daten an unterschiedlichen Kollektiven erhoben (zuvor hospitalisierte Personen, nicht-hospitalisierte und gemischte Samples) und zu sehr unterschiedlichen Zeitpunkten (zwischen 3 Wochen und 9 Monaten nach Infektion), häufig fehlen Kontrollgruppen. Die Datenerfassung erstreckte sich über sehr unterschiedlich lange Zeiträume [3, 30], die Methodik der Erhebung (App, Health Records, elektronische Surveys, Interviews) ist ebenfalls derart unterschiedlich, dass Vergleiche kaum möglich und Verzerrungen häufig sind. Ein ICD-10-Code wurde erst im Herbst 2021 eingeführt, die Codierungsqualität ist zudem uneinheitlich. Große Studien registrieren meist selbst berichtete Symptome ohne Nachweis einer stattgehabten Infektion mit SARS-CoV‑2 und ohne Vergleich mit einer gesichert nicht infizierten Bevölkerung. Die globale Prävalenz wird in einem Systematic Review mit 43 % angegeben (54 % nur für Hospitalisierte) [25]. Eine Studie, die nach der Symptomatik vor und nach COVID-19 beim gleichen Individuum korrigiert, findet dagegen nur eine Häufigkeit von 12,7 % [31]. Grundsätzlich leiden die existierenden Vergleichsstudien zudem meist an den unzulänglichen Testkriterien, die zu Verzerrungen vor allem in Bezug auf falsch negative Personen in den Kontrollgruppen führen [32, 33]. Dies führt auch zu erheblichen Limitierungen für Systematic Reviews, die an der sehr unterschiedlichen Methodik und Qualität der eingeschlossenen Studien leiden [27, 34]. Mehrere Studien, die berichtete Symptome zwischen seronegativen und seropositiven Individuen vergleichen, finden keine Unterschiede [35–37]. Auch bei diesen Studien bestehen methodische Probleme, z. B. ist die Dauer der Persistenz der jeweils zum Nachweis verwendeten Antikörper nicht geklärt [38].

Eine wesentliche Problematik ist die terminologische Unschärfe. Zwischen Symptomen aus alternativer Ursache, organischen Folgen schwerer Erkrankung (inklusive Post-Intensive-Care-Syndrom), und postviralen Zuständen, wie sie auch als Folge anderer Infektionserkrankungen (wie EBV, CMV, Parvovirus, Influenza u. a.) bekannt sind, wird selten eine klare Trennung vollzogen, trotz der völlig unterschiedlichen Ätiologie.

Zudem dürfte eine deutliche Abhängigkeit von der verursachenden Virusvariante bestehen – die allerdings in ihrer Bedeutung von der veränderten Immunitätslage in der Bevölkerung kaum zu trennen sein wird. Die meisten rezenteren Studien finden für frühere Varianten von Wildvirus bis Deltavariante Häufigkeiten um 10 % [31, 39, 40]. Daten aus der britischen Symptom-Study-App zeigen in etwa eine Risikoreduktion der Inzidenz von Folgezuständen um 25–50 % bei Omikron BA 1 und 2 (abhängig von Alter und Impfstatus) gegenüber der Deltavariante [40, 41]. Demgegenüber steht eine Studie, in welcher gezeigt wurde, dass die neurologischen und psychiatrischen Long-COVID-Symptome durch Omikron im Vergleich zu Delta nicht verringert waren [42].

Zu beachten ist jedoch, dass die Omikron-Welle mit einem starken Anstieg der Infiziertenzahlen einherging, wobei die Infektionen generell weniger schwer verliefen und es de facto nicht mehr zu Hyperinflammationssyndromen oder schwerem Lungenversagen kam. Eine zunehmende Herausforderung bringt auch die Einschätzung des Risikos nach 2 und mehr Infektionen. Ob Reinfektionen das Risiko für Post COVID erhöhen können, ist Gegenstand aktueller Studien [43], tendenziell zeigt sich eine Zunahme des Risikos für Long COVID und Langzeitschäden.

Welche Personen ein erhöhtes Risiko haben, anhaltende Symptome, die unter dem Begriff Long COVID zusammengefasst sind, nach SARS-CoV-2-Infektion zu entwickeln, ist nicht geklärt. Unter anderem werden auch genetische Faktoren diskutiert [15]. Für zuvor hospitalisierte und schwer erkrankte Personen dürfte die Wahrscheinlichkeit höher sein [44–46], was wenig überraschend erscheint, da Organschäden vornehmlich Folge schwerer Verläufe sind. Hier wird es in Zukunft hoffentlich möglich sein, Long-COVID-Symptome durch unterschiedliche Ursachen voneinander abzugrenzen und getrennt zu untersuchen (z. B. Symptome durch Organschäden vs. funktionelle Störungen vs. Symptome durch postvirale Zustände).

Gesicherte Erkenntnisse zu Prädiktoren für die Entwicklung anhaltender Symptome nach COVID-19 gibt es nicht. Eine Metaanalyse [46] zeigt ein erhöhtes Risiko für die Entwicklung anhaltender Symptome nach COVID-19 für Alter, weibliches Geschlecht, Rauchen, Komorbiditäten und Hospitalisierung während der akuten Krankheitsphase sowie eine Risikoreduktion nach zumindest 2‑maliger Impfung. Auch diese Übersicht leidet an den oben diskutierten Limitationen. Des Weiteren werden diskutiert:

- Symptomanzahl während der akuten Krankheitsphase [8, 47, 48], ausgeprägte Fatigue während der Akuterkrankung [47, 48]
- Hohe Viruslast zu Beginn [49]
- Auffälliges Labor während der akuten Infektion (erniedrigtes Gesamt-IgM und IgG3 und erhöhtes S1-spezifisches IgG, erhöhtes D‑Dimer, derangiertes Koagulopathieprofil) [50, 51]
- Waning Immunity/nicht vorhandene Immunität [40]
- Genetische Faktoren, Immunopathien
- Soziale Deprivation
- Vorbestehende Autoimmunerkrankungen
- Vorbestehende Beeinträchtigungen der mentalen Gesundheit/verminderte Resilienz
- Fehlende oder nicht ausreichende Rekonvaleszenz nach SARS-CoV-2-Infektion [52–54]
- Re-Aktivierung von bereits im Körper persistierenden Viren, wie z. B. EBV [49]

Aktuelle Studien und ein Review mit Metaanalyse zeigen, dass etwa die Hälfte der Symptome nach 4 Monaten und ca. drei Viertel der Symptome nach 15 Monaten ganz verschwunden sind [10, 39].

Indirekte Schlüsse auf die epidemiologische Bedeutung können eventuell aus Zahlen zur Beeinträchtigung der Arbeitsfähigkeit gezogen werden [55–57].

Organische und auch funktionelle Störungen im Gefolge von Infektionskrankheiten sind keine für SARS-CoV‑2 spezifische Phänomene. Die Bedeutung von Long COVID für Gesellschaft und Gesundheitssystem liegt vor allem darin, dass während der Pandemie eine große Zahl von Personen gleichzeitig erkrankt ist.

## 6. Symptomatik

Die Symptomatik der unter dem Patient:innenbegriff Long COVID zusammengefassten Erscheinungen ist inter- und intraindividuell sehr variabel, der Schweregrad reicht von Störungen des Befindens bis zu massiver Einschränkung des alltäglichen Lebens. Ein Teil der Patient:innen erholt sich nach der SARS-CoV-2-Infektion über Wochen bis Monate nicht oder erfährt Rückfälle. Möglich sind persistierende Beschwerden ebenso wie eine undulierende Symptomatik. Bekannt ist auch ein Wieder- oder Neuauftreten von Symptomen nach einem beschwerdefreien Intervall.

Entsprechend den diskutierten Pathomechanismen der Erkrankung sind auch deren mögliche Späterscheinungen sehr vielfältig und können unterschiedliche Ursachen haben und ganz unterschiedliche Organsysteme betreffen [4, 58–65].

Symptome und deren Häufigkeiten werden in der Literatur ebenfalls äußerst variabel angegeben und wurden in sehr unterschiedlichen Populationen untersucht. Immer deutlicher wird jedoch, dass Personen nach COVID-19 Unterschiede in Art und Schwere der Symptomatik zeigen im Vergleich mit solchen, die mit respiratorischen Symptomen negativ auf SARS-CoV‑2 getestet wurden [39].

Dazu kommen die im Kapitel Epidemiologie bereits dargestellten methodischen Schwierigkeiten, die auch auf unzulänglichen Testungskriterien mit zu eng gestellter Indikation beruhen. Viele Personen wurden trotz Symptomatik nicht getestet (da diese nicht den zu engen Kriterien entsprach), andere Studien inkludieren klinische COVID-19-Diagnosen [31–33]. Daher sind alle Vergleichsstudien ohne zumindest serologischen COVID-19-Ausschluss eingeschränkt zuverlässig.

Als am häufigsten berichtete Symptome finden sich in einem Systematic Review [25, 30] und in bevölkerungsbezogenen Erhebungen folgende:

- Müdigkeit, Erschöpfung, eingeschränkte Leistungsfähigkeit bis hin zur Leistungs‑/Bewegungsintoleranz (Post-Exertional-Malaise), Schwäche (unterschiedlicher Schweregrade, bis zu Fatigue-Syndrom),
- anhaltender Verlust des Riech- und/oder Schmeckvermögens (variantenabhängig),
- Atembeschwerden, Kurzatmigkeit, Belastungsdyspnoe,
- Insomnie,
- Konzentrationsstörung, Gedächtnisstörung,
- Tachykardie, Dizziness, Symptome einer orthostatischen Dysregulation,
- Brustschmerzen oder Brustenge,
- Husten,
- Gelenk- und/oder Muskelschmerzen,
- Kopfschmerz,
- Nervenschmerzen und andere Missempfindungen, beschrieben auch als „pins and needles and numbness“,
- Haarausfall und sexuelle Dysfunktion [66].

Eine prospektive Beobachtungsstudie mit 76.000 Proband:innen und mehreren Erhebungspunkten über 90 bis 150 Tage findet eine deutlich höhere Prävalenz von Brustschmerz und Atembeschwerden im Gefolge der COVID-19-Infektion, verglichen mit der Zeit vor der Erkrankung [31]. Dieselbe Studie zeigt auch auf, dass der Verlauf von unterschiedlichen Symptomen sich über die Zeit unterschiedlich entwickeln kann: So nehmen Symptome wie Geruchsstörungen und Husten über die Zeit eher ab, Symptome wie Schmerzen und Fatigue eher zu [31].

Weitere Symptome sind [8, 18, 67–70]: persistierende Rhinitis, Symptome der autonomen Dysfunktion, wie z. B. Sicca-Symptomatik, verminderter Appetit, Schwitzen, intermittierende (sub)febrile Körpertemperatur und Durchfall, Haarausfall, depressive Verstimmung, Angststörung, Hautausschläge, neu aufgetretene Allergien und Unverträglichkeiten, neurogene Blase.

In einer landesweiten Umfrage in Dänemark bestanden bei knapp 30 % der Befragten 6 bis 12 Monate nach positivem SARS-CoV-2-Test Symptome im Vergleich zu 13 % der Befragten mit einem negativen Test. Folgende Symptome traten vermehrt auf (in der Reihenfolge der Häufigkeit im Vergleich zu Proband:innen mit einem negativen Test): Dysosmie, Dysgeusie, Fatigue/Erschöpfung, Dyspnoe, verminderte Kraft in Armen und Beinen. Das Risiko für Symptome war bei Frauen und bei Personen im mittleren Alter erhöht [71].

Bei Patient:innen, die länger als 6 Monate an Long-COVID-Symptomen litten, standen Fatigue, Post-Exertional-Malaise und kognitive sowie autonome Dysfunktionen im Vordergrund [4].

## 7. Pathomechanismen – Was ist bekannt?

Die konkrete Pathogenese der unterschiedlichen Symptome und Symptomenkomplexe bei anhaltenden Zuständen nach COVID-19 ist derzeit noch nicht vollständig geklärt. Sie ist jedenfalls multifaktoriell und auch nicht bei allen Personen ident. In Erwägung gezogene Auslöser sind lang dauernde Gewebeschäden, eine Persistenz von Viren oder zumindest Virusbestandteilen sowie eine chronische (Hyper‑/Auto‑)Inflammation. Darüber hinaus wird eine Reaktivierung bereits vorbestehender persistierender Infektionen wie HSV1/2, EBV [72–74] oder Varizella-Zoster diskutiert. Auch genetische Ursachen, die für die Entwicklung der Symptome prädisponieren, werden in Erwägung gezogen [75].

SARS-CoV‑2 ist nicht einzigartig in seiner Fähigkeit, postakute Folgen zu verursachen. Bestimmte akute Infektionen werden bereits seit Langem mit chronischen Beeinträchtigungen in Verbindung gebracht [76], nachdem die Long-COVID-Symptome eine große Ähnlichkeit mit diesen bekannten Beeinträchtigungen haben, werden auch eine gemeinsame Ätiologie und Pathogenese vermutet, welche Choutka et al. in ihrer Publikation in *Nature Medicine* beschreiben [76]:

1. Pathogen(reste) oder -reservoire verbleiben im Körper und führen zu chronisch stimulierten T‑ und B‑Lymphozyten und erhöhen das Risiko für chronisch entzündliche Zustände im Körper/Gewebe.
2. Pathogen führt durch molekulares Mimikry oder deregulierte T_reg_-Zellen zu autoreaktiven Lymphozyten und autoreaktiven Antikörpern.
3. Pathogen führt zu einer Dysregulation der Mikrobiota. Über die Darm-Hirn-Achse können durch die Dysbiose Affektionen des Vagus, Re-Aktivierung latenter Viren und/oder Mikrogliaaktivierung resultieren.
4. Permanente chronische Gewebeschädigung durch das Pathogen oder durch dieses ausgelöste entzündliche/autoreaktive Reaktionen.
5. Alle oben beschriebenen Faktoren können direkt oder indirekt zelluläre und systemische metabolische Prozesse, Neurotransmittersynthese oder die Mitochondrienfunktion beeinflussen bzw. vice versa.

Diese Prozesse schließen sich nicht gegenseitig aus und könnten in verschiedenen Untergruppen in Kombination existieren oder mit unterschiedlicher Intensität ausgeprägt sein.

Diese Ursachen werden auch in einem systematischen Review [77] und einem EC-Bericht [34] als zugrunde liegend beschrieben.

Bei Patient:innen mit Symptompersistenz über ein Jahr nach Akutinfektion fanden sich in einer Studie, welche sich für die Phänotypisierung des „machine learning“ bediente, entsprechend dem oben beschriebenen Modell zahlreiche Immunaberrationen, darunter hohe Konzentrationen von Monozyten, funktionsbeeinträchtigte T‑Zellen und das Vorhandensein doppelt positiver CD4^+^- und CD8^+^-T-Zellen. Hohe Konzentrationen von Anti-Coronavirus-Antikörpern deuteten darauf hin, dass das Immunsystem der Patient:innen auch über ein Jahr nach Infektion noch auf das Virus reagierte – entweder weil es selbst oder Teile davon noch vorhanden waren oder weil eine Autoimmun- oder Entzündungsreaktion bestehen blieb. Das SARS-CoV‑2 war nicht das einzige Virus, auf das das Immunsystem reagierte – hohe Antikörperspiegel und Aktivierungsmarker deuteten darauf hin, dass Herpesviren, insbesondere EBV und Varizella-Zoster, reaktiviert wurden. Der Faktor, der die Patient:innen jedoch am deutlichsten von den gesunden Kontrollen unterschied, war ein niedriger Cortisolspiegel ohne erhöhtes ACTH [78].

Eine verminderte Aktivität der Stresshormonachse [79] könnte eine gewisse Erschöpfung erklären, denn niedrige Stresshormonlevel können einerseits dazu führen, dass Entzündungsreaktionen nicht gebremst werden und andererseits niedrigen Blutdruck und Kreislaufbeschwerden verursachen. Aus diesem Grund kommen auch die HPA(Hypothalamus-Hypophysen-Nebennierenrinde)-Achse und Cortisol [80] immer mehr in den Fokus des Interesses.

Die Cortisol-Regulierung der Stressreaktion, des Stoffwechsels, des Schlaf-Wach-Rhythmus und der Unterdrückung von Entzündungen deutet darauf hin, dass sie das Potenzial hat, viele der Prozesse zu beeinflussen, die bei von Long-COVID-Betroffenen gestört sind [81].

Die gefundenen immunologischen Aberrationen [78, 82] könnten mit Symptomen der immunologischen Dysfunktionen in Verbindung gebracht werden [64]. Diskutiert wird, dass SARS-CoV‑2 in Antigen-präsentierenden Zellen eine „Bystander-Aktivierung“ von T‑Zellen gegen Autoantigene auslösen könnte [83]. Mögliche alternative oder weitere Ursache für eine Hyperinflammation könnten Veränderungen der Mikrobiota des Gastrointestinaltraktes [84] bzw. eine Dysbiose [85, 86] sein. Neben einer T‑Zell-vermittelten Autoimmunität finden sich auch Beobachtungen von Anti-Phospholipid-Autoantikörper [87] ebenso wie Autoantikörper gegen Zellkerne, Neutrophile, Interferone oder Citrullinpeptide. Es ist bekannt, dass solche Autoantikörper in der Pathogenese unterschiedlicher Autoimmunerkrankungen (SLE, rheumatoide Arthritis, Sjögren-Syndrom usw.) eine Rolle spielen können [88, 89].

Auch durch diese postviralen Vorgänge beeinflusste mitochondriale Dysfunktion [90] mit gestörtem Fettsäurestoffwechsel wird immer öfter bei Patien:innen mit PASC beschrieben.

Ein immer wichtiger werdender Pathomechanismus dürfte auch die durch oben beschriebene postvirale Vorgänge entstehende endotheliale Dysfunktion sein. Einerseits wird die Hypothese diskutiert, dass die endotheliale Dysfunktion Folge der fortbestehenden Endotheliitis durch SARS-CoV‑2 [91] ist, andere Hypothesen gehen eher in die Richtung, dass es durch die postviral ausgelösten chronischen Entzündungen und/oder Autoimmunreaktionen zu einer endothelialen Dysfunktion (Endotheliitis, Endothelialitis und Endotheliopathie) kommt [92–94].

Des Weiteren relevant ist eine Gerinnungsaktivierung bzw. ein prothrombotisches Milieu während der akuten COVID-19-Infektion. Auch über die Akutphase hinaus wurde eine erhöhte Expression von prothrombotischen Faktoren (Faktor VIII, Plasminogen-Aktivator-Inhibitor-1) beschrieben [95, 96]. Dies nimmt nach der akuten Infektion ab, kann aber zumindest für die Dauer eines Jahres persistieren. Maher et al. [97] fanden heraus, dass Monozyten ihr Genexpressionsprofil ändern und zwar von kanonischen angeborenen Immunsignaturen zu prothrombotischen Signaturen und damit funktionell prothrombotisch werden und an Signalwegen zur Thrombozytenaktivierung und Gerinnselbildung beteiligt sein können.

In einer landesweiten Studie in England und Wales an 48 Mio. Erwachsenen [98] war das Risiko für eine arterielle thrombotische Erkrankung (meist Myokardinfarkt und Schlaganfall) in der ersten Woche nach der COVID-19-Diagnose massiv erhöht (Myokardinfarkt HR 17,2; Schlaganfall HR 23,0) und nahm in der Folge rasch ab. Allerdings persistierte ein etwas erhöhtes Risiko auch nach 13 bis 26 Wochen (Myokardinfarkt HR 1,17; Schlaganfall HR 1,58) sowie nach 27 bis 49 Wochen (Myokardinfarkt HR 1,21; Schlaganfall HR 1,62). Das Risiko für venöse Thromboembolien war ebenfalls in der ersten Woche nach der COVID-19-Diagnose stark erhöht (tiefe Beinvenenthrombose HR 10,8; Pulmonalembolie HR 33,2) und persistierte auf niedrigerem Niveau auch nach 13 bis 26 Wochen (tiefe Beinvenenthrombose HR 1,95; Pulmonalembolie HR 2,41) sowie nach 27 bis 49 Wochen (tiefe Beinvenenthrombose HR 1,99; Pulmonalembolie HR 1,61). Das Risiko war nach einer Hospitalisierung wegen COVID-19 höher als bei Patient:innen, die keiner Hospitalisierung bedurften.

Eine weitere Hypothese besagt, dass anhaltende Zustände nach COVID-19 mit einer chronischen subklinischen systemischen Entzündung (Inflammation) einhergehen könnten, wie dies im Alterungsprozess (Aging) beobachtbar ist. Dieses „Inflammaging“ hätte das Potenzial, bestehende Komorbiditäten zu verschlechtern und altersabhängige Probleme deutlich zu verstärken [99]. Eine Studie von Mongelli et al. fand einen konsistenten Anstieg des biologischen Alters in der betroffenen Bevölkerung und bestimmte einen Delta-Age-Anstieg von 10,45 ± 7,29 Jahren (+5,25 Jahre über dem Normalbereich) im Vergleich zu 3,68 ± 8,17 Jahren für die COVID-19-freie Bevölkerung (*p* < 0,0001). Eine signifikante Telomerverkürzung entspricht diesem Befund in der Kohorte mit PASC im Vergleich zu COVID-19-freien Proband:innen (*p* < 0,0001). Angesichts dieser Beobachtungen stellen sie die Hypothese auf, dass einige epigenetische Veränderungen mit dem Post-COVID-19-Zustand verbunden sind, insbesondere bei jüngeren Patient:innen (< 60 Jahre) [100].

Die als **„Zytokinsturm“** bezeichnete schwere, systemische Inflammation in der akuten Erkrankung und deren Folgen sind in allen Altersgruppen beobachtbar. Vielfach wurde bei Kindern eine schwere Multisysteminflammation mit Ähnlichkeiten zum Kawasaki-Syndrom beschrieben. Hält eine Entzündungsreaktion jedoch über lange Zeit an, so wird angenommen, dass dies zu zellulärer Seneszenz mit Hemmung der Zellproliferation und Resistenz gegenüber Apoptose führt [99].

Eine abschließende Klärung der Pathomechanismen steht jedoch auch hier noch aus. Siehe dazu auch Iwasaki [101].

### 7.1 Spezielle Aspekte

#### Pathophysiologie der COVID-19-Riechstörung (s. auch Abschn. 8.4 HNO)

Da die olfaktorischen Rezeptorneurone selbst kaum ACE-Rezeptoren besitzen, scheint die COVID-19-bedingte Riechstörung durch eine virusvermittelte Schädigung der Stützzellen der Riechschleimhaut zu entstehen, wodurch die oft nur kurzfristige Störung zu erklären wäre [102]. Eine schwerer wiegende Infektion könnte aber auch die bestehenden Riechnervenzellen irreversibel schädigen. Dadurch braucht die Regeneration des Riechvermögens, die von den Basalzellen der Riechschleimhaut ausgeht, zumindest mehrere Monate. Auch eine Schädigung von Riechzentren im Gehirn (z. B. Bulbus olfactorius) ist denkbar, jedoch als weniger wahrscheinlich anzunehmen [103].

- Die COVID-19-bedingte Riechstörung entsteht durch eine Schädigung der Stützzellen der Riechschleimhaut.
- Bei Schädigung der Riechnervenzellen kommt es zu einer langfristigen, evtl. dauerhaften Störung.
- Eine zentrale Schädigung (z. B. Bulbus olfactorius) gilt als weniger wahrscheinlich.

#### Pathophysiologie der neurologischen Störungen (s. auch Abschn. 8.3 Neurologie)

In einer Studie in einem tertiären neurologischen Versorgungszentrum für Long COVID konnten nur in einer geringen Anzahl von Patient:innen mit neurologischen Standarduntersuchungsmethoden Affektionen des Nervensystems gezeigt werden. Die Autor:innen der Studie spekulieren aus diesem Grund, dass eventuell auch entsprechende neuropsychologische Krankheitsmodifikatoren möglich wären. Auf der anderen Seite weisen sie in den Limitationen jedoch auch darauf hin, dass sich bei Hinzuziehen von weiteren Messmethoden auch andere Ergebnisse zeigen könnten [104]. Dem heutigen Stand entsprechend, ist SARS-CoV‑2 kein neurotropes Virus [105], entsprechende Auffälligkeiten im Gehirn dürften auf eine indirekte Entzündungsreaktion zurückzuführen sein [106].

### 7.2 Zusammenfassung

Zusammenfassend bestehen derzeit unterschiedliche Hypothesen zur Pathogenese des Post-COVID-Zustandes wie virale Persistenz und Reaktivierung, autoimmunologische Mechanismen, hormonelle Dysfunktion, Mikrobiom- und Metabolomveränderungen sowie endotheliale Störungen, die möglicherweise auch gleichzeitig auftreten könnten. Es könnten somit bei einer Person verschiedene Ursachen für die bestehenden Symptome zu finden sein, wodurch die weitere Beforschung der adäquaten Patient:innenstratifizierung herausfordernd ist. Eine Klärung der Ursache/n bei individuellen Patient:innen ist nach dem derzeitigen Kenntnisstand häufig nicht möglich.

#### Wichtig erscheint jedenfalls eine Abgrenzung von:

1. Folgen eines konkreten Organschadens durch die akute COVID-19-Erkrankung (z. B. Lungen- oder Nierenschädigung),
2. unspezifischen und spezifischen Folgen der Hospitalisation und/oder sozialer Isolation (von ernährungsbedingter Anämie bis hin zum Muskelabbau),
3. Symptomen von psychosozialer Belastung mit oder ohne Zusammenhang mit COVID-19,
4. Symptomen bedingt durch oben beschriebene postvirale Pathomechanismen [76]:
  A. Neuauftreten einer Erkrankung infolge einer SARS-CoV-2-Infektion (z. B. Risikoerhöhung für Diabetes [107], CHD, Schlaganfälle, erhöhtes perioperatives Risiko),
  B. Folgen durch eine infektionsbedingte Verschlechterung/Re-Aktivierung einer bereits vorher bestehenden, chronischen oder residualen Erkrankung-
  C. weitere Post-COVID-Symptomkomplexe.

Auch bedürfen alle in Folge geschilderten Symptome unabhängig von einer durchgemachten COVID-19-Erkrankung/SARS-CoV-2-Infektion einer differenzialdiagnostischen Abklärung, wie in Kapitel 10 beschrieben.

#### Empfehlung

Der ätiologische Hintergrund patientenseitig berichteter Symptome sollte jedenfalls soweit geklärt werden, dass zwischen organischen, mentalen oder sozialen Folgen durchgemachter schwerer Erkrankung an COVID-19, physischen oder mentalen Störungen alternativen Ursprungs, psychosozialen Pandemiefolgen und Symptomen aufgrund postviraler Pathomechanismen unterschieden werden kann.

## 8. Organsysteme – Übersicht: Leitsymptome und Krankheitsbilder

### 8.1 Pneumologie/Infektiologie

#### 8.1.1 Pneumologische Leitsymptome als Folge von COVID-19

##### Dyspnoe (s. auch 10.4 und 12.4.1)

- Dyspnoe im Rahmen von Long COVID äußert sich vor allem als **Kurzatmigkeit bei Belastung** und findet sich häufiger nach schwerem Verlauf (nach 3 Monaten noch in ca. 40 % [108], aber auch nach nicht hospitalisiertem Verlauf (in ca. 10 %) [8]). Eine milde Dyspnoe über einige Wochen nach der Akuterkrankung wird häufig berichtet. Wenn diese aber nach der Infektion akut neu aufgetreten, zunehmend oder mehr als nur milde ist, wenn sie den Alltag einschränkt oder mit weiteren Symptomen einhergeht, erfolgt die differenzialdiagnostische Abklärung

##### Husten (s. auch 10.5)

- Husten nach akuter Erkrankung findet sich häufig, z. B. noch in 17 % nach 3 Monaten [108]. Bei persistierendem Husten ist leitliniengemäß eine pneumologische Abklärung und Abgrenzung zu nicht-pneumologischen Hustenursachen zu empfehlen bzw. die weiterführende Diagnostik wie bei jedem anderen Husten.

##### Fieber

- Sekundäre bakterielle, virale oder fungale Infektionen v. a. nach SARS-CoV-2-assoziierter Pneumonie
- Rezidivierende Infektionen: Nach einer schweren Infektion kommt es häufig zu einer länger persistierenden Immunsuppression (wie beispielsweise nach Masern) und damit zu einer vermehrten Anfälligkeit für Infektionen. Das Ausmaß, die Qualität und die Dauer einer Immunsuppression nach COVID-19 und potenzielle Risikofaktoren dafür sind noch nicht ausreichend untersucht [109, 110].

##### Thorakale Schmerzen (s. auch 10.6)

- Thorakale Beschwerden treten häufig bei Patient:innen noch Wochen nach akuter Infektion auf. Die Ätiologie ist unklar, möglicherweise Folge der suspizierten autonomen Dysfunktion und Muskelschwäche im Rahmen des postviralen Zustandsbildes.
  Beispielsweise gibt es bei physiotherapeutischen Untersuchungen Hinweise für eine Einschränkung der Zwerchfellmobilität sowie Hinweise auf eine Muskelschwäche der Atemmuskulatur [111]. Letztere kann trotz normaler Lungenfunktion in Ruhe gegeben sein [112].

#### 8.1.2 Krankheitsbilder in möglicher Assoziation mit COVID-19

##### Residuale Pneumonie

In den ersten 3 Monaten nach COVID-19-Beginn bessern sich bei zwei Drittel der Patient:innen mit Viruspneumonie die CT-Auffälligkeiten deutlich, und es zeigen sich nur geringe Residuen (Milchglas und Retikulationen) [108]. Eine fehlende Besserung bzw. Zeichen von akuten oder chronischen Infektionen bedürfen einer spezifischen Abklärung. Hierbei sollte beispielsweise das Vorliegen einer organisierenden Pneumonie abgeklärt werden [113, 114]. Mittlerweile gibt es erste Metaanalysen und Übersichtsarbeiten zum 12-Monats-Verlauf nach Viruspneumonie: Zirka ein Drittel aller Hospitalisierten zeigt 12 Monate nach COVID-19 im CT noch Veränderungen [115]. Risikofaktoren für CT-Veränderungen inkludieren männliches Geschlecht, Alter >60 Jahren und intensivpflichtigen Verlauf [116]. Hierbei zeigen sich erfreulicherweise keine progredienten Fibrosen. Zum radiologischen Muster gehören Retikulationen, bandartige Strukturen, Milchglasveränderungen, selten Traktionsbronchiektasen und Wabenläsionen. Die Ausdehnung der Läsionen ist meist gering bis mittelschwer und betrifft häufig <25 % des Lungenparenchyms. Weiterhin ist die Relevanz einer pulmonalvaskulären Beteiligung beim Post-COVID-19-Syndrom unklar. Nach milder COVID-19-Erkrankung gibt es bisher kein Signal für eine pulmonale Hypertonie [115].

Erfreulicherweise bessert sich die Dyspnoe bei hospitalisierten Patient:innen im Verlauf deutlich. Die 24-Monats-Daten aus Wuhan zeigen nur mehr bei 14 % Kurzatmigkeit (mMRC ≥1, s. Abb. 11). Interessanterweise zeigte sich kein Unterschied zur Prävalenz in einer Gruppe passender Nicht-COVID-19-Erkrankter [117]. Die führende Veränderung in der Lungenfunktion war eine reduzierte Diffusion, vor allem in der Gruppe der intensivpflichtigen Patient:innen.

##### Pulmonalembolie

Trotz hoher Embolierate bei kritischem Verlauf auf der Intensivstation zeigen Nachsorgestudien klinisch eine geringe Inzidenz für Pulmonalembolien [2, 108]. Allerdings wurde das Vorliegen von Embolien oder Mikroembolien hierbei nicht systematisch untersucht. Die Frequenz, klinische Bedeutung und therapeutische Konsequenz von möglicherweise noch bestehenden Mikroembolien (bzw. „microvascular injury“) ist noch nicht geklärt [118].

- Bei akuter Dyspnoe mit D‑Dimer-Erhöhung oder anhaltender Dyspnoe mit Belastungsdesaturation oder Zeichen einer pulmonalen Hypertonie oder nur geringen strukturellen Veränderungen (unverhältnismäßig zur Dyspnoe) ist ein Angio-CT indiziert.
- Ein regelmäßiges Screening auf Mikroembolien ist in der Routine nicht empfohlen.

##### Lungenfibrosen

Ob und wie oft es nach Überleben eines COVID-ARDS oder nach einer schweren Viruspneumonie zu einer progressiven Fibrosierung der Lunge im Langzeitverlauf kommt, ist noch nicht abschließend geklärt [119]. Laut bisherigen Langzeitdaten ist dies eher ein seltenes Ereignis. Bei Befunden, die für einen progressiven interstitiellen Prozess sprechen, sollte gemäß etablierter Routine eine weitere Abklärung mittels Bronchoskopie mit (bronchoalveolärer Lavage [BAL] und Biopsie) folgen – entsprechend den Empfehlungen zur Diagnostik von interstitiellen Lungenerkrankungen.

##### Atemmuskelschwäche

Die muskuloskeletale Beteiligung bei Long COVID hat einen Gewichtsverlust durch Muskelverlust zur Folge. Damit verbunden kann eine Atemmuskelschwäche als Grundlage der Dyspnoe vorliegen (PI_max_ <80 mbar bei Männern, <70 mbar bei Frauen). Die Bedeutung und Spezifität einer Schwäche der inspiratorischen Atemmuskulatur in Abgrenzung zu Normalkontrollen ist allerdings weiterhin unklar [112, 120].

##### Veränderung der kleinen Atemwege

Eine SARS-CoV-2-Infektion kann, so wie andere Infekte, eine vorübergehende bronchiale Hyperreagibilität auslösen, der Stellenwert einer persistierenden Erkrankung der kleinen Atemwege („small airway disease“) ist allerdings unklar. Radiologisch konnte in ersten Arbeiten ein „air trapping“ nach mildem Verlauf häufiger als in Kontrollen nachgewiesen werden [121].

##### Dysfunktionale Atmung

Personen mit Post-COVID-Syndrom zeigen oftmals eine anhaltende Dyspnoe, insbesondere bei Belastung, ohne Hinweis auf strukturelle Veränderungen im Bereich der Lunge bzw. relevante Einschränkungen der Lungenfunktion. Dabei kann bei bis zu einem Drittel der Patient:innen anhand einer Spiroergometrie (Belastungsuntersuchung mit Erfassung der Atemgase) ein abnormes Atemmuster bei Belastung mit typischerweise steilem Anstieg des Atemminutenvolumens und fluktuierenden Werten für Atemäquivalente und Atemzugvolumen aufgezeigt werden [122–124]. Man bezeichnet diese stark schwankenden Veränderungen der Atemtiefe und -frequenz bei Belastung auch als Belastungshyperventilation. Ein weiterer Schirmbegriff hierfür ist dysfunktionale Atmung („dysfunctional breathing patterns“). Die Ursache hierfür ist noch nicht verstanden (Differentialdiagnose Störung der Atemregulation? Abnorme periphere Sauerstoffextraktion?).

##### Schlafassoziierte Störungen

Schlafstörungen sollten v. a. bei Fatigue abgefragt werden, da Schlafhygiene die Fatigue verbessern kann. Bei Durchschlafstörungen kann ein Schlafscreening oder eine Polysomnographie erfolgen, um diese zuzuordnen [125].

#### 8.1.3 Methoden der pneumologischen Abklärung

- In Ruhe (Spirometrie, Bodyplethysmographie, Diffusionskapazität, Blutgasanalyse, maximale inspiratorische Atemmuskelkraftmessung [MIP oder PI_max_]) und
- unter Belastung (z. B. 1‑Minute-Sit-to-Stand-Test, 6‑Minuten-Gehtest, Spiro‑/Ergometrie)
- Unter Berücksichtigung möglicher Vorerkrankungen sollte bei
  - pathologischer Lungenfunktion (FVC, TLC) oder
  - pathologischem Blutgasbefund (S_p_O_2_ in Ruhe oder Belastung) oder
  - einer verminderten CO-Diffusionskapazität (DLCO)
  eine Bildgebung mittels HRCT durchgeführt werden.

Bisherige Studien zeigen, dass eine eingeschränkte Diffusionskapazität (DLCO) in der COVID-19-Nachsorge von hospitalisierten Patienten in ca. 25 % diagnostiziert wird [2, 108].

Anmerkung zur Bildgebung: Das häufigste bildgebende Korrelat im HRCT nach einer Viruspneumonie sind Milchglastrübungen und Konsolidierungen, gefolgt von linearen Verdichtungen, sowie in Einzelfällen Traktionsbronchiektasen und lokalisierte fibrotische Zeichen [2, 108, 126].

### 8.2 Kardiologie

#### 8.2.1 Allgemeines

Eine kardiale Beteiligung bei anhaltenden Beschwerden nach COVID-19 ist nicht selten. In einer großen Case-Control-Studie an mehr als 150.000 Personen nach COVID-19-Infektion und mehr als 5 Mio. Kontrollpersonen mit einem Follow-up von etwa einem Jahr fand man nach 12 Monaten ein etwa 1,5- bis 2fach erhöhtes Risiko für Schlaganfall, Herzinfarkt, Arrhythmien und Herzinsuffizienz und ein etwa 5fach erhöhtes Risiko für Myokarditis. Bei Patient:innen, die wegen COVID-19 hospitalisiert wurden, war das Risiko etwa um das 3fache erhöht, bei Patient:innen nach Intensivstationbehandlung war das Risiko für diese Ereignisse um zumindest das 10fache erhöht [60].

Auch bildgebende Untersuchungen zeigen häufig kardiale Veränderungen im Langzeitverlauf nach COVID-19-Infektion: In einer Studie an 201 Personen mittleren Alters, die COVID-19 durchgemacht hatten (meist ohne Hospitalisierung) und persistierende Symptome aufwiesen, wurde etwa 4,5 Monate nach der Erkrankung eine Multiorgan-MR-Untersuchung durchgeführt. Bei 26 % der Patient:innen zeigten sich (meist milde) myokardiale Veränderungen: Myokarditis in 19 %, systolische Dysfunktion in 9 % [127]. Die klinische Bedeutung der Veränderungen ist nicht immer klar: In einer Case-Control-Studie in Hamburg wurden 443 Personen nach SARS-CoV-2-Infektion, die keiner Hospitalisierung bedurften, mit 1328 Kontrollpersonen verglichen. Es fanden sich geringgradige statistisch signifikante Verschlechterungen der Links- und Rechtsventrikelfunktion bei den Patient:innen post COVID-19, wobei die Werte im Normbereich lagen. Die Veränderungen im „cardiac MRI“ waren nicht unterschiedlich in beiden Gruppen, die Mittelwerte von Troponin und NT-proBNP waren post COVID-19 etwas höher, aber im Normbereich. Allerdings waren bei 33,2 % der Betroffenen post COVID-19 erhöhte (> 125 ng/l) NT-proBNP-Werte nachweisbar vs. 18,2 % der Kontrollgruppe. Interessanterweise waren Lebensqualität und psychosoziale Outcomes in beiden Gruppen gleich [128].

#### 8.2.2 Kardiologische Symptome im Zusammenhang mit COVID-19

Besonders bei kardialen Vorerkrankungen sind Verschlechterungen nicht selten. Die Folgen dieser akuten kardialen Manifestationen können auch bei Long COVID eine Rolle spielen. Eine Aufzählung dieser Akutereignisse findet sich weiter unten [129].

Des Weiteren wurde gezeigt, dass kardiovaskuläre Komplikationen innerhalb der ersten 6 Monate nach einer COVID-19-Erkrankung deutlich vermehrt auftreten. Dabei scheint die Inzidenz dieser direkt mit dem Schweregrad der vorangegangenen Erkrankung assoziiert zu sein. Patient:innen, die während ihrer akuten Erkrankung hospitalisiert waren, haben ein doppelt so hohes Risiko, im weiteren Verlauf auch eine kardiale Komplikation zu entwickeln, wie nicht Hospitalisierte. Hierbei ist insbesondere an venöse Thrombosen, ischämische Schlaganfälle, Myokardinfarkte, Lungenembolien und auch das Auftreten einer Herzinsuffizienz zu denken [130].

Mittlerweile gibt es von den großen kardiologischen Fachgesellschaften erste Empfehlungen im Sinne eines Expertenkonsensus zum Vorgehen bei kardiologischen Problemen im Rahmen von Long COVID [131]. Auch in Ermangelung einer wirklich ausreichenden Datenlage wird ein systematischer Zugang empfohlen. Die folgenden Ausführungen sind an die Empfehlungen des American College of Cardiology [131] 2022 angelehnt. Man bezieht sich hier auf Symptome und Erkrankungen, die eben nach COVID-19 auftreten UND NICHT DURCH EINE ALTERNATIVE DIAGNOSE ERKLÄRT WERDEN KÖNNEN. Die Symptome können nach allen Schweregraden der initialen SARS-CoV-2-Infektion auftreten, auch nach asymptomatischen Infektionen. Es wird auch empfohlen, zwischen kardiovaskulären Erkrankungen (klassischen Herz-Kreislauf-Erkrankungen) und kardiovaskulären Symptomen, die durch weiterführende Untersuchungen nicht wirklich erklärbar sind (oder nicht zur Gänze) zu unterscheiden. Diese Unterscheidung kann letztlich auch vom Ausmaß der durchgeführten Untersuchungen abhängen, wobei zu bedenken ist, dass nicht jede Veränderung z. B. in der Bildgebung auch mit Symptomen einhergeht bzw. korreliert. Letztlich ist auch bei Auffälligkeiten in der Bildgebung (z. B. MRI, FDG-PET) mangels Voruntersuchung häufig nicht klar, ob die Veränderungen vorbestehend sind oder durch die COVID-19-Infektion verursacht wurden.

##### Dyspnoe (s. auch 11.4 und 12.3.1)

In einer Long-COVID-Population wurde Dyspnoe von 43,4 % aller Patient:innen angegeben [24]. Die Dyspnoe ist sehr unspezifisch, jedoch eines der häufigsten Symptome in der Kardiologie (Herzinsuffizienz, Anginaäquivalent, Arrhythmie). Wenn eine Herzinsuffizienz zugrunde liegt, wird anhand der NYHA-Klassifizierung in das NYHA-Stadium I–IV eingeteilt [24]. Aufgrund des 2fach erhöhten Risikos für venöse Thromboembolien im ersten Jahr nach COVID-19 sollte neben kardialen Ursachen für Dyspnoe auch an eine Lungenembolie gedacht werden [98].

Interessanterweise findet man auch bei Fehlen einer spezifischen Diagnose häufig Abnormalitäten in der Spiroergometrie, wie z. B. niedrigere O_2_-Aufnahme, niedrigere anaerobe Schwelle, niedrigeres Schlagvolumen, höhere Herzfrequenzen sowie stärkere ventilatorische Ineffizienz [132].

##### Thorakale Schmerzen (s. auch 11.6)

Zirka 21,7 % der Patienten nach COVID-19 präsentieren sich mit thorakaler Schmerzsymptomatik [129]. Die Genese der thorakalen Schmerzen ist vielfältig, möglich als Erklärung für echte Angina pectoris ist eine persistierende endotheliale Dysfunktion (mikrovaskuläre Angina pectoris) oder auch eine vorher unentdeckt gebliebene makrovaskuläre KHK.

##### Palpitationen

Die Häufigkeit des Auftretens von Palpitationen bei Long COVID wurde mit 20 % angegeben [133]. Zur Abklärung dieser Beschwerden empfehlen sich wie sonst auch die Durchführung eines 12-Kanal-EKG, eines Holter-EKG sowie eine Ergometrie.

##### Orthostatische Intoleranz, inadäquate Tachykardie, POTS

(s. auch Autonome Dysfunktion, 8.10, 11.9 und 12.3.3)

#### 8.2.3 Weitere kardiale Krankheitsbilder im Zusammenhang mit COVID-19

Mögliche kardiale Begleiterscheinungen der akuten COVID-19 Erkrankung, deren Auswirkungen auch bei Long COVID eine Rolle spielen können, sind umfangreich und umfassen u. a. [129]:

- akute Perikarditis,
- Beschwerden ohne spezifische Ätiologie wie Palpitationen, Kreislauflabilität (s. auch 11.8 und 11.9),
- akute Herzinsuffizienz bis zum Lungenödem,
- akutes Koronarsyndrom (NSTEMI, STEMI),
- akute Stresskardiomyopathie,
- akute Myokarditis,
- supraventrikuläre und ventrikuläre Arrhythmien (am häufigsten Vorhofflimmern),
- akute rechtsventrikuläre Dysfunktion (nicht nur bei Lungenembolie).

Im Rahmen von Long COVID können folgende Herzerkrankungen auftreten [131]:

- Myokarditis,
- Perikarditis,
- Myokardischämie (neu oder verschlimmert) auf Basis einer (klassischen epikardialen) KHK oder einer mikrovaskulären Dysfunktion,
- nichtischämische Kardiomyopathie (rechts- oder linksventrikulär),
- Lungenembolien mit entsprechenden akuten und chronischen Folgen,
- Arrhythmien (z. B. Vorhofflimmern, Extrasystolen) Methoden der kardiologischen Abklärung,
- hypertensive Entgleisung.

Die Abklärung und Behandlung folgen den bestehenden Empfehlungen für die jeweiligen Krankheiten, wie oben ausgeführt, die Kausalität der durchgemachten SARS-CoV-2-Infektion ist häufig unklar bzw. schwer zu beweisen.

#### 8.2.4 Kardiologische Untersuchungsmethoden im Zusammenhang mit COVID-19

Die kardiologische Abklärung richtet sich nach der Symptomatik und dem Schweregrad. Sie kann je nach Situation folgende Untersuchungen umfassen [131]:

- BB, Troponin, NT-proBNP, CRP,
- EKG,
- Echo,
- 24-h-EKG,
- Thoraxröntgen (oder CT),
- Lungenfunktionsuntersuchung.

Bei pathologischen Ergebnissen, bekannten Herzerkrankungen mit neuen oder verschlimmerten Symptomen, dokumentierten kardialen Komplikationen während der akuten COVID-19-Infektion oder bei persistierenden unerklärbaren Symptomen sollte eine weiterführende kardiologische Untersuchung erfolgen.

**Figure 2:**
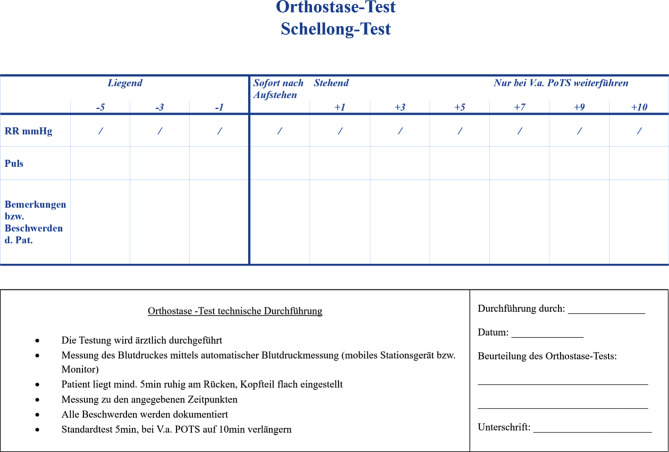

Weitere Details zu kardiologischen Untersuchungsmethoden, die bei Long COVID zum Einsatz kommen können:

- Die physikalische Untersuchung dient der Erkennung von Zeichen einer hydropischen Dekompensation und/oder einer Arrhythmie und umfasst unbedingt auch die Blutdruckmessung.
- Mittels 12-Kanal-EKG werden Frequenz und Rhythmus sowie allfällige Rhythmusstörungen erfasst. Unspezifische Veränderungen können bereits auf eine Myoperikarditis, Herzinsuffizienz oder eine KHK hinweisen. Das 24-h-EKG dient dem Nachweis von Arrhythmien und generell dem Verlauf der Herzfrequenz.
- Eine Laboruntersuchung zum Ausschluss anderer internistischer Ursachen für Dyspnoe soll bei (klinischen oder anamnestischen) Hinweisen auf Herzinsuffizienz bereits die Bestimmung eines NT-proBNP inkludieren. Ein NT-proBNP-Wert <125 pg/ml schließt das Vorhandensein einer symptomatischen Herzinsuffizienz weitgehend aus [134].
- Die Echokardiographie ist beweisend für die Diagnostik von verschiedenen Formen der Herzinsuffizienz (HFpEF bis HFrEF), wegweisend für die Erfassung einer pulmonalarteriellen Hypertension und liefert Hinweise auf eine KHK (z. B. Narben nach abgelaufenem Herzinfarkt).
- Belastungsergometrie – diese hat aufgrund der geringen Sensitivität im diagnostischen Algorithmus zur Abklärung einer koronaren Herzkrankheit mittlerweile einen geringeren Stellenwert. Sie wird aufgrund der guten Verfügbarkeit als Vorfelddiagnostik aber immer noch häufig eingesetzt. Sollte sich der Verdacht auf das Vorliegen einer koronaren Herzerkrankung erhärten, kommen je nach Höhe der Vortestwahrscheinlichkeit weitere nichtinvasive Untersuchungsmethoden (Myokardszintigraphie, Stressechokardiographie, Koronar-CT) oder die Koronarangiographie zur Anwendung. Im Rahmen eines postviralen Zustandes ist die Objektivierung einer Leistungseinschränkung ein Vorteil der Ergometrie, besser noch der Spiroergometrie.
- Der 6‑min-Gehtest mit Monitoring von Herzfrequenz, Blutdruck und Pulsoxymetrie kann ebenfalls rasch Aufschluss über die körperliche Leistungsfähigkeit geben.
- Der nichtinvasive Goldstandard für die Diagnose einer Myokarditis ist die Kernspintomographie, die in kleinen Fallserien nicht selten Myokarditis-typische Veränderungen nach COVID-19 zeigte [129].
- Aktiver Standtest (Schellong-Test): Unter sorgfältiger Observanz werden Herzfrequenz und Blutdruck nach dem Ruhen im Liegen und dann unmittelbar nach dem Aufstehen sowie nach 2, 5 und 10 min gemessen (Abb. 2).
- Kipptischuntersuchung bei orthostatischen Beschwerden oder Schellong-Test, wenn Klärung anders nicht möglich.

### 8.3 Neurologie

#### 8.3.1 Allgemeines

SARS-CoV‑2 konnte in unterschiedlichen Strukturen des Gehirnes nachgewiesen werden [135]. Die Viren verursachen nur sehr selten eine Enzephalitis [136]. Die Bedeutung dieser Befunde – insbesondere für Langzeitfolgen – ist aktuell sowohl für die Struktur als auch die Funktion des Gehirns unklar. Es ist darauf hinzuweisen, dass diese Patient:innen akut schwer erkrankt und an der SARS-CoV-2-Infektion verstorben waren. Hier bedarf es weiterer grundlagenwissenschaftlicher und klinischer Studien. In einer italienischen Studie konnte in 2 von 33 Autopsien Virus-RNA histologisch nachgewiesen werden, sodass insgesamt die Wahrscheinlichkeit eines direkten Virusbefalls des Gehirns als gering einzuschätzen ist, vor allem in Anbetracht der Schwere der untersuchten Fälle [137]. Ferner zeigten Studien, dass der RNA-Nachweis im Liquor (CSF) nur in einzelnen Fällen gelang und auch im Verlauf keine spezifische Antikörperproduktion nachgewiesen werden konnte [138] und nur in Ausnahmefällen (2/150 Lumbalpunktionen) oligoklonale Banden detektiert werden konnten [139]. Somit ist von einer persistierenden Infektion des ZNS NICHT auszugehen. Eine rezente Studie zeigt, dass inflammatorische Komponenten im Gehirn indirekte Ursache einer SARS-CoV-2-Infektion sind [106] und es sich nicht um ein neurotropes Virus handelt [105].

Bei einer prospektiven Dokumentation von hospitalisierungspflichtigen COVID-19-Patient:innen aus New York wird berichtet, dass nur 13 % eine neue neurologische Erkrankung zeigten, die auch vom Facharzt für Neurologie bestätigt wurde. Am häufigsten war hier die Enzephalopathie als Folge der systemischen Entzündungsreaktion bei der SARS-CoV-2-Infektion, die dem bekannten Krankheitsbild septischer Enzephalopathie entspricht [140].

Als Folgen einer SARS-CoV-2-Infektion, aber auch der Pandemie selbst [141] im Bereich von Neurologie und Psychiatrie berichtete **fast jede zweite Patient:in** von Müdigkeit, Muskelschmerzen, Biorhythmusstörungen, Angst oder Depression. Hier ist darauf hinzuweisen, dass es bezüglich der klinischen Symptomatik eine große Überlappung zu internistischen Fachgebieten (Rheumatologie, Kardiologie, Pulmologie) gibt. Es zeigte sich in einigen Studien eine positive Korrelation von Schweregrad der COVID-Erkrankung zu den Folgezuständen [2].

#### 8.3.2 Neurologische/psychiatrische Leitsymptome von Long COVID

- Postinfektiöse Müdigkeit
- Hirnleistungsstörungen (Konzentrations‑, Gedächtnisstörung)
- Kopfschmerzen
- Schlafstörungen
- Extremitätenschmerz (myalgisch, neuropathisch)
- Sensibilitätsstörungen (u. a. Missempfindungen, Taubheit)

R. Deer et al. [142] haben mittels „deep phenotyping“ als häufigstes Symptom Erschöpfung, gefolgt von Schlafstörung, kognitiver Funktionsstörung, Gedächtnisstörung beschrieben. Seltener bestanden Myalgien, Anosmie, Palpitationen und Kopfschmerzen. Die DGN hat mit Januar 2021 eine lebende S1-Leitlinie zur Beschreibung von neurologischen Komplikationen von COVID-19 para- und postinfektiös veröffentlicht [143]. Parainfektiöse immunologische Erkrankungen des Nervensystems wie ADEM (akute demyelinisierende Enzephalomyelitis) und Guillain-Barré-Syndrom wurden beschrieben [144, 145]. Eine MRT-Studie aus London [146]. zeigte, dass es nach eine SARS-CoV-2-Infektion zu einer Reduktion der grauen Substanz, des orbitofrontalen Kortex und parahippocampalen Gyrus sowie allgemein im Hirnvolumen im Vergleich zu vor SARS-CoV-2-Infektion und im Vergleich zu Kontrollen kam. Da klinische Daten der Patienten nicht vorlagen [146], bleibt unklar, inwieweit diese Veränderungen mit klinischen Symptomen korrelieren. Somit fehlen im Moment grundlegende Daten, ob diese Volumenabnahme mit einer reduzierten Funktion (z. B. fehlender Input bei Anosmie) im Sinne der Neuroplastizität potenziell reversibel ist oder einer permanenten Veränderung entspricht [147].

Zur Beteiligung autonomer Symptome s. dazu die Unterkapitel 8.10 (Überblick), 11.9 (Differenzialdiagnostik), und 12.3.3 (Autonome Dysfunktion)

#### 8.3.3 Neurologische Krankheitsbilder im Rahmen von Long COVID

##### Störungen der Hirnleistung

Da SARS-CoV‑2 kein neurotropes Virus ist, strukturelle Gehirnläsionen die Ausnahme sind, sollten weitere Differenzialdiagnosen ins Auge gefasst werden [148]. Fieber und Allgemeinerkrankungen können zu einer beeinträchtigen Hirnfunktion in individuell unterschiedlichem Ausmaß führen. Konzentrationsschwäche, Antriebsminderung, reduzierte Merkfähigkeit, verminderte Aufmerksamkeit und Kopfschmerzen bis hin zum Delir sind typische Manifestationen der akuten Phase und treten häufig auch im Rahmen der Krankheitsverarbeitung auf. Wenn solche Beschwerden nach der Genesung persistieren und die Grunderkrankung COVID-19 war, wird heute in der Literatur häufig der Begriff „brain fog“ verwendet, obwohl diesbezüglich keine medizinische Definition vorliegt. In einer rezenten Studie an 135 Patient:innen mit COVID-19 konnte gezeigt werden, dass kognitive Defizite vor allem mit dem Schweregrad der Erkrankung korrelieren und damit vor allem bei Patienten mit Intensivaufenthalt gesehen werden, während Symptome von Vergesslichkeit, Konzentrationsschwierigkeiten sowie Aufmerksamkeitsstörungen durch konventionelle Tests nicht gut quantifizierbar sind und eher bei Patienten mit mildem Verlauf berichtet werden [149]. Hier gibt es auch eine große Überlappung mit Fatigue, Schlafstörungen sowie Angst und Depressionssymptomen. Die Bewertung der wissenschaftlichen Evidenz ist in diesem Bereich besonders schwierig, da kognitive Störungen und Gedächtnisstörungen nach SARS-CoV-2-Infektionen gehäuft auftreten.

Pathophysiologisch werden unterschiedliche Faktoren diskutiert (Inflammation, Autoimmunität, Viruspersistenz, Immundysregulation, psychische Belastungsfaktoren), wobei für keine der angeführten Hypothesen belastbare Daten vorliegen [7]. Eine Gruppe aus Freiburg hat sich mittels FDG-PET-Untersuchung des Gehirns mit dieser Frage auseinandergesetzt. Mit der [18F]Fluordesoxyglucose-Positronenemissionstomographie wurde in einem kleinen Kollektiv eine Verminderung des Glukosestoffwechsels im Gehirn nachgewiesen, die mit solchen kognitiven Defiziten assoziiert war [150].

Die Arbeitsgruppe publizierte bereits Ergebnisse eines Follow-up von 8 Patient:innen der Originalstudie. Im Verlauf kam es zu einer signifikanten Besserung der kognitiven Defizite, wenngleich einige Betroffene auch 6 Monate nach der Akuterkrankung noch kein Normalniveau erreicht hatten [151].

Die Symptomverbesserung ging mit einer weitgehenden Normalisierung des Hirnstoffwechsels einher. Diese Daten sind primär Hypothesen-generierend und können nicht für diagnostische Zwecke für einzelne Patient:innen herangezogen werden. Weitere Studien konnten ebenfalls Auffälligkeiten im Hirnstoffwechsel zeigen, wobei in einer Studie vor allem die „Angstzentren“ auffällige Befunde zeigten [152–154].

Neuropsychologisch zeigt sich, dass die subjektiv wahrgenommenen und objektiv getesteten Beschwerden nicht immer übereinstimmen, Stressfaktoren und Angst jedoch ein wesentlicher Faktor sein dürften [155, 156].

Studien konnten zeigen, dass im Akutstadium neurodegenerative Marker im Serum von hospitalisierten Patient:innen deutlich erhöht waren, diese sich aber im Verlauf wieder normalisierten und somit nicht auf eine chronische Einschränkung zurückzuführen, sondern eine Reaktion auf die Hyperinflammation im Rahmen der Erkrankung sind [157, 158]. Bei Persistenz länger als 3 Monate ist eine fachärztliche neurologische Untersuchung zu empfehlen.

Gesicherte Therapien für Hirnleistungsstörungen (v. a. Exekutivfunktionen – Sequenzierung, Handlungsplanung) bei Long COVID sind limitiert. Studien zur Behandlung von Hirnleistungsstörungen bei Long COVID und deren Auswirkungen auf den Alltag der Betroffenen kommen insbesondere aus dem Bereich Ergotherapie [159–161]. Von einer spontanen Besserung ist prinzipiell auszugehen. Prinzipiell ist wichtig, auf eine adäquate Schlafhygiene zu achten und beeinflussende Faktoren (Angst, Depression, körperliche Überlastung) entsprechend zu therapieren. Obwohl ein kognitives Training für einzelne Patient:innen sinnvoll erscheint, liegen auch hierzu noch keine belastbaren Daten vor.

##### 8.3.3.1 Schmerzsyndrome

**Kopfschmerz **(Differenzialdiagnostik siehe 11.3)

Kopfschmerzen sind ein häufiges Symptom einer akuten SARS-CoV-2-Infektion, sie treten bei fast der Hälfte der Patient:innen auf. In 6–45 % persistieren sie über die akute Phase hinaus [162]. Dies ist häufiger der Fall bei Frauen und wenn Kopfschmerz das erste Symptom der Akuterkrankung war. Bereits vor der Infektion bestehende primäre Kopfschmerzen dagegen scheinen nicht mit Post-COVID-Kopfschmerz assoziiert [163]. Die Prävalenz dieser Post-COVID-Kopfschmerzen nimmt über die Zeit ab. So findet eine Metaanalyse bei insgesamt *n* = 28.438 COVID-19-Überlebenden Kopfschmerzen bei 47,1 % in der Akutphase und noch bei 8,4 % nach 6 Monaten [164]. Dieser Kopfschmerz tritt typischerweise bilateral auf, ist von mäßiger bis starker Intensität und hat häufig Charakteristika eines Kopfschmerzes vom Spannungstyp oder auch eines Migränekopfschmerzes mit den Begleitsymptomen Lärm‑, Lichtempfindlichkeit, Übelkeit und Erbrechen. In 61 % bestehen diese Kopfschmerzen täglich als Dauerkopfschmerz und imponieren als neu aufgetretener täglicher Kopfschmerz („new daily persistent headache“ [NDPH]), aber auch eine Verschlechterung vorbestehender primärer Kopfschmerzen ist möglich [164, 165].

##### Myalgien **(s. auch 11.11 und 12.3.8)**

Muskelschmerzen treten bei SARS-CoV-2-Infektion oft im Akutstadium auf, können aber auch nachher über Monate persistieren. Die Pathogenese ist nicht geklärt, die Differenzialdiagnose ist umfangreich und wesentlich. Zur Klärung tragen vor allem die Anamnese und der klinische Status bei, apparative Zusatzuntersuchungen sind im Einzelfall sinnvoll. In einer spanischen Case-Control-Studie [166] von hospitalisierten COVID-19-Patient:innen zeigte sich 7 Monate nach Krankheitsbeginn, dass das Auftreten von Myalgie bei Hospitalisierung mit präexistenten muskuloskeletalen Beschwerden korrelierte. Des Weiteren war Myalgie bei Hospitalisierung ein Prädiktor für die längerfristige Persistenz von Muskelschmerzen. Hier ist vor allem auf eine rheumatologische Abklärung hinzuweisen.

##### Neuropathische Schmerzen

Neuropathische Schmerzen werden nur vereinzelt berichtet und sind insbesondere vom Muskelschmerz abzugrenzen. Berichte über eine Small-Fiber-Neuropathie im Verlauf von SARS-CoV-2-Infektionen wurden wiederholt veröffentlicht [167, 168].

##### Störungen des autonomen Nervensystems (ANS) (auch: autonome Dysfunktion [AD]):

Diesen Störungen ist ein eigenes Unterkapitel 8.10 gewidmet.

#### 8.3.4 Abgrenzung anderer Beschwerdebilder gegenüber Long COVID

- **Critical-illness-Neuromyopathie** – Prolongierte Intensivaufenthalte mit Multiorganversagen führen zu einer nutritiv-toxisch bedingten Involution von Skelettmuskulatur und peripheren Nerven. Dieses Zustandsbild ist seit Jahrzehnten bekannt und wird anamnestisch, klinisch und elektrophysiologisch diagnostiziert.
- Persistenz einer **septisch-toxisch-metabolischen Enzephalopathie** nach ICU – Vor allem bei subklinischen zerebralen Vorschäden (z. B. Altersveränderungen des Gehirns) kann eine schwere Infektion mit ICU-Behandlungsbedarf durch Ausschüttung von Entzündungsmediatoren, Toxinen und Neurotransmitter-Imbalance zu einer prolongierten Aufwachphase mit Delir und persistierenden kognitiven Einbußen führen. Dieses Zustandsbild ist seit Jahrzehnten bekannt und wird anamnestisch, klinisch und mithilfe anderer Zusatzuntersuchungen diagnostiziert.
- **Verschlechterung vorbestehender neurologischer Erkrankungen** – Alle Erkrankungen des zentralen oder peripheren Nervensystems sowie der Skelettmuskulatur können sich durch eine schwere Allgemeinerkrankung passager oder auch dauerhaft verschlechtern. Patient:innen erreichen nach der Genesung von der Allgemeinerkrankung nicht mehr den vorherigen funktionellen Status. Dieses Zustandsbild ist seit Jahrzehnten bekannt und wird anamnestisch und klinisch diagnostiziert.
- Klinische **Manifestation subklinischer Gehirnerkrankungen** durch COVID-19 (z. B. „mild cognitive impairment“) – Chronische und bis dato unerkannte und subklinische Vorschädigungen des Gehirns können durch eine akute Infektion funktionell dekompensieren und nach Ausheilung des Infektes sich klinisch „erstmanifestieren“. Dieses Zustandsbild bedarf einer fachärztlichen Abklärung.

#### 8.3.5 Methoden der neurologischen Abklärung (spezialisierter Bereich bei Indikation)

- Generell lässt sich sagen, dass sich die Symptome im Rahmen und nach SARS-CoV-2-Infektion hinsichtlich ihrer Diagnostik als auch hinsichtlich ihrer Therapie nicht von Symptomen, die unabhängig von SARS-CoV‑2 entstehen unterscheiden
- Fokussierter neurologischer Status (Motorik, Sensibilität, kognitive Funktion)
- Labor: zur gezielten (!) Differenzialdiagnostik entsprechend Anamnese und Klinik s. Kapitel 11 je nach Symptomen, z. B. zur Identifikation entzündlicher Erkrankungen oder ursächlicher Stoffwechselstörungen
- Zur Beurteilung der kognitiven Leistungsfähigkeit: MMSE („Minimental State Examination“) oder MoCA (Montreal Cognitive Assessments, s. dazu 11.7), ist aber für enzephalopathische Störungen nicht validiert.
- Weiterführende Untersuchungen z. B. MRT, EMG/ENG, autonome Testbatterie/Kipptisch, Geruchstests, neuropsychologische Untersuchung, Schlaflabor, Neuropsychosomatik, ergotherapeutische Abklärung (insbesondere im Hinblick auf die Voraussetzungen für Handlungs- und Partizipationsfähigkeit und -möglichkeit bzw. Alltagsbewältigung), ggf. psychiatrische Konsultation

Weitere Quellen: [2, 4, 149, 150, 166, 169–173]

### 8.4 Hals-Nasen-Ohren-Heilkunde

#### 8.4.1 Allgemeines

Nach COVID-19 zeigen Patient:innen mitunter signifikante Beeinträchtigungen von Geruchsinn, Atmung, Stimme und Schlucken, die in einer individualisierten Rehabilitation nach COVID-19-Berücksichtigung finden müssen und einer HNO-ärztlich/phoniatrischen Diagnostik und logopädischen Therapie bedürfen.

#### 8.4.2 Leitsymptome und Krankheitsbilder im HNO-Bereich in Zusammenhang mit COVID-19

##### Riech- und Schmeckstörungen (s. auch 10.3 und 12.4.4)

Riechstörungen stellen ein häufiges Symptom der Infektion mit SARS-CoV‑2 dar [174]; 60–80 % der Betroffenen der ersten Infektionswelle klagen über einen Verlust des Riech- und Schmeckvermögens, oft nur vorübergehend für wenige Tage bis Wochen, eine Persistenz ist jedoch auch über mehrere Monate möglich [175]. Spätere Virusvarianten zeigen eine geringere Häufigkeit für Riechstörungen. So berichtet Doty in einer neueren Untersuchung bei der Delta-Variante (Herbst 2021) von 44 % und bei der Omikron-Variante (ab Winter 2021) von 17 % der Patient:innen mit einer Riechstörung [176]. Die Riechstörung wird aufgrund des plötzlichen Auftretens meist von den Patient:innen deutlich wahrgenommen. Die direkte Assoziation zu COVID-19 im Unterschied zu vorbestehenden Einschränkungen sollte gesichert sein. Untersuchungen des Langzeitverlaufs COVID-19-bedingter Riechstörungen zeigen, dass auch ein Riechverlust bis zu 1 Jahr und darüber hinaus vorkommt. Folglich kommt es bei den Betroffenen zu einer deutlichen Einschränkung der Lebensqualität und dem Wunsch nach Therapie der Beschwerden [177].

Besonders beeinträchtigend ist die **Parosmie** (Fehlriechen, die veränderte Wahrnehmung von Gerüchen, die meist als unangenehm wahrgenommen werden). Diese Form der Riechstörung tritt bei vielen Betroffenen mehrere Wochen bis Monate nach initialem Verlust des Riechvermögens auf, nachdem bereits ein Teil des Riechvermögens zurückgekehrt ist. Studien weisen darauf hin, dass dies als Zeichen der Regeneration des Geruchssinns aufgefasst werden kann [178].

#### 8.4.3 Andere Ursachen von Riechstörungen

Ein vermindertes Riechvermögen kann prinzipiell auf 2 pathophysiologische Mechanismen zurückgeführt werden. Zum einen kommt es bei konduktiven Riechstörungen (z. B. bei Nasenpolypen) zu einer verminderten Zuleitung der Duftstoffe zur Riechschleimhaut im oberen Bereich der Nase.

Bei den sensorineuralen Riechstörungen liegt die Ursache entweder in einer Funktionsstörung der Riechsinneszellen oder in übergeordneten zentralnervösen Strukturen entlang der Riechbahn.

Die COVID-19-bedingte Riechstörung fällt in die zweite Gruppe, in die auch vor Beginn der COVID-19-Pandemie andere Viren (z. B. Influenza‑, Parainfluenza‑, Rhinoviren) unter dem Begriff der postinfektiösen bzw. postviralen Riechstörung zusammengefasst wurden [179].

Ebenfalls in die Gruppe der sensorineuralen Riechstörungen fallen die posttraumatischen Riechstörungen, der Riechverlust bei neurologischen oder neurodegenerativen Erkrankungen (z. B. Morbus Parkinson, Morbus Alzheimer, Insult), bei medikamentös-toxischen Einflüssen, bei Tumoren der vorderen Schädelbasis, nach Chemo- oder Strahlentherapie oder bei internistischen Erkrankungen (z. B. Leber‑, Nierenerkrankungen). Selten besteht eine kongenitale Anosmie (isoliert oder im Rahmen des Kallmann-Syndroms).

Sollte keine Ursache im Rahmen der HNO-Abklärung, neurologischen, internistischen und allgemeinmedizinischen Abklärung gefunden werden, liegt eine idiopathische Riechstörung vor (bis zu 15–20 % der Patient:innen von Spezialambulanzen). Hier wird die Durchführung einer MRT-Untersuchung des Schädels empfohlen [179].

Als wichtige Differenzialdiagnose jeder Riechstörung ist die Mischform einer sensorineuralen und konduktiven Riechstörung in Form der chronischen Rhinosinusitis mit und ohne Nasenpolypen in Betracht zu ziehen. Sollten anamnestisch Hinweise auf diese Erkrankung vorliegen (z. B. Nasenatmungsbehinderung, Druckgefühl im Gesicht, nasale Sekretion), wird eine HNO-ärztliche Abklärung empfohlen.

##### Stimm- und Schluckprobleme

**Oropharyngeale Dysphagien** können u. a. nach Langzeitintubation, -beatmung, Tracheostomie sowie Intensivpflege sowohl bei NON-COVID-19- als auch COVID-19-Patient:innen auftreten. Pathophysiologisch liegen möglicherweise eine Koordinationsstörung zwischen Atmung und Schlucken, Pharynxschwäche, ein inkompletter laryngealer Verschluss oder eine Critical-Illness-Polyneuropathie zugrunde. Ob eine COVID-19-spezifische, neurogene Dysphagieätiologie vorliegt, ist derzeit nicht zu differenzieren und Gegenstand von Untersuchungen. Bei COVID-19-Patient:innen zusätzlich beeinträchtigend ist potenziell die Bauchlagerung mit verminderter Zugangsmöglichkeit im Rahmen der oralen Hygiene und potenziell vermehrter bakterieller Kolonisation der Mundhöhle mit denkbar erhöhtem Aspirationsrisiko.

Die Früherkennung einer Dysphagie ist für ein adäquates Patient:innen-Management wesentlich.

Bei 27 % der Patient:innen mit milder bis moderater COVID-19 wurde eine **Dysphonie** beobachtet. Sie kann als Initialsymptom (19 %), nach Erkrankung – selbst bei ursprünglich nicht hospitalisierten Patient:innen – oder im Rahmen von Long COVID auftreten und zu verbalen Kommunikationsproblemen führen. Dysphonie nach COVID-19 kann einerseits den oben genannten unspezifischen und den fraglich COVID-19-spezifischen pathophysiologischen Mechanismen geschuldet sein (Intubationsschäden am Kehlkopf, Folgen der Langzeitintubation oder Störung der neurogenen Koordination). Überdies bestärkt eine fraglich höhere Expression von ACE 2 bei COVID-19 die Hypothese von gesteigerten entzündlichen Prozessen der Stimmlippen („Corditis-Ätiologie“), wobei geschlechtsspezifische Unterschiede diskutiert werden. Darüber hinaus wurden auch paradoxe Stimmlippenbewegungen beobachtet. Außerdem kann eine Atemstörung nach COVID-19 auch laryngeal bedingt sein.

Weitere Quellen: [180–190].

##### Hörstörungen

Das Auftreten eines COVID-19-bedingten Hörverlustes wurde in der Literatur anekdotisch berichtet [191]. Ein zeitlicher Zusammenhang sollte gegeben sein, der entweder am Höhepunkt der Erkrankung oder auch wenige Wochen nach der Infektion zu finden ist. Es kommt entweder zu einer Schädigung des Labyrinths (Hörschnecke und Bogengänge) durch die akute Infektion oder die nachfolgenden Immunreaktionen. Neben der Hörstörung ist auf begleitende Symptome einer Labyrinthitis wie Schwindel und Tinnitus sowie Nystagmus zu achten.

Andere Ursachen der Hörstörung sind mittels otoskopischer Untersuchung (Cerumen, Otitis externa, Otitis media) oder durch weitere Untersuchungen auszuschließen (z. B. akustisches Trauma, Schädel-Hirn-Trauma, Morbus Menière, Otosklerose, Presbyakusis, medikamentös-toxische Ursachen oder innere Erkrankungen wie Hypertonie, Diabetes mellitus).

Bei unklarem Befund sollte eine retrocochleäre Ursache der Hörstörung (z. B. Akustikusneurinom) durch ein MRT des Schädels ausgeschlossen werden.

#### 8.4.4 Methoden der Diagnostik im HNO-Bereich

##### Riechtests

Im Rahmen des Managements der Betroffenen hat sich gezeigt, dass die Durchführung von Riechtests einen positiven Effekt aufweist, da dies den Patient:innen vermittelt, dass die Beschwerden ernst genommen werden. Außerdem hat sich gezeigt, dass die subjektive Selbsteinschätzung des Riechvermögens oft nicht mit objektivierenden Testverfahren übereinstimmt [192].

Es sind verschiedene Screeningtests zur einfachen und schnellen Testung des Riechvermögens erhältlich und auch für die Selbsttestung geeignet [193]. Bei Notwendigkeit der ausführlichen Testung (z. B. für gutachterliche Fragestellungen) sollten aber idealerweise Tests mehrerer olfaktorischer Dimensionen (Geruchsschwelle, Diskrimination, Identifikation) durchgeführt werden [194, 195]. Nur so kann die individuelle Diagnose einer Anosmie (Verlust des Riechvermögens), Hyposmie (vermindertes Riechvermögen) oder Normosmie (normales Riechvermögen) gestellt werden.

Besonders bei anamnestischen Unklarheiten, ob eine wirkliche Schmeckstörung (auf süß, sauer, salzig, bitter, umami) oder eine Riechstörung vorliegt, hilft die Durchführung validierter Tests der olfaktorischen und/oder gustatorischen Sensitivität [196]. So klagen die meisten Patient:innen mit Riechstörungen über eine damit einhergehende Störung des Feingeschmacks beim Essen und Trinken (durch das Fehlen der retronasalen Geruchswahrnehmung).

**Figure 3:**
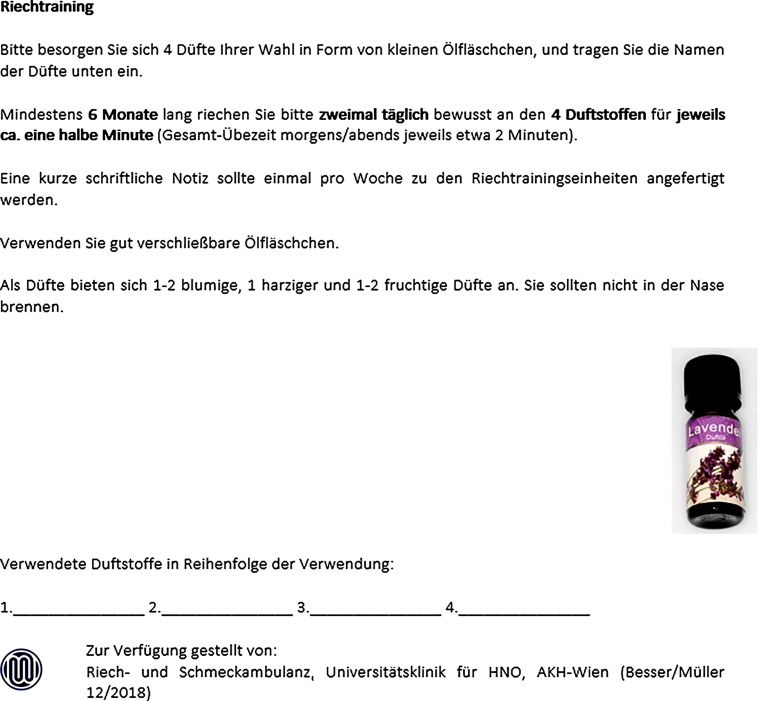

### 8.5 Dermatologie

#### 8.5.1 Allgemeines

Hautveränderungen können Begleitsymptome von der SARS-CoV-2-Infektion sein sowie bei vielen anderen Virusinfektionen auftreten. In der Literatur finden sich derzeit zahlreiche Hypothesen bezüglich der pathophysiologischen Mechanismen. Es gibt jedoch derzeit diesbezüglich keine sichere Evidenz. Weitere Studien sind daher diesbezüglich erforderlich.

Die Inzidenz von Hautmanifestationen wurde auch aufgrund methodischer Mängel der meisten Studien (Selektionsbias) überschätzt.

Es gibt in der Literatur zum Beispiel Berichte von COVID-19-assoziierten vesikulösen Exanthemen in denen schlussendlich verschiedene Herpesviren mittels PCR nachgewiesen werden konnten.

Der direkte Erregernachweis von SARS-CoV‑2 aus der Haut ist bisher nicht oder nur unzureichend gelungen, insbesondere bei den erwähnten Manifestationen.

COVID-19-assoziierte Hautmanifestationen können von sehr polymorpher Natur sein. Derzeit unterscheiden wir nachfolgende klinische Hautbilder, die länger als 4 Wochen anhalten können:

#### 8.5.2 Dermatologische Krankheitsbilder

##### Papulovesikulöses Exanthem

- Häufigkeit: 4–18 % der Hautveränderungen
- Zeitpunkt: Durchschnitt 3 Tage nach Symptombeginn
- Dauer: mediane Dauer 20 Tage, maximale Dauer 70 Tage
- Verteilung:
  1. Generalisiertes, polymorphes Muster
  2. Lokalisiertes Muster
- Symptomatik: kaum Pruritus
- Schweregrad der COVID-19-Infektion: moderat

##### Akrale Pernionen, sog. COVID-„Toes“

- Häufigkeit: ca. 28 % der Hautmanifestationen
- Zeitpunkt: asymptomatische PatientInnen
- Dauer: mediane Dauer 15 Tage, maximale Dauer länger als 130 Tage, Erkrankungsgruppe: Kinder und junge Erwachsene
- Verteilung: Füße, seltener Hände, Symptomatik: Schmerzen, Brennen, selten Juckreiz
- Schweregrad der COVID-19-Infektion: asymptomatische PatientInnen. In Zusammenhang mit den sog. COVID-Zehen wurde nachgewiesen, dass die meisten Patienten negativ im PCR-Test sind.

##### Livedo-reticularis/racemosa-Hautveränderungen

- Häufigkeit: weniger als 4 % der Hautmanifestationen
- Es gibt hierbei 2 Untertypen:
  1. Untertyp: Livedo-reticularis-Typ-Verteilung:
    Verteilung: symmetrisch, Symptomatik mild
    Schweregrad der COVID-19-Infektion: meist bei mildem Verlauf und nicht assoziiert mit thromboembolischen Ereignissen
  2. Untertyp: Livedo-racemosa-Typ-Verteilung:
    Verteilung: größere asymmetrische, anuläre Läsionen
    Schweregrad der COVID-19-Infektion: häufig assoziiert mit schwerer Koagulopathie

##### Purpura/vaskulitische Hautveränderungen

- Häufigkeit: 1–8 % der Hautmanifestationen
- Verteilung: Ausbreitung bis zu nekrotischen, ulzerierenden Läsionen; generalisiert oder lokalisiert in intertriginösen Arealen
- Erkrankungsgruppe: ältere Patient*innen
- Schweregrad der COVID-19-Infektion: schwere COVID-19-Verläufe
- Die Hautveränderungen sind mit der höchsten Mortalität assoziiert.

##### Immunvermittelte Hauterkrankungen

Durch Infektionen mit SARS-CoV‑2 können generalisierte, immunvermittelte Haut- oder Systemerkrankungen getriggert werden. Folgende dermatologische Erkrankungen im Rahmen von Long COVID sind derzeit in der Literatur beschrieben worden: Psoriasis vulgaris, systemischer Lupus erythematodes, Vaskulitis, Dermatomyositis und chronische rheumatologische Erkrankungen.

##### Parainfektiöse Phänomene

Wie bei jeder Infektionserkrankung können parainfektiöse Phänomene auch bei einer COVID-Infektion auftreten. Ein in der Literatur beschriebenes Beispiel ist Haarausfall.

##### Haarausfall

Auch Haarausfall wird als ein mögliches Long-COVID-Symptom in der Literatur beschrieben. Um einen kausalen Zusammenhang oder die Spezifität beurteilen zu können, bedarf es noch weiterer Daten. In einer chinesischen Kohorte trat Effluvium in 20 % der Patient*innen post COVID auf [197]. Eine spezifische Behandlung ist hier nicht notwendig – eine je nach Haarwuchszyklus (Telogen) zeitverzögerte jedoch vollständige Wiederherstellung sollte auch ohne die Gabe von Haarwuchs-stimulierenden Medikamenten erreicht werden. Eine andere reversible Ursache (u. a. Schilddrüse, Eisenmangel, Medikamente) sollte ausgeschlossen werden.

##### Seltenes

Hyperästhesien, toxisches Handekzem

#### 8.5.3 Methoden der dermatologischen Abklärung

Nachfolgende Methoden sollen zum Ausschluss von Differenzialdiagnosen helfen:

- Hautbiopsie inklusive einer direkten Immunfluoreszenz (nur bei Persistenz und unklarer Diagnose)
- Labor: Blutbild, Nierenfunktionsparameter, Elektrolyte, Leberfunktionsparameter, CRP, Gerinnung, bedarfsweise antinukleäre Antikörper + Subsets, ANCA, C3, C4, zirk. Immunkomplexe und Doppelstrang-DNA

#### 8.5.4 Differenzialdiagnosen

Virusexantheme aufgrund anderer Viren, Arzneimittelexantheme, Vaskulitis anderer Genese.

##### Zur Behandlung: Abschn. 12.3.9

Weitere Quellen: [198–204]

### 8.6 Psychiatrie

#### 8.6.1 Allgemeines

Psychiatrische Symptome und Krankheiten sind im Kontext von COVID-19 in mehrfacher Hinsicht von Relevanz:

- Das Bedrohungsszenario der COVID-19-Pandemie stellte eine psychische Belastung für die gesamte Bevölkerung dar und führte zu einer Zunahme von Symptomen von Angst, Depression und posttraumatischen Belastungsstörungen (PTSD).
- Patienten mit vorbestehenden psychischen Erkrankungen haben ein höheres Risiko, an COVID-19 zu erkranken und einen schwereren Verlauf zu entwickeln. Wenn bereits in der Akutphase multiple körperliche, aber insbesondere auch neurokognitive Symptome und Schlafstörungen auftreten, ergibt sich ein erhöhtes Risiko für persistierende Beschwerden post COVID [205].
- Schwerere Verläufe von COVID-19 können zu organischen psychischen Störungen wie Delirien führen.
- Psychische Reaktionen sind im Rahmen eines postviralen Zustands immer mitzudenken:
  - Psychische Reaktionen auf die veränderte Funktion
  - Psychische Alterationen in Zusammenhang mit pathophysiologischen Veränderungen
  - Grundsätzlich erscheinen Körper und Seele als eine lebendige Einheit
- Psychischer Stress wird als ein wichtiger Faktor in der Entstehung und Aufrechterhaltung von körperlichen und psychischen Beschwerden nach COVID-19 angesehen [206, 207].

#### 8.6.2 Psychiatrische Leitsymptome und Krankheitsbilder im Zusammenhang mit COVID-19

Die am häufigsten genannten psychiatrischen Symptome im Kontext eines Post-COVID-Zustands sind Angst, depressive Verstimmungen, PTSD, kognitive Störungen und Schlafstörungen. Darüber hinaus gibt es aber noch viele andere Störungen wie Zwangsstörungen, somatoforme Störungen, substanzbezogene Störungen, die gelegentlich auftreten oder sich gravierend verschlechtern können [208]. Die Häufigkeit psychischer Erkrankungen in einem Long-COVID-Sample wurde mit 39 % (nach 2 Monaten) angegeben [209].

##### Angst/Depression

Symptome von Angst und Depression werden ca. bei einem Viertel der Patient:innen mit Long COVID gefunden [210, 211], auch noch nach mehr als einem Jahr.

Es ist empfehlenswert, Patient:innen mit somatischen Beschwerden von Long COVID diesbezüglich zu befragen. Umgekehrt ist es sinnvoll, Personen, die sich primär mit psychischer Symptomatik präsentieren, nach dem Kontext mit COVID-19 zu fragen.

Die Kardinalsymptome der Depression sind gedrückte Stimmung, Freud- und Interesselosigkeit sowie Verminderung des Antriebs. Angst tritt vorwiegend als generalisierte Angststörung (anhaltende, diffuse Angst, verschiedenste Lebensbereiche betreffend) und/oder in Form von Panikattacken (kurz dauernde Anfälle heftiger Angst ohne Anlass) auf.

Meist werden diese Störungen als reaktiv betrachtet [212–214]. Es gibt aber auch Hinweise darauf, dass die mit COVID-19 verbundenen, inflammatorischen Prozesse lange anhalten können und das Risiko für Depressionen erhöhen können [215].

##### Posttraumatische Belastungsstörungen (PTBS)

Symptome von PTBS werden in der Akutphase der Pandemie sowohl bei Infizierten wie auch in der nicht infizierten Allgemeinbevölkerung häufig gefunden und wurden auch im Kontext von Long COVID beschrieben [209, 216]. Kardinalsymptome der PTBS sind die ständige Wiederkehr der traumatischen Situation in Gedanken, Vorstellungen und Träumen und damit verbunden ein Rückzug aus dem Alltag.

##### 8.6.2.1 Funktionelle Körperbeschwerden und somatische Belastungsstörung

Persistierende körperliche Symptome kommen in der Allgemeinbevölkerung häufig vor, und oft zeigt sich hier keine lineare Korrelation zwischen den Ergebnissen von Standardzusatzuntersuchungen und individueller Symptomausprägung [217].

Im ICD-11 wird auch deshalb die „Somatische Belastungsstörung“ eingeführt (im DSM‑V gibt es die Diagnosekategorie bereits), eine Diagnose, die körperliche Symptome unabhängig von einer messbaren somatischen Ursache als behandlungswürdig und behandelbar beschreibt, wenn sie bei dem Betroffenen mit dysfunktionalen Gedanken, Gefühlen und Verhaltensweisen assoziiert sind. Hier wird von einer Wechselbeziehung zwischen psychischer und somatischer Belastung ausgegangen [13]. So zeigt sich auch bei Long/Post COVID, dass persistierende körperliche Symptome mit Zeichen einer somatischen Belastungsstörung sowie mentalem Stress assoziiert sind und kaum mit den Ergebnissen von Standardzusatzuntersuchungen korrelieren [218]. Wissenschaftliche Untersuchungen versuchen schon länger biologische Testverfahren zur Differenzierung und Diagnostik persistierender Körperbeschwerden zu entwickeln, wenn Standardzusatzuntersuchungen keine klare Abgrenzung erlauben. Hier bietet sich bei Long/Post COVID eine Chance [77, 101, 105, 219–223], wie die Autoren Iwasaki und Putrino in einem Kommentar formulieren: „Trotz der multifaktoriellen Pathogenese zeigen die verfügbaren Daten, dass es sich bei Long-COVID um ein organisches postakutes Infektionssyndrom (PAIS) mit einer eindeutigen physiologischen Funktionsstörung handelt, die mit medizinischen Standarddiagnosetests oft nicht konsequent nachgewiesen werden kann. Diese Diskrepanz unterstreicht den Bedarf an einer neuen Generation empfindlicherer Testverfahren für Menschen mit PAIS.“ Möglich ist auch, dass sich so in weiterer Zukunft verschiedene Ursachen für solche persistierende Körperbeschwerden und auch differenzielle Behandlungen entwickeln lassen. Epigenetische [224] Veränderungen und differenzielle inflammatorische Signaturen [49] sind hier erste Ansätze bei Long/Post COVID.

Pathogenetisch spielt wahrscheinlich mentaler Stress eine wichtige Rolle und psychoneuroimmunologische Mechanismen verbinden die psychischen und körperlichen Symptome in bidirektionaler Weise [225, 226]. Wichtig ist hier zu betonen, dass es sich somit keineswegs um rein „psychogene“ Beschwerden handelt, sondern dass im Sinne des biopsychosozialen Modells biologische (z. B. Inflammation, Genetik, somatosensorische neuronale Verstärkermechanismen), psychische (z. B. Katastrophisieren, Angstvermeidungsstrategien, individuelle Krankheitswahrnehmung und Symptominterpretation [227]) und soziale (z. B. Lockdown, Krankheitsnarrative in der Gesellschaft) Faktoren in individuell unterschiedlichem Ausmaß an der Symptomentstehung beteiligt sind [228].

Für Patient:innen mit somatischer Belastungsstörung oder auch einzelnen funktionellen Körperbeschwerden (z. B. auch Fatigue) besteht nach der S3-Leitlinie „funktionelle Körperbeschwerden“ [229] ein wirksamer und evidenzbasierter Therapieansatz in der multimodalen Therapie (z. B. Medikation, Psychoedukation, Entspannungsverfahren, CBT, Physiotherapie, Ergotherapie, gesunde Ernährung usw.). Bis zum Nachweis spezifischerer Therapien z. B. für Subgruppen von Patient:innen sollte diese als Therapie der Wahl empfohlen werden.

**Erschöpfung** (siehe 11.1, 12.2 und 12.3.2)

**Kognitive Störungen** (siehe 11.7 und 12.3.7)

**Schlafstörungen** (siehe 11.10)

**Des Weiteren:** Zwangsstörungen, somatoforme Störungen, substanzbezogene Störungen [208]

##### Empfehlung

Patient:innen, die sich mit psychischen und/oder sozialen Symptomen und Belastungsfaktoren im Gefolge einer Infektion mit SARS-CoV‑2 vorstellen, sollten auch einer somatischen Differenzialdiagnostik zugeführt werden.

##### Empfehlung

Patient:innen, die sich mit somatischen Beschwerden im Rahmen postviraler Zustände von Long COVID vorstellen, sollten aktiv nach den genannten Symptomen von Angst, Depression, und psychosozialen Belastungen gefragt werden.*

* Die Co-Autorinnen Kathryn Hoffmann und Eva Untersmayr sind mit dieser Empfehlung in der vorliegenden Formulierung im Dissens.

##### Empfehlung

Patient:innen mit anhaltenden postviralen Symptomen nach COVID-19 sollten im Rahmen eines multimodalen Therapiekonzepts auch Angebote zu psychosozialer Betreuung erhalten.*

* Die Co-Autorinnen Kathryn Hoffmann und Eva Untersmayr sind mit dieser Empfehlung in der vorliegenden Formulierung im Dissens.

#### 8.6.3 Methoden der psychiatrischen Abklärung

Untersuchende stehen vor der Aufgabe, psychischen Beschwerden den richtigen Stellenwert zuzuordnen. Zum einen geht es darum, psychische Beschwerden im Kontext von Long COVID nicht zu übersehen, zum anderen aber auch darum, somatische Beschwerden ohne fassbaren Befund nicht als „psychisch“ abzustempeln.

Zum Screening nach Depression und Angst in der Praxis stehen zahlreiche kurze praktikable Fragebogen zur Verfügung, z. B. für Depression WHO‑5 [230], für Angststörungen GAD‑2 [231], für PTBS [232].

Derartige Fragebögen vermitteln keine Diagnose [233], bieten aber erste Anhaltspunkte für die weitere Exploration. Dabei muss man berücksichtigen, dass kürzere Fragebögen eine geringere Treffsicherheit haben als längere [234]. Basis der psychiatrischen Diagnostik ist noch immer das ärztliche Gespräch. Wichtig ist auch, ob es sich bei den psychischen Beschwerden um eine Erstmanifestation handelt oder ob es bereits eine längere Vorgeschichte gibt. Des Weiteren ist zu bedenken, dass psychische Beschwerden, die im Kontext von Long COVID auftreten, nicht zwangsläufig damit kausal zusammenhängen müssen, sondern auch andere Ursachen haben können.

#### 8.6.4 Differenzialdiagnosen

Differenzialdiagnostisch sind bei Depression und Angst die möglichen (sehr seltenen) organischen Ursachen wie endokrine Störungen und hirnorganische Veränderung zu bedenken. Auch an eine postvirale Fatigue ist zu denken, s. dazu auch Abschn. 8.9.

##### 8.6.4.1 Fatigue und kognitive Beeinträchtigungen

Psychoneuroimmunologische Modelle wie Immunhypothese der Depression werden jetzt wieder mit neuen Termini wie die postinfektiöse Müdigkeit, Chronic-Fatigue-Syndrom, ME diskutiert. Ihre Pathomechanismen sind jedoch noch nicht völlig geklärt [235]. Da es keine biologischen Marker gibt, beruhen die diagnostischen Kriterien auf Expertenkonsens. Allerdings wird die ICD-11-Klassifizierung diagnostische Leitlinien für die Diagnose „postviral fatigue syndrome“ (PVFS) enthalten. Kognitive Beeinträchtigungen sind Teil dieses Syndroms. Wie weit die Fatigue-Symptome nach COVID-19 zu dem meist chronisch verlaufenden CFS/ME [236] werden können, ist noch unklar [237]. Aus psychiatrischer Perspektive ist die mögliche Differenzialdiagnose die Depression, wobei die fehlende traurige Verstimmung der Patienten:innen mit PVFS einen Hinweis auf Überschneidungen bzw. Komorbiditäten geben kann.

### 8.7 Kinder

#### 8.7.1 Allgemeines

Im Gegensatz zu anderen Altersgruppen zeigen Kinder und Jugendliche zuallermeist einen milden oder asymptomatischen Krankheitsverlauf [238, 239]. Allerdings haben Kinder und Jugendliche massiv unter den Folgen der Mitigationsmaßnahmen und dem weiterhin fehlenden Infektionsschutz gelitten [240]. Besonders in dieser Altersgruppe können Symptome, welche nach einer SARS-CoV-2-Infektion beobachtet werden, in manchen Fällen nicht in direktem Zusammenhang mit der Infektion selbst stehen: Sie können Folge der psychischen Belastung durch Mitigationsmaßnahmen, weiterhin fehlendem Infektionsschutz in Kindergärten und Schulen, Folgen der Erkrankung/Tod von Bezugspersonen oder Freunden sowie von anhaltendem starkem Erfolgsdruck durch Schule und Gesellschaft trotz Pandemie, Klimakrise und Inflation sein, ebenso wie bei Kindern und Jugendlichen ohne SARS-CoV-2-Infektion [35].

Selten leiden jedoch auch Kinder gänzlich unabhängig von der Schwere des Krankheitsverlaufs unter anhaltenden Beschwerden nach durchgemachter SARS-CoV-2-Infektion [241, 242].

Prinzipiell zu unterscheiden gilt es zwischen Long COVID – ähnlich wie bei Erwachsenen – und dem Hyperinflammationssyndrom, welches auch MIS‑C („multisystem inflammatory syndrome in children“) oder PIMS („paediatric inflammatory multisystem syndrome“) genannt wird. Dieses tritt bei Kindern, Jugendlichen und jungen Erwachsenen unter 21 Jahren etwa 2 bis 8 Wochen nach einer SARS-CoV-2-Infektion auf. ist jedoch eine eigene Krankheitsentität und zählt daher streng genommen nicht zu Long COVID. Dieses Krankheitsbild soll dennoch Eingang in dieses Papier finden, um Awareness zu schaffen, da es sich bei diesem Symptomkomplex um ein unter Umständen lebensbedrohliches Syndrom handelt (s. unten).

#### 8.7.2 Long COVID

Long COVID bei Kindern bezieht sich auf die bei Erwachsenen geltenden Definitionen und beschriebenen, andauernden Symptome nach einer SARS-CoV-2-Infektion. Diese Beschwerden umfassen ähnlich wie im Erwachsenenalter unter anderem Müdigkeit, Kopf- und Gliederschmerzen, Störungen von Geruchs- und Geschmackssinn, Kurzatmigkeit, Konzentrationsstörungen, mangelnde körperliche Belastbarkeit [241].

Bei der präliminären Zwischenauswertung einer Analyse von 755 Kindern im Alter von 0 bis 14 Jahren mit SARS-CoV-2-Infektion (durchgeführt von der MedUni Graz, AGES und ÖGKJ) zeigt sich, dass 11 % Beschwerden nach 1 Monat und 6 % Beschwerden 3 Monate nach der SARS-CoV-2-Infektion angeben, die mit Long COVID vereinbar sind, wobei ältere Kinder (10 bis 14 Jahre) mit 16 % (Symptome nach >1 Monat) und 10,7 % (Symptome nach >3 Monaten) häufiger betroffen waren. Die in der Gesamtkohorte (alle Altersgruppen) am häufigsten angegebenen Symptome waren Müdigkeit (4,2 % nach 1 Monat bzw. 2,3 % nach 3 Monaten), Kopfschmerzen (2,6 % bzw. 1,7 %), Kurzatmigkeit (2,1 % bzw. 1,3 %), Konzentrationsstörungen (1,9 % bzw. 1,1 %), eingeschränkte körperliche Belastbarkeit (2,1 % bzw. 1,1 %), gefolgt von Husten, Halsschmerzen, Gelenk‑/Gliederschmerzen und Bauchschmerzen (nach 1 Monat jeweils 0,7–1,1 % bzw. nach 3 Monaten 0,4–0,7 %). Störungen von Geruchs- und Geschmackssinn wurden bei Kindern unter 10 Jahren seltener als 1 % angegeben, bei Kindern >10 Jahre jedoch in 5,6 % (nach 1 Monat) bzw. 3,3 % (nach 3 Monaten) berichtet. Diese Symptome wurden von den Familien als mit der Infektion im Zusammenhang stehend beurteilt. Eine Abgrenzung gegenüber Beschwerden anderer Ursache ist jedoch im Einzelfall schwierig, sodass die Rate der tatsächlichen anhaltenden Symptome nach COVID-19 unter den angegebenen Häufigkeiten liegen dürfte. Eine Arbeit mit Daten von 1734 SARS-CoV-2-positiven Kindern und Jugendlichen (5 bis 17 Jahre) aus dem Vereinigten Königreich zeigte nach COVID-19 anhaltende Symptome nach 1 Monat bei 4,4 % (77 von 1734) und nach 3 Monaten bei 1,8 % (25 von 1379). In dieser Arbeit waren höheres Alter der Kinder sowie ein schwererer Krankheitsverlauf mit anhaltenden Beschwerden positiv korreliert. Auch in dieser Arbeit erwiesen sich Müdigkeit und Kopfschmerzen als die am häufigsten angegebenen Symptome [243].

Die diagnostische Abklärung von Long COVID im Kindesalter sollte sich wie bei Erwachsenen an den beschriebenen Beschwerden und dem Ausschluss anderer Differenzialdiagnosen orientieren (s. dazu Kap. 8 und 9). Die diagnostische Abklärung kann sich dabei an den einzelnen Kapiteln dieser Leitlinie orientieren, wobei auf die pädiatrischen Gegebenheiten Rücksicht genommen werden muss. Neben der Abklärung anderer organischer Differenzialdiagnosen ist eine Berücksichtigung psychischer Ursachen von essenzieller Bedeutung.

Neben einer Basisblutabnahme (mit Blutbild und Chemie) sollten je nach Beschwerdebild EKG, Blutdruckmessungen, Lungenfunktion und ggf. 24-h-Blutdruck, Belastungs-EKG/Ergometrie und Herzultraschall in Betracht gezogen werden. Auch eine klinisch psychologische Evaluierung zu diagnostischen Zwecken oder auch zur Entlastung bei über viele Wochen bestehenden Symptomen sollte erwogen werden.

#### 8.7.3 MIS-C/PIMS-TS

Das klinische Erscheinungsbild des Multiinflammationssyndroms bei Kindern (MIS-C), auch als pädiatrisches inflammatorisches Multisystemsyndrom (PIMS-TS) bezeichnet, variiert und reicht von Fieber (mit oder ohne Bauchschmerzen) ohne andere erklärbare Ursache mit stark ausgelenkten Entzündungsparametern bis zu einem Kawasaki-Syndrom-ähnlichem Phänotyp oder einer Präsentation mit Schock, Gerinnungsstörung und Multiorganversagen. Prinzipiell handelt es sich um ein progressives Erscheinungsbild, das zumeist mild beginnt und innerhalb einiger Tage zu einer schweren Dysfunktion mehrerer Organsysteme führen kann [244, 245].

Ähnlich wie bei Kawasaki-Syndrom können sich bei einem Teil der Kinder Koronaraneurysmen bilden.

Abgesehen von der relativ späten Symptomentwicklung nach einer Infektion unterscheidet sich MIS‑C auch bezüglich der auffindbaren Biomarker und Zytokine von der Hyperinflammation bei COVID-19 im Erwachsenenalter [246].

Die WHO definiert MIS‑C wie folgt:

- Kinder und Jugendliche ≤19 Jahre mit Fieber ≥3 Tage
- UND 2 der folgenden Kriterien:
  - Exanthem oder bilaterale non-purulente Konjunktivitis oder mukokutane Entzündungszeichen (Mund, Hände, Füße)
  - Hypotension oder Schock
  - Zeichen einer myokardialen Dysfunktion, Perikarditis, Valvulitis oder Koronaranomalien
  - Zeichen einer Gerinnungsstörung (PT, PTT, erhöhtes D‑Dimer)
  - Akute gastrointestinale Beschwerden (Diarrhö, Erbrechen oder Abdominalgie)
- UND erhöhte Entzündungsparameter (Blutsenkung, C‑reaktives Protein oder Procalcitonin)
- UND keine andere offensichtliche Ursache der Entzündung wie bakterielle Sepsis, toxisches Schocksyndrom
- UND Evidenz für COVID-19 (positiver PCR-, Antigentest oder Serologie) oder wahrscheinlicher Kontakt zu SARS-CoV‑2

(Adaptiert nach WHO [247], Mai 2020)

Die Inzidenz ist derzeit ebenfalls nicht ausreichend geklärt. In einer Metaanalyse, im Zuge derer Daten aus 26 Ländern und insgesamt 7780 Kinder mit SARS-CoV-2-Infektion eingeschlossen wurden, wurde die Inzidenz auf 0,14 % aller SARS-CoV-2-Infektionen im Kindes- und Jugendalter geschätzt [248]. Probleme bei der Inzidenzabschätzung sind das Fehlen eines allgemein gültigen Meldesystems für MIS‑C, die vielfältige klinische Präsentation und dass die Zahl SARS-CoV-2-infizierter Kinder vermutlich höher ist als die tatsächlich gemeldeten Fälle. Von Februar 2020 bis April 2022 wurden in Österreich 214 Fälle eines MIS‑C gemeldet; 72 davon benötigten die Aufnahme auf einer Intensivstation. Ein Jugendlicher benötigte eine extrakorporale Membranoxygenierung (ECMO). Im selben Zeitraum sind 656.733 bestätigte SARS-CoV-2-Infektionen in dieser Altersgruppe registriert worden, was einer Inzidenz von 0,03 % aller SARS-CoV-2-Infektionen im Kindes- und Jugendalter entsprechen würde (ÖGKJ, AGES 2021), jedoch zeigen die Daten, dass die Inzidenz vor Auftreten der Delta-Variante höher war (ca. 1 MIS-C-Fall pro 1000 infizierter Kinder/Jugendlicher), als bei Infektionen mit der Delta- oder Omikron-Variante (ca. 1 MIS-C-Fall pro 4900 infizierter Kinder/Jugendlicher).

Der Pathomechanismus ist bisher unbekannt. Es dürfte sich jedoch um ein multifaktorielles, immunologisches Geschehen nach Kontakt des Körpers mit dem Virus handeln [249].

Die Überlappungen mit dem Kawasaki-Syndrom deuten auf eine Vaskulitis und ein autoimmunologisches Geschehen hin. Diskutiert werden unter anderem eine Kombination aus einer überschießenden T‑Zell-Antwort und dem Auftreten von Autoantikörpern nach einer SARS-CoV-2-Infektion [246].

In der Therapie kommen vornehmlich intravenöses Immunglobulin und hoch dosierte Glukokortikoide zum Einsatz. Ein hochfieberhaftes Zustandsbild bei Kindern und Jugendlichen wenige Wochen nach (möglicher) SARS-CoV-2-Infektion ohne eindeutige andere Ursache sollte an dieses Krankheitsbild denken lassen. Eine frühzeitige Kontaktaufnahme mit definierten Kompetenzzentren und ein interdisziplinäres und bedarfsorientiert auch interprofessionelles Management sind essenziell [250, 251].

##### Empfehlung

Bei hochfieberhaftem Zustandsbild bei Kindern und Jugendlichen wenige Wochen nach (möglicher) SARS-CoV-2-Infektion ohne eindeutige andere Ursache sollte an dieses Krankheitsbild gedacht werden. Eine frühzeitige Kontaktaufnahme mit definierten Kompetenzzentren und ein interdisziplinäres Management sind essenziell.

### 8.8 Immunologie: immunologische Dysfunktion, Immunopathien

#### 8.8.1 Allgemeines

Immunologische Mechanismen wie die zeitgerechte und adäquate Induktion einer virusspezifischen Immunantwort sind für den Schweregrad und Verlauf einer akuten COVID-Infektion entscheidend [252, 253]. Wissenschaftliche Studien legen jedoch ebenso immunologische Ursachen für weiterbestehende Symptome nach der akuten Erkrankung im Sinne eines postviralen Zustandes nach COVID-19 oder PASC nahe. Hierbei werden neben vorbestehenden immunologischen Veränderungen eine mukosale Dysbiose des Viroms, Mykobioms sowie des bakteriellen Mikrobioms, autoimmunologische Mechanismen oder eine Immundysregulation als Ursachen diskutiert [76].

#### 8.8.2 Anhaltende Symptome nach COVID-19 in Kombination mit rezidivierenden schweren Infekten

In einer prospektiv durchgeführten Studie an 123 COVID-Patienten konnte anhand der Symptome während der akuten Infektion sowie Alter, Komorbiditäten und einer spezifischen Antikörpersignatur das Risiko für die Entwicklung von PASC abgeschätzt werden [51]. Eine Häufung immunologischer Veränderungen bei Patient:innen mit postviraler Fatigue ist seit Längerem bekannt. Hier dürften unter anderem auch Veränderungen des Komplementsystems eine Rolle spielen. Eine bereits zu Beginn der SARS-CoV-2-Pandemie im Jahr 2020 durchgeführte prospektive Beobachtungsstudie zeigte bei einer Kohorte von 23 Personen, die an PASC erkrankt waren, und an 19 Personen, die an PASC erkrankt waren und den Diagnosekriterien von myalgischer Enzephalomyelitis/Chronic-Fatigue-Syndrom (ME/CFS) entsprachen, eine Reduktion des Mannose bindenden Lektins (MBL) bei 23 % bzw. 17 % der Patienten. Ebenso waren in der Studie bei 14 % bzw. 11 % der Patienten die C3-Werte vermindert [254]. Auch in einer bereits vor der SARS-CoV-2-Pandemie durchgeführten österreichischen Studie unter Einschluss von Patienten mit ME/CFS nach Epstein-Barr-Virus(EBV)-Infektion, zeigte sich eine diagnostizierte MBL-Defizienz bei 7 % der Patient:innen, was über den Werten liegt, die in der allgemeinen Bevölkerung in Europa zu finden sind [255].

##### Empfehlung

Basierend auf diesen Daten wird daher angeraten, bei von PASC Betroffenen, die zusätzlich wiederkehrende schwere Infekte haben, eine umfangreiche immunologische Abklärung im spezialisierten niedergelassenen Bereich oder an spezialisierten Zentren durchzuführen.

Rezidivierende Virämie und Persistenz von SARS-CoV‑2 wurde bei Patient:innen unter immunsuppressiver Kombinationstherapie beschrieben [256]. Ebenso konnten Virus-RNA oder Virusfragmente endoskopisch in Biopsien oder durch Probenentnahme im respiratorischen sowie gastrointestinalen Gewebe Monate nach einer Infektion nachgewiesen werden [257, 258]. Bei einer Gruppe von Patient:innen mit chronisch entzündlichen Darmerkrankungen konnte die Persistenz von Virusfragmenten im gastrointestinalen Gewebe vor allem bei solchen mit PASC-Symptomatik nachgewiesen werden [259]. Ebenso wurde bei einer Gruppe von PASC-Patient:innen mit Post-Exertional-Malaise (PEM) eine EBV-Reaktivierung festgestellt [74].

##### Empfehlung

Bei PASC-Patient:innen mit Immunsuppression oder Immundefizienz, Milz‑, Lymphknoten- oder Lebervergrößerung sollte eine Abklärung auf virale Erreger angeboten werden.

#### 8.8.3 Autoimmunologische Aspekte

SARS-CoV-2-Infektionen gehen mit der Induktion von Autoantikörpern gegen verschiedenste körpereigene Proteine einher. Diese Autoantikörper sind nicht nur gegen Bestandteile des Immunsystems gerichtet und werden daher mit einem schweren Verlauf der akuten Infektion in Verbindung gebracht [260, 261], die Entwicklung von Autoantikörpern wird auch als eine weitere mögliche Ursache für die Pathogenese von PASC diskutiert. Gerade die Induktion von agonistisch wirksamen Autoantikörpern gegen G‑Protein-gekoppelte Rezeptoren dürfte in der Pathogenese von PASC eine Rolle spielen [262]. Die Assoziation zwischen Autoantikörpern gegen G‑Protein-gekoppelte Rezeptoren (GPCR) und Fatigue-Symptomatik ist bekannt [263]. Bei PASC-Betroffenen sind nicht nur Autoantikörper gegen GPCR des autonomen Nervensystems mit vasoregulatorischer oder immunregulatorischer Wirkung vorhanden, diese korrelieren auch mit der Symptomschwere [264]. In diesem Zusammenhang wird auch auf den Zusammenhang zwischen orthostatischer Dysregulation und der Präsenz von Autoantikörpern gegen GPCR bei einer Subgruppe der Patient:innen hingewiesen, was eine autoimmune Genese der Beschwerden bei diesen Patient:innen nahelegen könnte [265].

#### 8.8.4 Zum Konzept der Mastzellüberaktivierung

Neben der diskutierten Persistenz von inflammatorischen Zytokinen sowie der chronischen Aktivierung von Monozyten und Lymphozyten [266, 267], spielt in der Klinik von PASC auch eine Dysregulation von Mastzellen eine Rolle. Durch den virusinduzierten Untergang von Epithelzellen kommt es während der akuten Infektion zu einer vermehrten Ausschüttung von Alarminen wie IL-25, IL-33 und TSLP, wodurch Typ-2-Immuneffektorzellen wie ILC2, Mastzellen und eosinophile Granulozyten aktiviert werden [268]. Nach schweren COVID-Verläufen sind bis zu 9 Monate nach Infektion erhöhte Spiegel unter anderem von Th2-Zytokinen im Blut der Patient:innen nachweisbar [266]. PASC-Patient:innen hatten in Studien nicht nur klinische Symptome, die mit einer Aktivierung von Mastzellen einhergehen [269], es konnten auch signifikant erhöhte Blutspiegel an Mastzell-spezifischen Proteasen detektiert werden [270].

Symptome, die typischerweise mit einer lokalen oder systemischen Mastzellüberaktivierung in Verbindung zu bringen sind:

- Haut (Urtikaria, Flush, Pruritus und/oder Angioödem)
- Respirationstrakt (verstopfte Nase, nasaler Pruritus, keuchende Atmung, ödematöse Schwellungen im Halsbereich und/oder Heiserkeit)
- Kardiovaskuläres System (Kopfschmerzen, Hypotension bis zur Synkope und/oder Tachykardie)
- Gastrointestinaltrakt (abdominale Krämpfe, Diarrhö und/oder Übelkeit) [271]

Die konsensuellen, diagnostischen Kriterien für ein Mastzellüberaktivierungssyndrom involvieren 1) typische, klinische Symptome einer schweren, wiederkehrenden, systemischen Mastzellaktivierung (häufig Anaphylaxie), 2) labordiagnostische Dokumentation der Mastzellaktivierung und 3) Ansprechen auf Mastzell-stabilisierende und/oder Mastzellmediator-blockierende Medikamente [272].

##### Empfehlung

Im Idealfall soll bei Patient:innen mit Symptomen, die auf eine Mastzellüberaktivierung hindeuten, eine Abklärung entsprechend den verfügbaren internationalen Konsensusprotokollen [271, 273] im spezialisierten niedergelassenen Bereich oder an spezialisierten Zentren in die Wege geleitet werden.

#### 8.8.5 Spezifische Indikationen für die immunologische Abklärung – Zusammenfassung

Grundsätzlich gilt: andere Ursachen für PASC müssen ausgeschlossen sein. Die immunologische Abklärung soll nach korrekter Indikationsstellung im spezialisierten Bereich stattfinden (Tab. 1).

**Table 1:** 

Indikation	Symptombild	Befunde	Immunologische Abklärung
PASC *und* schwere rezidivierende Infekte	PASC, z. B. chronische Fatigue und infektassoziierte Symptome	Blutbild inklusive Differenzialblutbild, Entzündungsmarker	Immunglobulin und Subklassen, Evaluierung Komplementsystem, Lymphozytensubtypisierung mittels FACS, ggf. funktionelle Lymphozytenanalyse
Immunsupprimierte oder immundefiziente Personen mit PASC *und* Milz‑, Lymphknoten- und/oder Lebervergrößerung	PASC und infektassoziierte Symptome	–	Abklärung auf akute/chronische Infektionen (u. a. auch SARS-CoV-2-Persistenz oder Re-Infektion; u. a. EBV- oder CMV-Reaktivierung); alternative Diagnosen
Hinweise auf Mastzellüberaktivierung, Unverträglichkeitsreaktionen gegen Nahrungsmittel und/oder Medikamente	Kardiovaskuläre, kutane, respiratorische und/oder gastrointestinale Leitsymptome entsprechend den internationalen Konsensusprotokollen	Leitsymptome erheben, Allergieabklärung Tryptase-Werte bis zu 6 h nach dem Beginn akuter Beschwerden	Weitere Abklärung entsprechend den verfügbaren internationalen Konsensusprotokollen

### 8.9 Postvirale Fatigue

#### 8.9.1 Allgemeines

Dieses Kapitel befasst sich nicht mit dem häufigen Phänomen der Müdigkeit, Erschöpfung und Abgeschlagenheit, die viele Ursachen haben kann, und auch nicht selten als Folge einer Vielzahl von viralen Infekten auftritt. Die Differenzialdiagnostik dieses Symptoms findet sich in Abschn. 11.1. Siehe dazu auch die Leitlinie Müdigkeit der DEGAM [12].

Postvirale Fatigue ist nicht nur Müdigkeit oder Erschöpfung, sondern bezeichnet eine „zu den vorausgegangenen Anstrengungen unverhältnismäßige, durch Schlaf nicht zu beseitigende körperliche, geistige und/oder seelische Erschöpfung“ [274].

Fatigue ist das am häufigsten beschriebene Long-COVID-Symptom. Sie kann als leitendes oder begleitendes Symptom vorkommen. In den Erhebungen des Office for National Statistics (ONS; UK) wird die Häufigkeit unter den Personen mit selbst berichteten anhaltenden Folgen von COVID-19 (2,9 % der zu Hause lebenden Personen) mit 72 % angegeben [275]. Eine kanadische Studie berichtet 72 % Anteil von Fatigue an Symptomen, wobei auch Personen eingeschlossen waren, bei denen eine Infektion nicht gesichert war [276]. Zu ähnlichen Ergebnissen kam eine Umfrage aus Österreich vom April 2022 [277].

Auch nach anderen Viruserkrankungen (u. a. Influenza, EBV, CMV, Masern, Ebola, West-Nil-Virus, andere Corona-Viren) wird länger anhaltende postvirale Fatigue beobachtet [76, 78, 254, 278]. Hypothesen zu den Ursachen sind auch hier vielfältig, s. dazu Kap. 7. Neben immunologischen und inflammatorischen Mechanismen sowie einer Viruspersistenz werden auch eine mitochondriale Dysfunktion und reduzierte muskuläre Energieversorgung diskutiert [90, 279].

Zu beachten ist, dass Fatigue auch Ausdruck organisch-struktureller Folgeschäden von COVID-19 sein kann (z. B. Sauerstoffmangel durch [atypische] Lungenfibrose oder im Rahmen einer Herzinsuffizienz). Die ist differenzialdiagnostisch unbedingt zu berücksichtigen (Abschn. 11.1).

##### Empfehlung

Postvirale Fatigue muss von organisch strukturellen Störungen nach COVID oder aus alternativer Ursache (physischer, psychischer oder sozialer Natur) sowie von postinfektiöser Müdigkeit und Erschöpfung im Rahmen der normalen Rekonvaleszenz unterschieden werden. Eine exakte Differenzialdiagnostik ist unerlässlich.

#### 8.9.2 Postvirale Fatigue in Zusammenhang mit COVID‑19

##### 8.9.2.1 Post-Exertional-Malaise (PEM; auch: „post-exertional symptom exacerbation“ [PESE])

Bei postviraler Fatigue ist auf das Vorhandensein einer Post-Exertional-Malaise (PEM) zu achten: PEM ist abzugrenzen von einfachen Belastungsintoleranzen im Sinne von mangelnder körperlicher Belastbarkeit durch organische Erkrankungen oder bei protrahierten Rekonvaleszenzverläufen. PEM ist eine „nach (auch leichter) Alltagsanstrengung auftretende **Verschlechterung der Beschwerden, die oft erst nach einer Zeitverzögerung von mehreren Stunden oder am Folgetag einsetzt, mindestens bis 14** **h nach Belastung noch spürbar ist und oft mehrere Tage (bis Wochen) und im schlimmsten Fall dauerhaft anhält**. Auslöser können dabei sowohl körperliche, kognitive als auch emotionale, sensorische oder orthostatische Belastungen sein“ [274]. Es gibt mehrere, jedoch aufwendige objektivierbare Untersuchungsmethoden, die aber auch für die PatientInnen höchst belastend sind, wenn nicht zu starker Verschlechterung führen und nicht in der hausärztlichen Versorgung durchgeführt werden können. So können kardiopulmonale Belastungstests (CPET) an 2 aufeinanderfolgenden Tagen eine PEM objektiv darstellen. Auch ließ sich während eines orthostatischen Stresstests bei 90 % der Patienten (*n* = 429) im Vergleich zu Gesunden eine deutliche Reduktion der per Doppler gemessenen Hirndurchblutung nachweisen [280, 281]. Die Verwendung eines geeigneten Fragebogens [282] ist zusammen mit einer sorgsamen Anamnese empfohlen. Sie ist von hoher Relevanz, weil sie das weitere Vorgehen determiniert. Bei Vorliegen von PEM kann aufgrund der nachgelagerten Zustandsverschlechterung eine Aktivierungstherapie nicht erfolgen, sondern es ist die Therapieform [11, 283] des Pacings zu wählen (s. dazu Abschn. 12.2.1) [274, 284]. Bei Fehlen von PEM/PESE ist dagegen die Aktivierungstherapie im Sinne von „graded exercise“ der angemessene Zugang (s. dazu 12.2.2). Die Abgrenzung ist nicht immer scharf, daher ist ein hochgradig individualisierter Behandlungsplan erforderlich, der, wenn möglich, unter Einbeziehung weiterer Gesundheitsberufe, wie z. B. Ergotherapie, umgesetzt werden sollte.

PEM kann auch unabhängig von einer Fatigue-Symptomatik auftreten, auch in Kombination mit anderen Symptomen. Hinsichtlich eines kombinierten Auftretens von PEM/PESE mit Störungen des autonomen Nervensystems (ANS) ist die Studienlage noch nicht eindeutig.

##### 8.9.2.2 Fatigue als Einzelsymptom (ohne PEM/PESE)

Als Einzelsymptom wird Fatigue oft im Verlauf von einigen Wochen bis Monaten besser oder verschwindet vollständig, in diesem Fall ist nach Ausschluss von Red Flags und akut behandlungsbedürftigen Differenzialdiagnosen abwartendes Verhalten mit Ermunterung zur langsamen, symptomgesteuerten Aktivierung die Therapie der ersten Wahl (s. dazu 11.1). Postvirale Fatigue ist aber auch oft mit anderen Symptomen vergesellschaftet, dann sind zusätzliche Punkte zu beachten [274].

##### 8.9.2.3 Fatigue in Zusammenhang mit orthostatischer/autonomer Dysregulation

Wenn postvirale Fatigue durch orthostatische/autonome Dysfunktion (AD) bedingt ist, wie z. B. das posturale Tachykardiesyndrom (POTS), die orthostatische Hypotonie (OH) und/oder Tachykardie und Palpitationen (s. dazu 8.10), sollte dies rasch erkannt und behandelt werden (s. dazu 11.9 und 12.3.3). Eine autonome Dysfunktion kann sich u. a. auch als Reizdarm oder Reizblase äußern. Die Etablierung einer Therapie nach Ausschluss organisch-struktureller oder mentaler Ursachen (s. dazu 11.1 und 11.9) kann die Lebensqualität der Betroffenen deutlich steigern. [13, 274] (s. dazu 12.3.3).

##### 8.9.2.4 Weitere wichtige in Kombination mit Fatigue auftretende postvirale Symptome

- Eine im Vergleich zu der Zeit vor der Erkrankung substanzielle Einschränkung der Fähigkeit zu beruflichen, schulischen, sozialen oder persönlichen Aktivitäten mit plötzlichem oder definiertem Beginn [20, 285, 286] (s. auch Leitlinie Ergotherapie, Anhang)
- Nicht erholsamer Schlaf, Einschlaf- und/oder Durchschlafstörungen, gekippter oder rollierender Schlaf-Wach-Rhythmus
- Kognitive Dysfunktionen, wie z. B. Konzentrations- und Gedächtnisstörungen, Wortfindungsstörungen, Reizüberempfindlichkeit
- Neurogene Schmerzen, Kopfschmerzen, Muskel‑/Gelenkschmerzen
- Neuroendokrine Störungen, wie z. B. gestörte Anpassung der Körpertemperatur, Temperaturempfindlichkeit, Appetitstörung
- Zeichen einer Immundysregulation, wie z. B. Symptome, die zum Konzept einer Mastzellaktivierung passen [269, 270, 287]. (Zu diesem Konzept siehe 8.8.) Symptome sind z. B. Blähungen, Reizdarm, neu aufgetretene Unverträglichkeiten oder Allergien, Juckreiz, Herzrasen oder „Infekt-Symptome“ wie Verstopfung der Nase, Grippegefühl, Halsschmerzen bzw. eine Rötung der Bindehaut bzw. Sehstörungen oder aber Immundysregulation im Sinne einer Infektionsneigung.
- Angststörungen und/oder depressive Störungen (s. dazu auch 8.6) [206, 207]. Diese Störungen können durch die im Kap. 7 beschriebenen viralen und postviralen Prozesse direkt oder indirekt durch SARS-CoV‑2 getriggert werden. Sie können aber auch als depressive Störungen bzw. als Folge der Beanspruchung durch die anhaltende postvirale Symptomatik im Sinne einer sekundären somatischen Belastungsstörung zu verstehen sein. Zudem kommt es auch in der Allgemeinbevölkerung im Gefolge der Pandemie sekundär [288] zum vermehrten Auftreten von mentalen Belastungen.
- Siehe dazu auch Kedor et al. [289], Sudre et al. [8], Sonnweber et al. [108]

All diese Symptome können im Rahmen einer PEM nach Aktivität/Überlastung verschlechtert werden oder neu auftreten, sie können aber auch ohne PEM vorhanden sein.

Wir weisen darauf hin, dass Überschneidungen mit dem Konzept der ME/CFS (myalgische Enzephalomyelitis/chronisches Fatigue-Syndrom) vorhanden sind [290]. Laut AWMF [13] und NICE [18] Guidelines zu Long COVID sollte daran gedacht werden, wenn bei PatientInnen im Alter unter 60 Jahren schwere Fatigue mit PEM, kognitiven Störungen und Schmerzen auftreten und diese für mehr als 6 Monate bestehen bleiben.

Die Leitliniengruppe merkt dazu an, dass zuvor eine leitlinienkonforme differenzialdiagnostische Abgrenzung anderer Symptomursachen (s. dazu 11.1), die einer Behandlung zugänglich sein könnten (z. B. einer autonomen Dysfunktion oder einer mentalen Problematik), stattgefunden haben muss, damit betroffene Personen keinen Nachteil hinsichtlich möglicher therapeutischer Zugänge erleiden.

Nachdem der als ME/CFS beschriebene Zustand jedoch auch durch weitere Ursachen als postvirale ausgelöst werden kann und nur eine (seltene) der vielen möglichen Zustände nach einer SARS-CoV-2-Infektion ist, wird in dieser Leitlinie nicht näher darauf eingegangen. Für detailliertere Informationen zum Thema ME/CFS verweisen wir aus diesem Grund auf die S3-Leitlinie der DEGAM [12] zur Müdigkeit, die auch das Thema ME/CFS behandelt.

#### 8.9.3 Fatigue ohne Zusammenhang mit COVID-19

Fatigue kann ein Symptom für nicht im Zusammenhang mit einer Infektion stehende Erkrankungen sein, wie z. B. Anämie, Hypothyreose, Intoxikationen, Schlafstörungen oder onkologische Erkrankungen [291, 292].

Eine exakte Differenzialdiagnostik ist daher obligat, s. dazu 11.1.

Zur Differenzialdiagnostik Müdigkeit/Fatigue: S3-Leitlinie „Müdigkeit“ der DEGAM [12]

Zur Behandlung der postviralen Fatigue siehe 12.3.2 sowie 12.2.1 (Pacing) und 12.2.2 (Coping)

### 8.10 Dysfunktionen des autonomen Nervensystems (ANS)

#### 8.10.1 Allgemeines

Neurologische bzw. neuroautonome postvirale Syndrome sind seit über 100 Jahren kontinuierlich beschrieben und auch in den letzten Jahrzehnten gut untersucht. Im Rahmen einer viralen Pandemie war daher ein gehäuftes Auftreten zu erwarten. Das Wissen um Komplikationen des autonomen Nervensystems ist derzeit noch anekdotisch.

Ein in Literaturreview identifizierte 134 Patient:innen mit kardiovaskulärer autonomer Funktionsstörung in zeitlichem Zusammenhang mit SARS-CoV-2: davon 81 während der akuten SARS-CoV-2-Erkrankung und 53 in der Post-COVID-Phase [293]. Personen mit neuroautonomen Komplikationen in der Phase nach COVID waren im Vergleich zur Akutphase jünger (42 vs. 51 Jahre, *p* = 0,002) und häufiger weiblichen Geschlechts (68 % vs. 49 %, *p* = 0,034).

Am häufigsten trat Reflexsynkope als kardiovaskuläre Erkrankung in der Akutphase auf, bei Post-COVID-Patient:innen das posturale Tachykardiesyndrom (POTS) zu 62 %, gefolgt von der orthostatischen Hypotonie (OH) zu 15 %.

Patient:innen, die während der Akuterkrankung eine autonome Funktionsstörung entwickelten, zeigten in 43 % eine komplette Remission, dies war nur in 15 % der Post-COVID-Patient:innen zu beobachten (*p* = 0,002). Darüber hinaus zeigten 56 % neuroautonome Post-COVID-Patient:innen eine Teilremission ihrer Beschwerden. Die Initialinfektion mit SARS-CoV‑2 war bei Patient:innen mit neuroautonomer Folgeerkrankung großteils mild (67 % – moderat: 11 %, schwer: 22 %).

Zusammenfassend fällt auf, dass Patient:innen, die nach der SARS-CoV-2-Infektion eine autonome Erkrankung entwickeln, in der Akutphase häufig milde Symptome hatten, jünger und eher weiblich waren.

#### 8.10.2 Autonome Funktionsstörungen nach COVID – Erscheinungsformen

##### 8.10.2.1 Kardiovaskuläre autonome Funktionsstörungen

Am häufigsten trat Reflexsynkope als kardiovaskuläre Erkrankung in der Akutphase auf, bei Post-COVID-Patient:innen das posturale Tachykardiesyndrom (POTS) zu 62 %, gefolgt von der orthostatischen Hypotonie (OH) zu 15 %.

Viele Patient:innen mit einer kardiovaskulären Funktionsstörung erleben einen Bewusstseinsverlust (Synkope bzw. in der englischen Literatur „transient loss of consciousness“ [TLOC]). Der Fokus der ärztlichen Anamnese liegt darauf, von so vielen Episoden/Ereignissen wie möglich Informationen zu bekommen. Wichtig zur Anamneseunterstützung sind Begleitpersonen, insbesondere wenn sie einzelne Episoden beobachtet haben. Dezidiert nachgefragt sollte auch nach Videoaufzeichnungen werden, die heutzutage häufig verfügbar sind. Bei kardiovaskulären Funktionsstörungen sind die Beschwerden beim aufrechten Stehen deutlich verstärkt und sistieren im Liegen. Einzelne Symptome sollten in so einer Situation abgefragt werden (z. B. Unscharfsehen, Schwindel etc.). Begleitumstände geben oft wichtige Hinweise. So können Beschwerden am frühen Morgen (durch Nykturie), während der Menstruation, nach einem kohlenhydratreichen Essen bzw. nach langem Stehen verstärkt auftreten.

In der Folge wird auf die 2 häufigsten neuroautonomen kardiovaskulären Funktionsstörungen eingegangen: POTS und OH.

##### 8.10.2.2 POTS (posturales orthostatisches Tachykardiesyndrom)

POTS wurde erstmals 1982 von Rosen und Cryer beschrieben und hat insbesondere in den letzten Jahren im Zusammenhang mit der SARS-CoV-2-Pandemie neue Aufmerksamkeit erlangt.

POTS ist keine Entität, sondern ein klinisches Syndrom, bei dem in Orthostase unterschiedliche Symptome aufgrund einer sympathischen Überaktivität auftreten.

Das häufig auftretende wiewohl früher wohl unterdiagnostizierte POTS-Syndrom spielt auch bei der aktuellen viralen Pandemie eine gewichtige Rolle, des Weiteren ist aber eine orthostatische Hypotonie auch zu bedenken. Bisher gibt es noch keinen klaren Hinweis, ob POTS nach SARS-CoV‑2 (dzt. „Long-COVID-POTS“) sich in irgendeiner Form von POTS nach anderen viralen Infekten unterscheidet – und es damit überhaupt eine Berechtigung gibt, hierbei von „Long-COVID-POTS“ zu sprechen [294].

**Figure 4:**
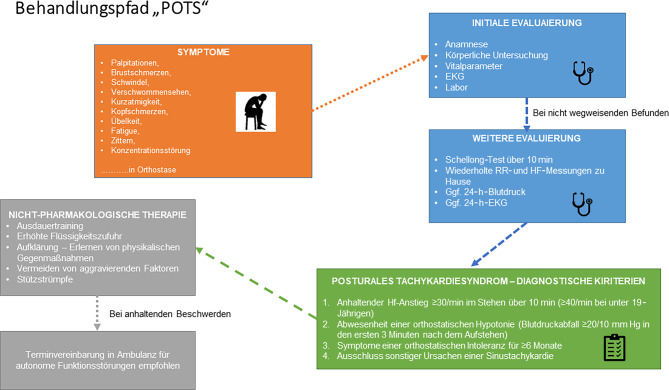

Zu denken an das Syndrom ist, wenn Symptome wie Palpitationen, Brustschmerzen, Benommenheit, Verschwommensehen, Kurzatmigkeit, Kopfschmerzen, Übelkeit, Fatigue, Zittrigkeit, Belastungsintoleranz und Konzentrationsstörungen im Stehen auftreten. Der wesentliche Aspekt ist dabei, dass diese Beschwerden im Liegen sistieren (Abb. 4).

Pathophysiologisch wird die Sympathikusüberaktivierung durch unterschiedliche Mechanismen wie Hypovolämie, autoimmunologische Prozesse, Dekonditionierung und autonome Neuropathie ausgelöst durch u. a. (vor allem virale) Infektionen, Traumata und Stress.

Zur Abgrenzung des POTS von anderen Phänomenen, wie z. B. Angsterkrankungen, s. Abschn. 8.6. Anzumerken ist, dass eine kartesianische Dichotomie von Psyche und Körper wenig hilfreich ist, um eine Multisystemregulationsstörung wie POTS zu begreifen, und zwar von beiden Seiten des Behandlungstisches aus. Bei Verdacht auf ein POTS soll initial eine ausführliche Anamnese inklusive Erhebung möglicher Trigger, Auswirkung auf den Alltag, Vorerkrankungen und Medikation erhoben werden. Des Weiteren soll eine körperliche Untersuchung stattfinden, Vitalparameter sollen dokumentiert, ein EKG soll geschrieben und ein Basislabor abgenommen werden. Bei weiter bestehendem Verdacht eines POTS kann dieser mittels Schellong-Test (Abb. 2) über 10 min im Stehen erhärtet werden.

Hämodynamische Kriterien für POTS sind ein beständiger Herzfrequenzanstieg ≥ 30 Schläge/min im Stehen über 10 min (≥ 40/min bei unter 19-Jährigen). Bei Patient:innen mit niedriger Liegend-Herzfrequenz (< 60 Schläge/min) kann die Differenz von einer Ruhefrequenz von 60 Schlägen/min berechnet werden [295]. Die Kriterien müssen tatsächlich durch zumindest 2 der Messungen erfüllt werden, die mindestens 1 min zeitlichen Abstand voneinander haben und deren Trend die Sympathikusaktivierung bei POTS widerspiegelt. Ein zeitgleiches Auftreten einer orthostatischen Hypotonie (Blutdruckabfall ≥ 20/10 mm Hg in den ersten 3 min nach dem Aufstehen) schließt ein POTS aus.

Sonstige Ursachen einer Sinustachykardie müssen ausgeschlossen werden. Ebenso schließen andere Homöostasebedingungen, die die orthostatische Tachykardie erklären, POTS per Definition aus: Anämie, Angst, Fieber, Schmerz, Infektion, Dehydratation, Hyperthyreose, Phäochromozytom, längere Bettruhe sowie Verwendung von Medikation, die die Herzfrequenz hebt (Stimulanzien, Diuretika, Noradrenalin-Wiederaufnahmehemmer) (s. unten).

##### Empfehlung

Bei Symptomen wie Palpitationen, Brustschmerzen, Benommenheit, Verschwommensehen, Kurzatmigkeit, Kopfschmerzen, Übelkeit, Fatigue, Zittrigkeit und Konzentrationsstörungen sollte an ein posturales orthostatisches Tachykardiesyndrom (POTS) gedacht werden, wenn diese Symptome im Stehen auftreten und im Liegen sistieren.

Diagnosekriterien (alle müssen erfüllt sein) [296]:

- Beständiger Herzfrequenzanstieg ≥ 30 Schläge/min im Stehen über 10 min (≥ 40/min bei unter 19-Jährigen) innerhalb 10 min aufrechter Haltung mit Auftreten der Beschwerden
- Abwesenheit einer signifikanten orthostatischen Hypotonie (Blutdruckabfall ≥ 20/10 mm Hg)
- Häufige Symptome von orthostatischer Intoleranz, verstärkt in aufrechter Haltung und rascher Besserung in liegender Haltung. Symptome können sehr individuell sein, beinhalten aber häufig Benommenheit, Palpitationen, Zittern, generelles Schwächegefühl, Unscharfsehen, Erschöpfung
- Symptomdauer ≥ 3 Monate
- Andere Ursachen, die eine Sinustachykardie im Stehen erklären könnten, müssen ausgeschlossen sein.

Andere Ursachen, die eine Sinustachykardie im Stehen erklären könnten und mit der Diagnose POTS nicht vereinbar sind [296]:

- Akute Hypovolämie (als Folge von Dehydratation oder Blutverlust)
- Anämie
- Orthostatische Hypotonie
- Endokrinopathie
- Nebenniereninsuffizienz
- Karzinoid
- Hyperthyreose
- Phäochromozytom
- Nebenwirkungen von Medikation (mitunter auch Nahrungsergänzungsmittel, Naturstoffe)
- Panikattacken und heftige Angst
- Längere oder dauerhafte Bettruhe
- Effekte von Freizeitdrogen

Zum Management des POTS s. Abb. 4, sowie Absatz 12.3.3.1).

##### 8.10.2.3 OH (orthostatische Hypotonie)

Aus Übersichtsgründen wird in diesem Text lediglich die sog. klassische orthostatische Hypotonie betrachtet.

Bei Zeichen einer orthostatischen Hypotonie hat es sich bewährt, als Erstes die Medikation zu reevaluieren. Häufig finden sich Diuretika, Antihypertensiva inklusive Betablocker bzw. Nitrate, deren Verschreibung noch einmal kritisch reevaluiert werden sollte.

Jedenfalls ist entscheidend, sich mit der Diagnose OH nicht zufriedenzugeben und Anstrengungen zu unternehmen, nach der Ursache der OH zu fahnden. Häufige Ursachen für neurogene OH (nOH) sind Parkinson-Erkrankungen, Demenzerkrankungen. Des Weiteren sollte man fahnden nach sekundären nOH-Ursachen wie metabolische Erkrankungen (DM, Urämie), immunmediierte neurologische Erkrankungen (GBS, autoimmune autonome Ganglionopathie), Amyloidose oder Paraneoplasie.

Zum Management der OH siehe Absatz 12.3.3.2.

##### 8.10.2.4 Sudomotorische Störungen

Dabei ist zu beachten, dass Patient:innen meistens wegen vermehrten Schwitzens ärztlichen Rat suchen, wiewohl dies neben dem Gedanken an die Hyperhidrose auch Anlass geben sollte, die Hypohidrose zu bedenken. Auch bei Hypohidrose kann es zu vermehrtem kompensatorischem Schwitzen an nicht betroffenen Hautarealen kommen.

##### 8.10.2.5 Gastrointestinale Beschwerden, pupillomotorische Beschwerden, urogenitale sowie Beschwerden der Sexualfunktion

**Figure 5:**
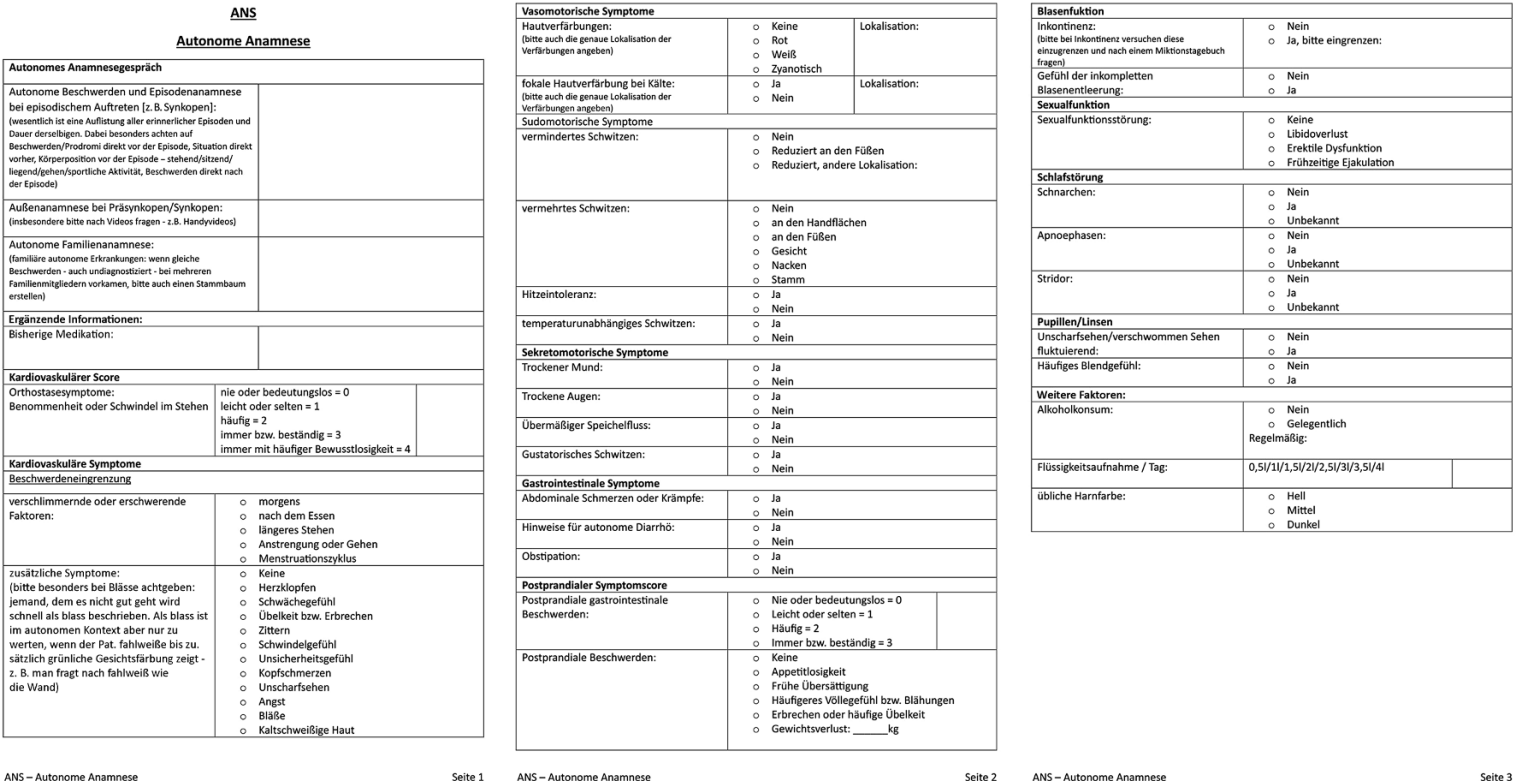

Nach diesen Beschwerden sollte aktiv gefragt werden. Gerade wenn man diese Anamnese nicht häufig macht, können einzelne Anamnesepunkte übersehen werden, s. dazu das online verfügbare Anamnesetool (Abb. 5.).

##### Empfehlung

Bei orthostatischer Intoleranz, sudomotorischen Symptomen, Beschwerden aus dem gastrointestinalen, urogenitalen und sexuellen Bereich sollte an eine autonome Dysfunktion im Rahmen eines postviralen Zustands gedacht werden. Andere Ursachen dafür müssen ausgeschlossen werden.

#### 8.10.3 Neuroautonome Diagnostik nach COVID-19

Die Funktionsdiagnostik beginnt mit einer ausführlichen Anamneseerhebung [297]. Die speziellen Aspekte hierbei werden bei den Erscheinungsbildern unter Absatz 8.10.2 beschrieben. Das ANS-Anamnesetool (Abb. 5) dient zur Unterstützung.

Eine detaillierte Anamneseerhebung ist sehr hilfreich, um 2 Fragen zu beantworten:

1. Handelt es sich um eine autonome Funktionsstörung?
2. Welche autonomen Domänen sind betroffen?

Die ärztliche Diagnostik erfordert eine konsensus- bzw. evidenzbasierte Durchführung, um zu einer Diagnose zu führen. Insbesondere bei kardiovaskulären autonomen Erkrankungen sollte dem Rechnung getragen werden, da immer mehr an Patientinnen generierte Daten z. T. von Consumer-Produkten Ärzt:innen zur Kenntnis gebracht werden, die die sorgfältige Diagnostik nicht ersetzen dürfen. Die Untersuchung sollte unter vergleichbaren Bedingungen (Raumtemperatur, Vermeidung von Störungen) durch eine erfahrene Untersucher:in durchgeführt werden. Die ärztliche Dokumentation der medizinisch durchgeführten Untersuchung muss die darauf fußende Diagnose nachvollziehbar machen.

Im europäischen Kontext wurden bisher bei Verdacht auf kardiovaskuläre neuroautonome Funktionsstörung nach COVID-19 von autonomen Funktionslaboren am häufigsten der aktive Stehtest (auch Schellong-Test, Abb. 2), Valsalva, tiefe Atmung, Kipptischuntersuchung, Kälteprovokation, isometrische Anstrengung durchgeführt, wobei am häufigsten der Kipptisch, Valsalva und der aktive Stehtest angewendet wurden.

**Figure 6:**
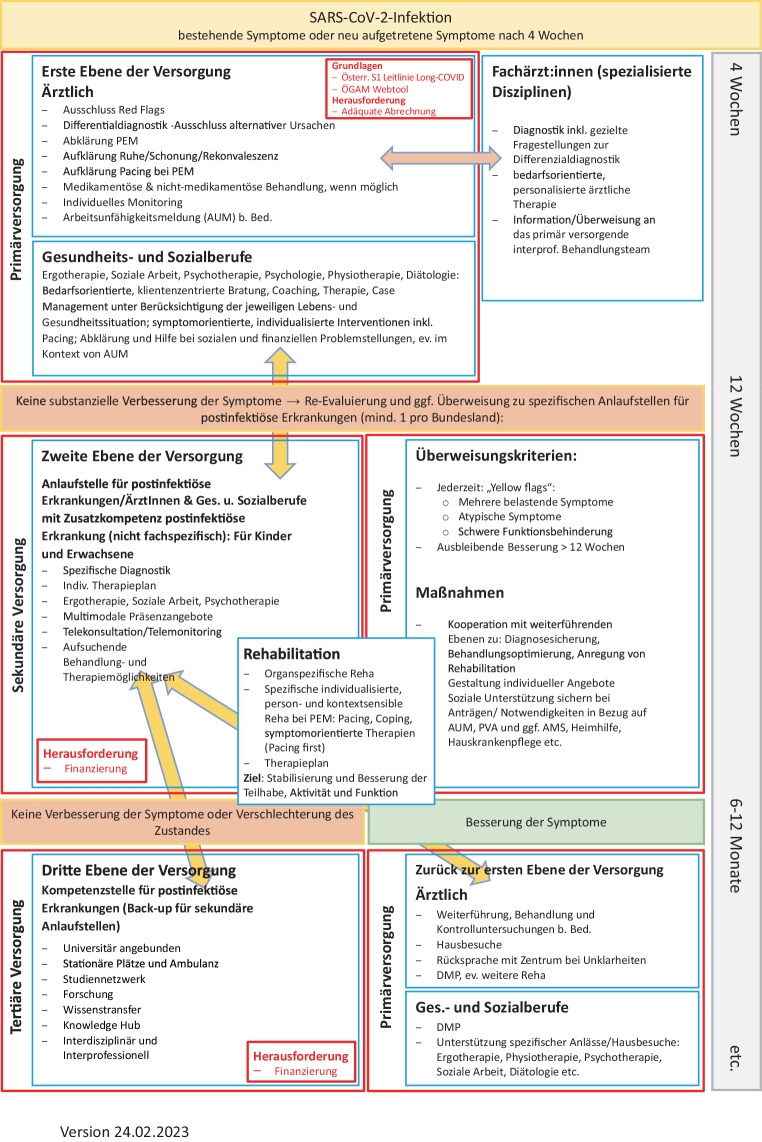

Im Folgenden wird die Abklärung signifikanter bzw. länger als 12 Wochen anhaltender Beschwerden beschrieben, die meist innerhalb der spezialisierten Versorgung durchgeführt werden.

(Zu den Tests im Rahmen der Erstabklärung im hausärztlichen/primärversorgenden Bereich sowie Überweisungszeitpunkten s. 11.9 und Versorgungswege Kapitel 9, Abb. 6)

##### 8.10.3.1 Kardiovaskuläre neuroautonome Funktionsdiagnostik

Eine EKG-Untersuchung und die Erhebung eines neurologischen Status ermöglichen die Identifikation von rhythmogenen Herzerkrankungen sowie neurologischen Erkrankungen wie neurodegenerative Erkrankungen (Morbus Parkinson, Parkinson-Syndrome, Demenzerkrankungen und andere) als beschwerdeverursachend.

##### Einfacher Stehtest – Schellong-Test (Abb. 2)

Der Schellong-Test als Eponym geht auf den in Königsberg 1891 geborenen Fritz Schellong, Prof. für Innere Medizin in Prag und Münster, zurück (für einen schönen Überblick s. [298]).

Dieser Test eignet sich zum Screenen von Patient:innen und erfordert in einfachster Ausführung lediglich ein automatisches Blutdruckgerät. Eine Anleitung zur Durchführung der Untersuchung und ein geeignetes Formular zur Dokumentation findet sich in Abb. 2.

Wesentlich ist die Dokumentation auch der Beschwerden durch Ärztin/Arzt bzw. delegierten Mitarbeiter:in eines Gesundheitsberufes.

Diagnostische Kriterien für eine orthostatische Hypotonie sind ein abnormer Blutdruckabfall definiert als ≥ 20 mm Hg systolisch, ≥ 10 mm Hg diastolisch bzw. ein systolischer Blutdruckabfall < 90 mm Hg im Stehen [299]. Sollte Schlag für Schlag Blutdruckmessung verfügbar sein, ist diese der Riva-Rocci-Messung vorzuziehen, um sehr kurze Blutdruckschwankungen mitzuerfassen. Ob eine neurogene OH vorliegt kann mittels einer einfachen Berechnung evaluiert werden. Sollte es zu < 0,49 Schläge/min pro mm Hg Blutdruckabfall kommen, so ist von einer neurogenen OH auszugehen [300].

POTS sollte als Diagnose bzw. Differenzialdiagnose in Betracht gezogen werden, wenn innerhalb von 10 min Stehen ein Anstieg der Herzfrequenz um > 30 Schläge pro Minute bzw. über 120 Schläge pro Minute mit typischen Beschwerden ohne signifikanten Blutdruckabfall (i. S. einer OH) auftritt. Dabei sollte aber die physiologische Sinusarrhythmie ebenso in Betracht gezogen werden, die physiologischerweise bei höheren Herzfrequenzen zu höheren Auslenkungen führt. Daher sind mindestens 2 Werte gefordert, die mindestens im Abstand von 1 min gemessen werden sollten. Ein wesentlicher Aspekt ist, dass die Erfüllung dieser Kriterien nicht gleichbedeutend ist mit der Diagnose POTS. Die Diagnose POTS ist komplex (s. unten).

##### Kipptischuntersuchung (Sonderfälle)

**Figure 7:**
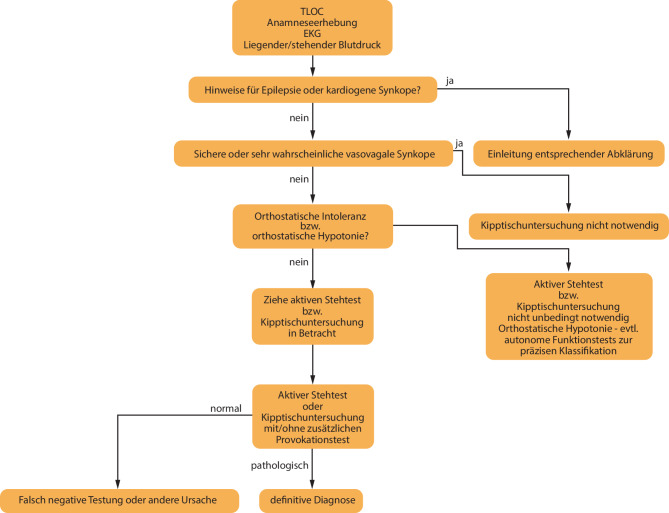

Eine detaillierte Beschreibung der Durchführung einer Kipptischuntersuchung findet sich im 2021 EFAS-Konsensus. Insbesondere bei rezidivierenden Bewusstseinsverlusten kann nach Abb. 7 vorgegangen werden. Diese gibt einen Entscheidungsbaum bzgl. Zuweisung zur Kipptischuntersuchung wieder.

Da es mehrere autonome Protokolle je nach Fragestellung für die Kipptischuntersuchung gibt, ist es sinnvoll, Patient:innen vor Durchführung der Untersuchung in der den österreichischen Kipptischlaboren angeschlossenen Spezialsprechstunde vorzustellen.

##### 8.10.3.2 Weitere Diagnostik bei Verdacht auf Dysfunktion des autonomen Nervensystems

Zusätzlich wurden indikationsbezogen auch sudomotorische Tests wie der quantitative sudomotorische Axonreflextest, der thermoregulatorische Schweißtest, „sympathetic skin response test“, Hautbiopsie, und kardiale 123I-MIBG-Szintigraphie durchgeführt.

Trotz der Vielzahl der einem autonomen Funktionslabor der Neurologie zur Verfügung stehenden Tests sind es vor allem kardiovaskuläre und gebräuchliche Tests, die bei postviralen Zuständen wie nach COVID-19-Anwendung finden und die auch durch Konsensus definiert sind [299, 301].

##### Empfehlung

Bei Symptomen autonomer Dysfunktion sollte jedenfalls ein Schellong-Test bereits an der Stelle des Erstkontakts durchgeführt werden.

##### Empfehlung

Differenzialdiagnostik und erste Funktionstests (Schellong-Test!) können und sollten bereits in der Primärversorgung stattfinden. Weiterführende Diagnostik und Erstellung eines Behandlungsplans erfolgen bei ausgeprägter Symptomatik bzw. Persistenz über 12 Wochen sinnvollerweise an einem Zentrum für autonome Dysfunktion.

- **Management** siehe dazu 12.3.3 Behandlung autonome Dysfunktion

##### 8.10.4 Kommunikation von autonomen Erkrankungen

Das Wissen um die Pathophysiologie v. a. bei POTS ist noch limitiert. POTS und OH sind Syndrome, welche jeweils Endstrecken sehr unterschiedlicher Pathomechanismen darstellen.

Daher ist in der Beratung vorrangig, beide Diagnosen nicht als Antwort auf alle Fragen, sondern eher als Beginn einer langen Reise darzustellen.

Insbesondere sind Patient:innen oftmals teilinformiert über Informationsquellen online. Pathophysiologische Sub-Syndrome basierend auf Labordiagnosen sind von klinisch-wissenschaftlichem Interesse, zum Teil aber irreführend („hyper-POTS“) und führen manchmal zu verzweifelten Versuchen von Patient:innen, alle ihre Beschwerden einer einzigen Diagnose zuzuordnen.

Es kann hilfreich sein, individuelle Beschwerden in den Mittelpunkt des Managements zu stellen, wie bei anderen chronischen Erkrankungen – und von „POTS“ bzw. „OH“ als Schlagwort wegzukommen.

Dazu gehört auch, dass POTS nicht als primäre Diagnose festzustellen ist, sondern immer Versuche zu unternehmen sind, die zugrunde liegende Erkrankung zu identifizieren (z. B. „Kleinfaserneuropathie nach SARS-CoV‑2 Infektion [Monat]/22 assoziiert mit POTS“, „SARS-CoV‑2 Infektion [Monat]/22 – postvirales Syndrom unklarer Signifikanz assoziiert mit POTS“ etc.).

Dadurch bekommen PatientInnen, aber auch Zuweiser:innen ein klareres Bild, dass autonome Krankheitsbilder mit einem individuellen komplexen Krankheitsprozess assoziiert sind.

##### Autonome Fachlabore in Österreich

In Österreich gibt es 4 neuroautonome Speziallabore, die allerdings nun durch die Pandemie durchwegs eine erhöhte Patient:innenfrequenz haben.

## 9. Versorgungsweg postvirale Erkrankungen am Beispiel der SARS-CoV-2-Infektion

### 9.1 Vorbemerkungen

International besteht Übereinkunft [130, 214, 302, 303], dass die primäre Abklärung und die Basisbetreuung als Aufgabe der Primärversorgungsebene zu sehen ist. Für Österreich gilt bis dato der Versorgungspfad des BMSGPK, der die Rollenverteilung in der Versorgung von Patient:innen mit Folgen von COVID-19 festlegt [304].

### 9.2 Empfehlungen zum österreichischen Versorgungspfad

Die folgenden Empfehlungen orientieren sich an den internationalen Empfehlungen, wurden aber an die Gegebenheiten des österreichischen Gesundheitssystems und die rezenten Ergebnisse aus Studien zum COVID-19-Verlauf angepasst [30].

Damit soll der Versorgungspfad des BMSGPK [304] konkretisiert und mit spezifischen Algorithmen und Handlungsempfehlungen unterlegt werden. Einbezogen in die Konkretisierung wurden auch erste Erfahrungen aus bisherigen Versorgungsstrukturen wie den wenigen Post-COVID-Clearingstellen und Ambulanzen in Österreich – aus denen Lehren gezogen werden müssen, auch wenn derzeit belastbare Evaluierungen noch fehlen. Wir folgen damit dem Prinzip der Verwendung der besten verfügbaren Evidenz, denn zumindest vorläufige Entscheidungen zur Betreuung der Betroffenen müssen getroffen werden.

#### Empfehlung

Patient:innen sollte geraten werden, bei Beschwerdepersistenz über 4 Wochen eine Abklärung in Anspruch zu nehmen. Erstabklärung und differenzialdiagnostische Zuordnung von Symptomen, die in zeitlichem Zusammenhang mit viraler Infektion aufgetreten sind, sollen in der hausärztlichen Primärversorgung erfolgen, ebenso wie die primäre Betreuung und Begleitung.

#### 9.2.1 Aufgaben und Kompetenzen der Primärversorgung

##### Überblick

Die hausärztliche Primärversorgung ist aufgrund ihrer Fachdefinitionen (DEGAM [305], WONCA [306]) am besten dafür geeignet: sowohl für die Abklärung als auch für eine erste Behandlungsplanung bzw. die gezielte Weiterleitung an geeignete Kooperationspartner (Sonderfächer, Medizinberufe, weitere Gesundheits- und Sozialberufe und Beratungsstellen) [307].

**Figure 8:**
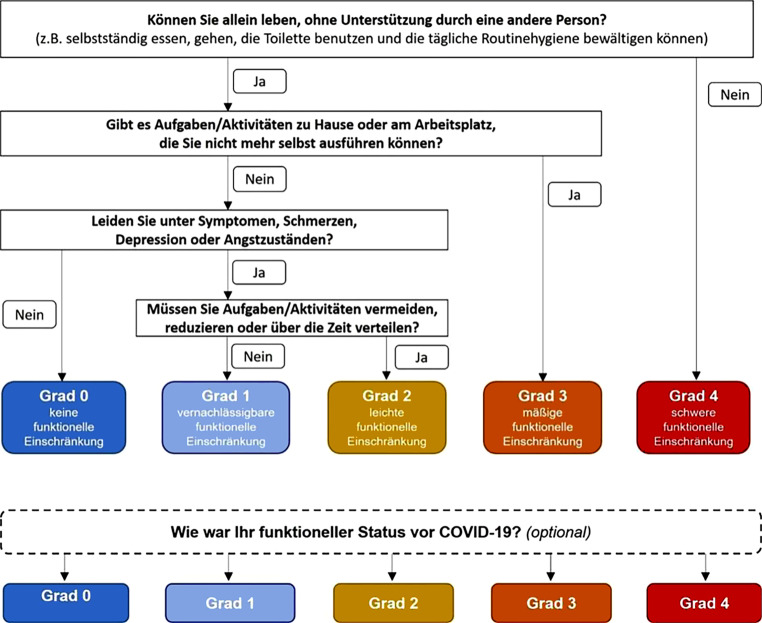

- Die Erstabklärung nach guter hausärztlicher Praxis (s. Differenzialdiagnostik, Kapitel 10 und 11.1) mit Veranlassung gezielter spezialistischer Abklärung im Bedarfsfall (z. B. Verdacht auf kardiale Erkrankung) ermöglicht Bestätigung oder Ausschluss organisch-struktureller und/oder psychiatrischer Erkrankungen und damit deren zeitgerechte Behandlung.
- Die hausärztliche Erstabklärung ermöglicht die Einschätzung von notwendiger begleitender sozialer, psychischer oder ergotherapeutischer Unterstützung.
- Sie ermöglicht die frühe korrekte Behandlung von Symptomen, die ursächlich der abgelaufenen Infektion mit SARS-CoV‑2 zugeordnet werden konnten, aber primär keinen organisch-strukturellen Schäden zugeordnet werden können:
  - Eine kausale Therapie ist hier zwar noch nicht möglich, symptomatisch kann den Patient:innen jedoch oft bereits von Anfang an geholfen werden.
  - Behandlungsoptionen sind Maßnahmen zu Pacing (siehe 12.2.1) und Coping (siehe 12.2.2) bei Symptomen von Fatigue mit Zeichen einer Post-Exertional-Malaise (PEM) oder PEM alleine, Aktivierungstherapie bei Fatigue ohne PEM/PESE (siehe 12.2.2) sowie die symptomatische Therapie postviraler Zustände (siehe 12.3.3). Erstlinientherapie im Regelfall meist nicht-medikamentös (siehe 12.1.1).
  - Zweitlinie: medikamentöse Optionen siehe 12.1.3
  - Wie bei Beschwerden anderer Art ohne derzeit noch gesichert nachweisbare alternative Ursache liegt der Schwerpunkt der multiprofessionellen Versorgung auf Kommunikation, Selbstwirksamkeit und kontinuierlicher individualisierter symptomatischer Betreuung, die ebenfalls im primärversorgenden Bereich geleistet werden kann [18, 302].
- Das Monitoring von Symptomverläufen, v. a. wenn eine Beeinträchtigung des alltäglichen Lebens besteht (z. B. Post-COVID-19-Skala des funktionellen Status, „Klok-Skala“ Abb. 8), und eine Re-Evaluierung, wenn die Symptome anhaltend sind, können Verschleppung von Symptomen und eine (iatrogene) Chronifizierung verhindern (s. dazu Kap. 12).
- Kooperationspartner:innen (situationsabhängig):
  Viele Patient:innen profitieren von einer frühzeitigen Einbindung weiterer Gesundheits- und Sozialberufe wie Ergotherapie oder soziale Arbeit, da sich bei anhaltenden Symptomen soziale und existenzielle Probleme (u. a. durch Folgen auf die Teilhabe im Kontext von Erwerbsarbeit, Familie und sozialem Netzwerk) verstärken oder manifestieren können.
  Schwere oder protrahierte Verläufe erfordern jedenfalls die Beiziehung spezialisierter ärztlicher oder nicht-ärztlicher Expertise (niedergelassener Bereich, ambulante Spezialeinrichtungen, interprofessionelle Telekommunikation).

##### 9.2.1.1 Aufgaben innerhalb der hausärztlichen Versorgung im Detail

**Figure 9:**
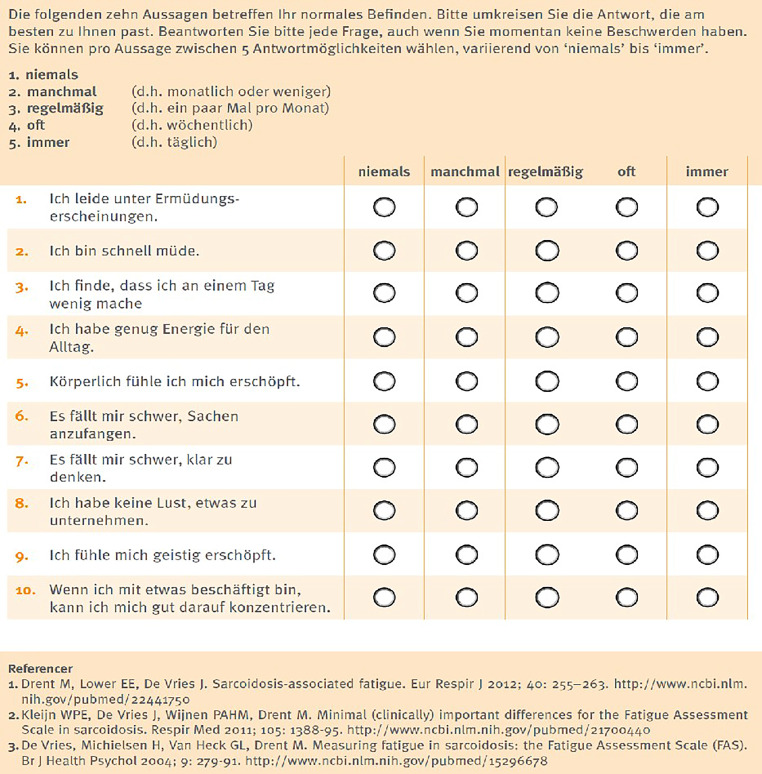

Die folgenden Maßnahmen können und sollen in der Primärversorgung durchgeführt bzw. eingeleitet werden. Sie sind in den Abschnitten Differenzialdiagnostik (Kapitel 10 und 11) bzw. Behandlung (Kapitel 12) detailliert beschrieben:

1. Ausschluss von Red Flags mit unmittelbarem Handlungsbedarf („potenziell gefährlicher Verlauf“, entsprechend dem klinischen Bild, wie üblich)
2. Ausschluss alternativer Ursachen der Beschwerden: organisch-strukturelle oder/und psychische/psychiatrische Erkrankungen (unabhängig aufgetreten, durch akute oder chronische Organschäden nach SARS-CoV-2-Infektion und/oder durch Verschlechterung/Re-Aktivierung einer Grunderkrankung)
  - Symptom-getriggerte Abklärung: Wesentlich ist die Verhinderung von Unter‑/Fehldiagnostik behandelbarer Situationen
  - Bei Bedarf in Kooperation (Überweisung) mit Fachärzt:innen, Labormedizin, radiologischer Diagnostik
  - Behandlung lt. aktuellen Leitlinien
3. Erstabklärung von Symptomen, die mit einem postviralen Zustand nach SARS-CoV-2-Infektion vereinbar sind:
  - Post-Exertional-Malaise (PEM): anhaltende, überschießende Verschlechterung der Symptome/des Zustandsbildes durch Aktivität, siehe dazu 8.9.2.1: gezielte Anamnese, Fragebogen (Differenzialdiagnostik siehe 11.1)
  - Müdigkeit/Fatigue (Grundlagen siehe 8.9; Differenzialdiagnostik siehe 11.1): nach Ausschluss alternativer Ursachen Beurteilung Schweregrad-Score, Abklärung POTS siehe unten, Monitoring
  - Autonome Dysfunktion (AD bzw. ANS; z. B. posturales Tachykardiesyndrom [POTS]) (Grundlagen siehe 8.10, Abb. 3, Differenzialdiagnostik Kreislauflabilität siehe 11.9): gezielte Anamnese und Schellong-Test (Abb. 2): Abklärung Vorhandensein sowie Verlaufsmonitoring wichtig – sowohl für Primärversorgung als auch für Spezialambulanzen
  - Symptome vereinbar mit dem Konzept einer Mastzellüberaktivierung (siehe dazu 8.8.4): gezielte Anamnese
  - Kognitive Beeinträchtigung: MMSE, Differenzialdiagnose Fatigue, PEM, POTS s. oben (siehe 11.7)
  - Weitere Symptome wie Dyspnoe, Brustschmerz, Husten, Schlafstörungen, Myalgie u. a. siehe Kapitel 11
  - Ausschluss von „Yellow Flags“: mehrere ausgeprägte Symptome (v. a. bei gleichzeitigen physischen und mentalen/sozialen Problemen), anhaltende Symptome nach schwerem Verlauf (Post-ICU), atypische Symptomatik, schwere Funktionsbehinderung (mangelnde Arbeitsfähigkeit, Alltag nicht bewältigbar, Immunkompromittierung) [302]
4. Aufklärung der Patient:innen über den meist selbstlimitierenden Verlauf der Symptome und über Möglichkeiten zum Selbstmanagement. Siehe dazu die AWMF-Patientenleitlinie [308]
5. Einleitung einer geeigneten Behandlung
  - PEM: siehe 12.1 und 12.3 (Behandlung, Pacing, Coping)
  - Orthostatische Dysfunktion: Therapieversuch mit nicht-medikamentösen Maßnahmen (siehe 12.3.3). Medikamentöse Therapieversuche empfehlen sich beim derzeitigen Stand des Wissens nur in ausgewählten Situationen (siehe dazu 8.10 und 12.1.3)
  - Symptome, die zum Konzept der Mastzellüberaktivierung passen (siehe 8.8): bei Persistenz oder deutlicher Beeinträchtigung: medikamentöse Therapieversuche „off-label“ möglich, siehe dazu 12.3.4; sorgfältiges Abwägen, gute Information der Patient:in und Dokumentation sind erforderlich
  - Symptomatische Behandlung von weiteren möglichen Symptomen, wie z. B. belastende Schmerzen, Insomnie etc. (siehe 12.3)
6. Abklärung und Monitoring der Schwere der Beeinträchtigung durch die Symptomatik bei der Bewältigung der alltäglichen Aufgaben durch die Post-COVID-19-Skala des funktionellen Status (Abb. 8), die Fatigue Assessment Scale (FAS, Abb. 9) und den PEM-Screening-Fragebogen [282] (siehe auch 10.2)
  - Objektivierung von Schweregrad und Dringlichkeit sowie für das Verlaufsmonitoring; wichtig sowohl für Primärversorgung als auch für Spezialambulanz
  - Bei Bedarf Zusammenarbeit mit anderen Gesundheits- und Sozialberufen (z. B. Ergotherapie, Sozialarbeit, psychologisch-psychotherapeutische Berufe, v. a. wenn die Symptome zu einer erheblichen Behinderung bei der Bewältigung der alltäglichen Aufgaben führen, ab Klok-Grad 2)
7. Kooperation mit Ärzt:innen und Gesundheitsberufen mit spezifischer Expertise zu postviralen Zustandsbildern bzw. spezialisierten Versorgungseinrichtungen
8. Re-Evaluierung der Symptome (Diagnostik und Therapie) nach Situation, jedenfalls nach Fortbestehen über 12 Wochen.

##### 9.2.1.2 Weiterleitung zur Abklärung möglicher alternativer Ursachen (Details in Kapitel 10 und 11)

- Unverzüglich bei Red Flags
- Elektiv und gezielt, wenn sich aus der Erstabklärung im hausärztlichen Bereich die Notwendigkeit spezialisierter Untersuchungen ergibt (entsprechend den gültigen Leitlinien)
- Bei Notwendigkeit einer Behandlung im spezialisierten Bereich inklusive Gesundheitsberufe (entsprechend den gültigen Leitlinien)

##### 9.2.1.3 Weiterleitung nach Ausschluss alternativer Ursachen und erhärtetem Verdacht auf postvirale Genese

- „Yellow Flags“ (mehrere ausgeprägte Symptome – v. a. gleichzeitige physische und mentale/soziale Probleme), anhaltende Symptome nach schwerem Verlauf (Post-ICU), atypische Symptomatik, schwere Funktionsbehinderung (mangelnde Arbeitsfähigkeit, Alltag nicht bewältigbar)
- Ausbleibende Besserung der Symptome im Verlauf von 12 Wochen nach Erkrankungsbeginn

**Die Weiterleitung in diesen Fällen soll und kann sinnvollerweise erst nach leitliniengerechter Abklärung in der hausärztlichen Primärversorgung erfolgen** [18, 302, 309–311].

##### Empfohlen als Voraussetzung für die Weiterleitung sind jedenfalls folgende Dokumente, die der Patient:in mitgegeben werden:

1. Überweisungsformular
2. Kurzer Arztbrief mit Anamnese, Symptomverlauf, aktuell vorherrschende Symptome, Komorbiditäten, Vorbefunde, Therapieversuche und Erfolge/Misserfolge, aktuelle Medikationsliste
3. Aktueller Schellong-Test (Abb. 2)
4. Aktuelle Einstufung der Beeinträchtigungsintensität bei alltäglichen Aktivitäten sowie deren Verlauf über die Wochen (FAS und Klok-Skala – Abb. 9 bzw. Abb. 8)
5. Aktueller PEM-Fragebogen [312]

##### Empfehlung

Von Beginn an soll für Beschwerden, die mit einem postviralen Zustand in Zusammenhang gebracht werden, die interprofessionelle Kooperation (ärztliche Spezialist:innen, Gesundheits- und Sozialberufe) angestrebt werden.

In der Primärversorgung sollte jedenfalls die leitliniengerechte Differenzialdiagnostik der berichteten Symptome erfolgen. Bei einer eventuellen Weiterleitung in den spezialisierten Bereich sollen Basisbefunde entsprechend den gültigen Leitlinienempfehlungen mitgegeben werden.

#### 9.2.2 Aufgaben und Kompetenzen der spezialisierten Ebene

Spezialisierte Fachpersonen sollten erst nach den in Absatz 9.2.1 beschriebenen Abklärungsschritten in Anspruch genommen werden.

Ziele:

- Gezielte weiterführende Diagnostik zum Ausschluss alternativer Ursachen: **spezialisierte Disziplinen**
- Bei postviralem Zustandsbild (z. B. autonome Dysfunktion 8.10, Fatigue 8.9, kognitive Dysfunktion 8.3.3, mit MCAS vereinbare Symptomatik 8.8.4; Indikation zur immunologischen Abklärung siehe 8.8, Small-Fiber-Neuropathie): **Ärzt:innen mit spezifischer Expertise **für postvirale Erkrankungen (wichtig ist, dass diese Expertise alle relevanten Fachbereiche umfasst, damit es nicht erneut zur Fragmentierung der Versorgung kommt) sowie Gesundheitsberufe mit spezifischer Expertise für postvirale Erkrankungen für:
  - Präzisierende Diagnostik
  - Therapievorschlag (derzeit noch hauptsächlich „off-label“) und Optimierung
  - Anbindung an Studienzentren für Therapiestudien
  - Einschätzung der Reha-Fähigkeit, ggf. Empfehlung der passenden Reha (Antrag durch Sozialarbeiter:in oder HÄ)
  - Einschätzung der Arbeitsfähigkeit
  - Kooperation mit der primärversorgenden Ebene/Gesundheits- und Sozialberufen
- Betreuungsstellen mit individualisierbarem Angebot zur Behandlung schwerer funktioneller postviraler Zustandsbilder könnten den speziellen Bedarfen gerecht werden, die mit dem tradierten Rehabilitationskonzept nicht erfasst werden können (individualisierte, personen- und kontextsensible Rehabilitation) [313]
- **Mobile Teams** für die Betreuung von immobilen Patient:innen (Unterstützung durch Videokonsultationen, Telemonitoring, digitale Gesundheitsanwendungen [DiGas])

Nach Abklärung und Therapieeinführung ist die Rückführung zur weiteren Behandlung und Koordination bei Haus- und evtl. zusätzlich einzelnen Fachärzt:innen sinnvoll.

Die Kooperation zwischen den Berufsgruppen sollte flexibel, situations- und kontextbezogen gestaltet werden können. Spezifische Expertise sollte für jede Patient:in mit geprüftem Bedarf zugänglich sein, erforderliche Berufsgruppen sind zumindest: Ärzt:innen mit Expertise hinsichtlich postviraler Erkrankung, Gesundheitsberufe wie v. a. Ergotherapie, Sozialberatung. Für Kinder sollten eigene Einrichtungen mit spezifischer Expertise aus der Kinder- und Jugendheilkunde zu Verfügung stehen.

Rückkopplungsmöglichkeiten zwischen den Behandlungspartnern können die weitere Führung der Betroffenen verbessern und zum Wissenstransfer zwischen den beteiligten Professionen beitragen.

##### Mögliche Organisationsformen

- Extra- oder intramurale ambulante Anlaufstellen für Patient:innen mit der Möglichkeit zur weiterführenden Abklärung
- Telemedizinische Services zur interprofessionellen Konsultation, evtl. in Form von Panels
- Als weitere Ebene für schwere Erscheinungsformen sollte die Einrichtung von Kompetenzzentren oder multidisziplinären Boards für postinfektiöse Erkrankungen erwogen werden, wo Forschung und Wissenschaftstransfer vorangebracht werden könnten und auch einzelne, den Bedürfnissen speziell angepasste stationäre Betten zur Verfügung stehen.

##### Empfehlung

Anlaufstellen, die über die für die Betreuung postviraler Zustände geeignete Expertise von Gesundheits- und Sozialberufen verfügen, sollen geschaffen werden. Diese sollen dann angesteuert werden, wenn die Symptomatik mit den Mitteln der primärversorgenden Ebene nicht beherrscht werden kann oder über einen Zeitraum von mehr als 12 Wochen keine Besserung erzielt wurde.

##### Empfehlung

Behandlungsziel für von postviralen Zuständen Betroffene soll die Verbesserung der Teilhabe, Aktivität und Funktion sein.

##### Empfehlung

Kompetenzzentren bzw. -netzwerke mit den Aufgaben Erforschung postviraler Zustände und Wissenstransfer sollen geschaffen werden.

## 10. Präsentiersymptome – Grundlagen der Differenzialdiagnostik

In diesem Kapitel wird der grundlegende, typisch allgemeinmedizinische Umgang mit Symptomen und Beschwerden beschrieben, der auf alle in dieser Leitlinie behandelten Symptome (siehe dazu Kapitel 6), anwendbar ist. Die Allgemein- und Familienmedizin nimmt jedes präsentierte Symptom als mehrdeutig wahr und folgt zur Abklärung und Einordnung einer logischen Struktur, die sich aus medizinisch-fachlichem Breitenwissen, Kenntnis der Patient:in und ihres Kontextes und der persönlichen und organisatorischen Kontinuität ergibt:

- Ausschluss eines potenziell gefährlichen Verlaufs (d. h. Situation mit akutem Handlungsbedarf ohne verfeinerte Diagnostik),
- Zuordnung des/der berichteten Symptoms/Symptome mittels: Anamnese, fokussierter Untersuchung, ausgewählter Zusatzuntersuchungen, Kenntnissen von Vorgeschichte und Kontext, Identifizieren von „Yellow Flags“ mit raschem Abklärungsbedarf,
- Procedereentscheidung (wie: Behandlung, Weiterleitung, abwartendes Offenhalten…).

Von Patient:innen berichtete Symptome sind grundsätzlich ernst zu nehmen. Eine Zuordnung ohne exakte Differenzierung ist unbedingt zu vermeiden, das gilt insbesondere für Symptome, die nicht oder schwer zu objektivieren sind. Die Ungewissheit, die daraus entsteht, dass postvirale Zustandsbilder derzeit noch schlecht untersucht und verstanden sind, darf nicht dazu führen, dass subjektiven Beschwerden nicht die nötige Aufmerksamkeit und Ernsthaftigkeit zuteilwird.

### Empfehlung

Alternative Ursachen für berichtete postviral aufgetretene Symptome sind mit geeigneten Mitteln auszuschließen. Nach Ausschluss eines potenziell gefährlichen Verlaufs und Ausschluss einer behandelbaren Ursache kann bei milder Symptomatik eines postviralen Zustandsbildes in vielen Fällen abwartend beobachtet werden.

### 10.1 Systematik des diagnostischen Ablaufs (Zuordnung)

Jedes der in dieser Leitlinie beschriebenen Symptome kann trotz der zeitlichen Assoziation unabhängig von COVID-19 aufgetreten sein. Unerklärte, persistierende oder ausgeprägte Symptome vor allem aus dem beschriebenen Spektrum, ohne bekannte abgelaufene Erkrankung an COVID-19 sollten dann an eine okkulte Infektion mit SARS-CoV‑2 denken lassen, wenn keine andere Ursache identifizierbar ist [18].

- Der erste diagnostische Schritt ist, wie immer, die sorgfältige **fokussierte Anamnese** mit einigen Besonderheiten:
  - Gegenwärtige Beschwerden und Symptome – exakte Exploration
    Beginn mit offenen Fragen: Welche Symptome bemerken Sie?
    Präzisierung mittels konkreter Nachfrage entsprechend den angegebenen Symptomen
    Aktives Fragen nach weiteren, nicht erwähnten Wahrnehmungen ist in Zusammenhang mit einem postviralen Zustand nach COVID-19 besonders wichtig
  - Vorbestehende Erkrankungen, Ereignisse, Medikationen und Kontextfaktoren
  - Infektionsvorgeschichte und -verlauf, insbesondere:
    Gab es Hinweise auf eine kardiale Beteiligung während der akuten Erkrankungsphase?
    Gab es Hinweise auf eine PAE/thromboembolisches Geschehen (CT, D‑Dimer)? Erfolgte eine andere Bildgebung der Lunge?
    Gab es Zeiten mit Atemnot, erheblicher Schwäche, Sauerstoffbedarf (Zeitraum und Menge erheben)
    Gab es neurologische Symptome?
    Andere Komplikationen?
    Behandlung: Was ist zu welchem Zeitpunkt geschehen?
- Danach erfolgt die fokussierte **klinische Untersuchung** entsprechend den von den Patient:innen berichteten Situationen.
- Aufgrund der Zusammenschau der Ergebnisse wird über die folgenden nötigen **Basisuntersuchungen** (Labor, apparativ etc.) und eine evtl. nötige **Weiterleitung** in den spezialisierten Bereich entschieden.

### 10.2 Diagnostische Zielsetzungen

#### 1: Ausschluss potenziell gefährlicher Verläufe – „Red Flags“ (mit oder ohne Assoziation mit COVID-19) – unmittelbarer Handlungsbedarf

Wie immer, wenn sich eine Person mit einem mehrdeutigen Symptom vorstellt, erfolgt zunächst die **Beurteilung der Dringlichkeit**. Mittels kurzer, fokussierter Anamnese und darauffolgendem **zielgerichtetem** klinischem Assessment wird ein potenziell gefährlicher Verlauf ausgeschlossen. **Alarmsignale werden nach den gleichen Prinzipien beurteilt wie immer (Vitalzeichen, Allgemeinzustand, Art und Dynamik der Symptome, zeitlicher Ablauf …).**

#### 2: Abgrenzung fassbarer Pathologien (mit evtl. behandelbarer Grundkrankheit)

Ein zeitlicher Zusammenhang muss kein kausaler Zusammenhang sein. Es muss nach COVID-19 mit allen physischen und psychischen Störungen gerechnet werden, die auch sonst möglich sind. Diese sind daher so wie üblich leitliniengerecht abzuklären bzw. auszuschließen. Zusätzlich ist auf spezielle Pathologien besonderes Augenmerk zu legen, die in besonderem Maße im Gefolge von COVID-19 auftreten, wie in Kapitel 8 beschrieben, so wird z. B. ein vermehrtes Auftreten der klassischen kardiovaskulären Erkrankungen berichtet (s. dazu 8.2).

#### 3: Evaluierung häufig mit einem Zustand nach COVID-19 assoziierter Beschwerdebilder mit Einstufung des Handlungsbedarfs

Erhebung aller zum Untersuchungszeitpunkt wahrgenommenen Symptome (offene Fragen)

Diese erfolgt im Gespräch mit der Patient:in und umfasst die Erhebung aller zum Untersuchungszeitpunkt wahrgenommenen Symptome (offene Fragen). Eine Objektivierung, auch zur vergleichenden Verlaufsbeobachtung, wird durch die Verwendung von Assessment-Skalen erleichtert.

Einen Überblick über häufig verwendete Skalen bietet Tab. 2.

**Table 2:** 

Symptomzuordnung	Diagnostik-Tool	Link
Post-Exertional-Malaise	PEM-Screening-Fragebogen	https://cfc.charite.de/fileadmin/user_upload/microsites/kompetenzzentren/cfc/Landing_Page/DSQ-PEM_TUM.pdf
Schwere der Fatigue-Symptomatik	Fatigue-Assessment-Scale	Abb. 6
Schwere der funktionellen Beeinträchtigung im Alltag	Post-COVID-19-Skala des funktionellen Status	Abb. 7
Orthostatische Dysfunktion	Schellong-Test	Abb. 8
Beurteilung Dyspnoe	BORG-Skala	Abb. 10
Beurteilung Dyspnoe	mMRC-Skala	Abb. 11

Siehe dazu auch Kap. 9, Versorgungspfad postviraler Zustand nach COVID-19.

**Hinweise auf autonome Dysfunktion, v.** **a. orthostatische Dysfunktionen**; Grundlagen siehe 8.1; Differenzialdiagnostik: Synkopen, Orthostase siehe 11.9, Benommenheit („dizziness“) siehe 11.8, Palpitationen, posturales Tachykardiesyndrom siehe 8.10.2.2, sudomotorische Störungen siehe 8.10.2.4, PEM siehe 8.9.

**Hinweise auf postvirale Müdigkeit/Fatigue:** Grundlagen siehe 8.9; Differenzialdiagnostik siehe 11.1)**: **Reduzierte Leistungsfähigkeit/übermäßige Erschöpfung (Vergleich mit Situation vor Erkrankung) **mit oder ohne PEM,** Tagesmüdigkeit ohne Besserung durch Schlaf oder Ruhepausen. Beim Zustandsbild der postviralen Fatigue sollte immer mittels validen Fragebogens erhoben werden, ob gleichzeitig eine Post-Exertional-Malaise vorliegt. Dieses Kriterium ist für die weitere Therapieempfehlung, vor allem in Bezug auf mögliche Aktivierung, von großer Bedeutung. Zum Assessment: **PEM-Screening-Fragebogen** für Erwachsene und für Kinder und Jugendliche s. Tab. 2.

**Hinweise auf Symptome, die in Verdacht stehen, mit dem Konzept einer MCAS (allergieartige Symptome) vereinbar zu sein** (Mastzellaktivierungssyndrom; Grundlagen siehe 8.8): Haut (Urtikaria, Flush, Pruritus und/oder Angioödem), Respirationstrakt (verstopfte Nase, nasaler Pruritus, keuchende Atmung, Schwellungen im Halsbereich und/oder Heiserkeit), kardiovaskuläres System (Kopfschmerzen, hypotensive Synkopen und/oder Tachykardie), Gastrointestinaltrakt (abdominale Krämpfe, Diarrhö und/oder Übelkeit).

**Hinweise auf kognitive Dysfunktion (Grundlagen** siehe 8.3**): **Vergesslichkeit, Konzentrationsschwierigkeiten sowie Aufmerksamkeitsstörungen, „Brain Fog“.

**Hinweise auf psychosoziale Belastungen** in Zusammenhang mit persistierenden postviralen Zustandsbildern dürfen nicht übersehen werden.

**Vor allem wenn Einzelsymptome der beschriebenen Beschwerdekomplexe erhoben werden, ist die Differenzialdiagnostik zum Ausschluss alternativer Ursachen, wie in **Kap. 11** beschrieben, unerlässlich.**

#### Empfehlung

Die allgemeinmedizinische Zuordnung von Symptomen soll gezielt und unter Einsatz der fachspezifischen Mittel wie erlebte und erhobene Anamnese mit Kontextfaktoren, fokussierter klinischer Untersuchung und gezieltem Einsatz weiterer Mittel erfolgen.

#### Empfehlung

Die Dringlichkeitsbeurteilung umfasst die Identifikation von Situationen mit unmittelbarem Handlungsbedarf, solchen mit raschem Abklärungsbedarf und Situationen, die die elektive Abklärung oder ein Abwarten unter Beobachtung ermöglichen.

## 11. Symptombezogene Abklärungsgänge: häufige Präsentiersymptome

### 11.1 Erschöpfung (Müdigkeit/Fatigue/Leistungsminderung/Schwäche)

#### 11.1.1 Allgemeines

Müdigkeit und Erschöpfung in der Rekonvaleszenz nach viralen Infektionen auch über mehrere Wochen sind nicht selten. Diese sind abzugrenzen von länger anhaltenden Erschöpfungszuständen bis hin zur Fatigue nach spezifischen viralen Infektionen wie nach COVID-19, Influenza, Parvoviren oder EBV-Infektion, die über „normale“ Rekonvaleszenzsymptome hinausgeht (s. dazu Abschn. 8.9).

Organisch-strukturelle Ursachen in Zusammenhang mit abgelaufener COVID-19 bzw. Erkrankungen ohne Zusammenhang mit einer solchen müssen ausgeschlossen werden, Zusatzsymptome, die das Management einer Fatigue beeinflussen, müssen erfasst werden.

Siehe dazu auch Leitlinie S3 der DEGAM: [12].

#### 11.1.2 Ausschluss potenziell gefährlicher Verlauf („Red Flags“)

- Vitalparameter, klinischer Gesamteindruck
- Weitere Red Flags, z. B.: Zeichen einer Blutung, Intoxikation (Substanzgebrauch, Medikamente), Hypoglykämie, Stauungszeichen, Dyspnoe
- Erfassen der subjektiven und objektiven Beeinträchtigung (S_p_O_2_, Belastungstests, Scores)

#### 11.1.3 Zuordnung

##### Anamnese

- Hinweise im akuten/protrahierten Verlauf der COVID-19-Erkrankung auf organspezifische Schädigungen (Myokarditis, KHK, PE …)
- Bestehende chronische organische oder Erkrankungen inklusive psychische Erkrankungen
- Psychosoziale Belastungsfaktoren
- Substanzgebrauch, Medikamente?
- (Vorangegangene) Blutungen oder Stürze
- Atemnot, Belastungsdyspnoe
- Gewichtsab-/-zunahme, Ödeme
- Fieber, Nachtschweiß
- Schmerzanamnese
- Ess‑/Trinkverhalten wegen möglichen Vitaminmangels/Elektrolytverschiebungen
- Stuhl‑/Harnverhalten
- Schlafverhalten und Schlafqualität
- Anamnese bestehender Zusatzsymptome wie orthostatische Dysfunktionen, PEM, Symptome, die mit dem Konzept einer Mastzellüberaktivierung in Einklang zu bringen sind (s. oben)

##### Klinische Untersuchung

- Fokussierter Status: körperliche Untersuchung: Auskultation und Perkussion Cor, Pulmo, Abdomen, Hautkolorit, Schleimhäute, Palpation Abdomen, Lymphregionen, Schilddrüse, Stauungszeichen, Ödeme
- Orientierende neurologische Beurteilung
- Bei fehlenden Hinweisen auf erklärende Ursachen:
  - Beurteilung Vorhandensein POTS: Schellong-Test (Abb. 2), Weiteres s. Abschn. 8.10.2
  - Evaluierung PEM, wenn keine Hinweise auf eine ursächliche Pathologie gefunden wurden (s. weiter oben, sowie Kapitel Fatigue Abschn. 8.9) mittels gezielter Anamnese und dem **PEM-Screening-Fragebogen **[282]: validierter PEM-Fragebogen (DePaul-Symptom-Questionnaire) für Erwachsene und für Kinder und Jugendliche: Dieses Vorgehen ist von großer Wichtigkeit, da sich die gesamte weitere Empfehlung und Behandlung hinsichtlich Aktivierung danach richten müssen [282].
    Patient:innen mit positivem PEM-Screening dürfen keine Aktivierungstherapie erhalten, sondern müssen sich streng an die Therapieform des Pacing halten. s. dazu Abschn. 12.2.1.
    Wenn keine PEM vorliegt, sollen die Patient:innen nach der Erkrankung symptomgetriggert wieder aktiviert werden, was vor allem bei Störungen des ANS von großer Bedeutung ist (s. Behandlung, Abschn. 12.2.3).
    Objektivierung mittels Scores und Fragebögen von Vorhandensein, Schweregrad sowie für das Verlaufsmonitoring ist von größter Wichtigkeit; wichtig für Maßnahmen/Therapien sowohl für PV als auch für Spezialambulanz
    Fatigue Assessment Scale (FAS): (Abb. 9)
    Klok-Assessment-Scale zur Einschätzung des funktionellen Status im Alltag: (Abb. 8)

##### Basisdiagnostik

- Basisdiagnostik: RR, Puls, S_p_O_2_, Körpertemperatur, ggf. EKG, BZ, BB, CRP
- Je nach Leitsymptomen, Anamnese und körperlicher Untersuchung zusätzlich: TSH, Vitamin D, Vitamin B_12_, Folsäure, CK, CK-MB, TropT, proBNP, B‑Vitamine, Eisenstatus, Nieren- und Leberwerte, Elektrolyte, Stuhlprobe auf Blut

##### Yellow Flags

- Mehrere ausgeprägte Symptome – v. a. gleichzeitige physische und mentale/soziale Probleme
- Anhaltende Symptome nach schwerem Verlauf (Post-ICU)
- Hinweise auf strukturelle Erkrankungen
- Atypische Symptomatik, schwere Funktionsbehinderung (mangelnde Arbeitsfähigkeit, Alltag nicht bewältigbar: Klok > 2, s. unten)

##### Weiterführende Untersuchungen

Bei gegebenem Anlass und entsprechenden Symptomen, bei Vorliegen von „Yellow Flags“. Sonst erst bei Re-Evaluierung bei Persistenz über mehr als 3 Monate:

- Kardiologische Untersuchung (Ergo, Echo): Herzinsuffizienz, KHK, pulmonalarterielle Hypertension, Arrhythmien. Bei Zustand nach SARS-CoV-2-Infektion ist u. a. auch an entzündliche oder postentzündliche Ätiologien zu denken (s. Abschn. 8.2)
- Neurologische Untersuchung, v. a. bei zusätzlichen kognitiven Dysfunktionen (MRT), neurogenen Schmerzen/autonomen Dysfunktionen (Abschn.8.3 und 8.10)
- Pulmonologische Begutachtung: (atypische) Fibrose, Tumor, PE, COPD, Asthma, Schlafapnoe etc. – s. Abschn. 8.1
- Rheumatologische Begutachtung bei zusätzlichen neurogenen, Muskel- und Gelenksschmerzen, evtl. Hinzuziehen von Schmerzambulanzen
- Weitere internistische Begutachtung (gastroenterologisch, nephrologisch, hepatologisch, endokrinologisch, onkologisch etc.)

#### 11.1.4 Procedere

##### Abwartendes Offenhalten

Wenn eine erste Abklärung inklusive Labor keine Hinweise auf einen abwendbar gefährlichen Verlauf zeigt („Red Flags“) und keine weiteren anamnestischen und klinischen Anzeichen für strukturelle Erkrankungen gefunden werden und keine weiteren „Yellow Flags“, wie z. B. eine PEM, vorliegen (s. weiter oben), kann in Absprache mit der Patient:in mit einer weiterführenden Abklärung zugewartet werden.

- Die Prognose ist in den meisten Fällen gut. Wesentlich ist die gute Information der Betroffenen
- Klinische Kontrolle bei der Hausärztin/dem Hausarzt je nach Situation, zumindest nach 4 bis 6 Wochen [314].
  Auf die Notwendigkeit der Wiedervorstellung bei Verschlechterung ist dokumentiert hinzuweisen!

##### Fachspezifische Abklärung

- Hinweise auf kardiale Ursachen: Abklärung nach etablierten Routinen: Herzinsuffizienz, KHK, pulmonalarterielle Hypertension, Arrhythmien. Bei Zustand nach COVID-19 ist u. a. an entzündliche oder postentzündliche Ätiologien zu denken (s. dazu 8.2).
- Hinweise auf pulmonale Ursachen (siehe 8.1): Abklärung nach etablierten Routinen.
- Hinweise auf andere relevante Erkrankungen: weitere Abklärung nach jeweiligen Leitlinien.
- Postvirale Ätiologie: bei Persistenz > 3 Monate oder erheblichem Leidensdruck: Begutachtung durch spezialisierte Einrichtung mit Erstellen eines Therapieplans (s. Kapitel 9).
- Patient:innen über die zu erwartende Selbstlimitierung der Symptome aufklären: Nach viralen Erkrankungen sind postvirale Erschöpfungszustände im Rahmen der Rekonvaleszenz häufig und vor allem häufig selbstlimitierend.

#### 11.1.5 Wichtige Differenzialdiagnosen ohne postinfektiöse Ätiologie

- Kardiale oder pulmonale Erkrankungen
- Nierenerkrankungen
- Lebererkrankungen
- Rheumatologische Erkrankungen
- Gastroenterologische Erkrankungen (z. B. CED, Blutungen …)
- Onkologische Erkrankungen
- Anämie und Blutungen
- Schilddrüsenfunktionsstörungen
- Andere hormonelle Dysfunktionen
- Chronische Infektionen/Entzündungen anderer Genese
- Stoffwechselerkrankungen (z. B. Diabetes)
- Muskuläre Schwäche
- Überlastungssituationen
- Psychische Erkrankungen (z. B. Depression, Angststörungen)
- Gestörter Schlaf und/oder Schlafstörungen

**Behandlung**: siehe Kapitel 12 (12.2, 12.3.2)

##### Empfehlung

Beim Zustandsbild der postviralen Müdigkeit/Erschöpfung sollte schon zu Beginn mittels sorgfältiger Anamnese und validen Fragebogens (PEM-Screening-Fragebogen für Erwachsene und für Kinder und Jugendliche) eine Situationsbeurteilung stattfinden.*

* Das Vorliegen von PEM darf aus Sicht der Neurologie und Psychiatrie einer leitliniengerechten Therapie neurologischer und psychiatrischer Erkrankungen nicht im Wege stehen.

##### Empfehlung

Bei geringer bis mäßiger Beeinträchtigung aufgrund postinfektiöser Müdigkeit und Fehlen von Red Flags sollte eine weiterführende Diagnostik nur bei anhaltenden Beschwerden über 12 Wochen angestrebt werden. Eine Überdiagnostik ist zu vermeiden, um eine iatrogene Fixierung hintanzuhalten.

### 11.2 Riech- und Schmeckstörungen

#### 11.2.1 Allgemeines

Einer Riechstörung können 2 pathophysiologische Mechanismen zugrunde liegen: Blockade des Duftstofftransports zur Riechschleimhaut im Nasendach oder eine sensorineurale Schädigung (Riechnervenzellen oder zentrale Strukturen wie Bulbus olfactorius oder Hirnareale).

Die COVID-bedingte Riechstörung fällt in die zweite Gruppe, in die auch vor Beginn der COVID-19-Pandemie andere Viren (z. B. Influenza‑, Parainfluenza‑, Rhinoviren) unter dem Begriff der postinfektiösen bzw. postviralen Riechstörung zusammengefasst wurden [184].

#### 11.2.2 Ausschluss potenziell gefährlicher Verlauf

Einige Hirntumoren können mit Riechverlust einhergehen (Meningeome, Tumoren der vorderen Schädelgrube), auch Insulte. Dabei stellt der Riechverlust normalerweise kein Einzelsymptom dar.

#### 11.2.3 Zuordnung

##### Anamnese

- Zeitpunkt des Auftretens, Verlauf, genaue Symptomatik erfragen: Hyposmie, Dysosmie, Trauma, Chemo- oder Strahlentherapie, Operation? Weitere Symptome (neurologisch oder internistisch)?

##### Klinische Untersuchung

- Riechtest
- Hinweise auf sinunasale Erkrankungen? Gezielte neurologische Untersuchung

#### 11.2.4 Procedere

Wenn es einen klaren zeitlichen Zusammenhang mit der SARS-CoV-2-Infektion gibt, und Hinweise auf andere Erkrankungen fehlen, kann ohne weiterführende Untersuchung von einer COVID-19-assoziierten Riechstörung ausgegangen werden.

#### 11.2.5 Weitere wichtige Differenzialdiagnosen ohne postinfektiöse Ätiologie

- Sinunasale Erkrankungen (z. B. chronische Sinusitis, Allergie, Septumdeviation, Rhinopathia gravidarum, Tumoren)
- Schädel-Hirn-Trauma
- Andere Infekte (nasal oder systemisch, z. B. Influenza, „common cold“)
- Zentrale Ursachen (z. B. Meningeom, Insult)
- Neurodegenerative Erkrankungen (z. B. Morbus Parkinson, Morbus Alzheimer)
- Internistische Erkrankungen (z. B. Leber‑, Nieren‑, Schilddrüsenerkrankungen)
- Medikamentös-toxische Einflüsse (z. B. Chemo‑, Strahlentherapie)
- Iatrogen (neurochirurgische Operation, Nasen/NNH-Operationen)
- Angeborene Riechstörung (z. B. Kallmann-Syndrom)
- Idiopathische Riechstörung (Ausschlussdiagnose!)

**Behandlung** siehe 12.3.6.

Riechtraining: Abb. 3

### 11.3 Kopfschmerz

#### 11.3.1 Allgemeines

Wie bei anderen systemischen Infektionen ist Kopfschmerz auch ein häufiges Symptom einer akuten COVID-19-Erkrankung. In einer spanischen Studie waren bei 106 Patient:innen in 94,6 % die ICHD-3-Kriterien eines „sekundären Kopfschmerzes zurückzuführen auf eine systemische Infektion“ erfüllt [197].

Sekundäre Kopfschmerzen als Symptom einer Komplikation der COVID-19-Erkrankung (Sinus‑/Hirnvenenthrombose, intrakranielle Hypertension, Meningitis) sind dagegen seltener. Eine weitere Abklärung muss beim Vorliegen von Hinweisen auf eine zugrunde liegende Pathologie veranlasst werden [162]. In 6–45 % persistieren Kopfschmerzen über die akute Phase von COVID-19 hinaus.

Dieser Kopfschmerz tritt typischerweise bilateral auf, ist von mäßiger bis starker Intensität und hat häufig Charakteristika eines Kopfschmerzes vom Spannungstyp oder auch eines Migränekopfschmerzes mit den Begleitsymptomen Lärm‑, Lichtempfindlichkeit, Übelkeit und Erbrechen. In 61 % bestehen diese Kopfschmerzen täglich als Dauerkopfschmerz und imponieren als neu aufgetretener täglicher Kopfschmerz („new daily persistent headache“ [NDPH]), aber auch eine Verschlechterung vorbestehender primärer Kopfschmerzen ist möglich [164, 165].

#### 11.3.2 Ausschluss eines potenziell gefährlichen Verlaufs

##### Sofortiger Handlungsbedarf (potenzieller Notfall)

Neurologische Symptome, plötzliches oder abruptes Erstauftreten bisher unbekannter, heftiger Kopfschmerzen, Papillenödem, schmerzhaftes Auge mit autonomen Symptomen, posttraumatisches Erstauftreten, Fieber und reduzierter Allgemeinzustand.

**Weitere Red Flags,** die eine rasche Abklärung erfordern, s. dazu Do TP et al. [315]:

Alter > 65 Jahre bei Erstauftreten, systemische Symptome, Symptomwechsel bekannter Kopfschmerzen oder Erstauftreten unbekannter Kopfschmerzen hinsichtlich Charakteristik/Intensität/Frequenz, lageabhängiger Kopfschmerz, Kopfschmerz nach Schnäuzen/Husten/körperlichem Training, progredienter Kopfschmerz und atypische Präsentation, Schwangerschaft oder Wochenbett, Beeinträchtigung des Immunsystems wie HIV, Schmerzmittelübergebrauch oder neue Medikation bei Erstauftreten der Kopfschmerzen, Tumoranamnese.

#### 11.3.3 Zuordnung

##### Anamnese

Kopfschmerzanamnese hinsichtlich vorbekannter Kopfschmerzen bzw. Erstauftreten, Dauer, Lokalisation, Charakteristik, Intensität, Häufigkeit, Begleitsymptome, Medikationsanamnese, Alter, Lage- oder Belastungsabhängigkeit, Schwangerschaft, Kontrazeption, Wochenbett.

##### Klinische Untersuchung

Fokussierte körperliche und neurologische Untersuchung, Blutdruckmessung.

##### Weitere Untersuchungen

Bei unauffälliger klinisch-neurologischer Untersuchung, fehlenden Red Flags und fehlenden Hinweisen auf eine organische Erkrankung vorerst keine weitere Diagnostik. Bei Auffälligkeiten MRT-Bildgebung einschließlich Gefäßdarstellung und neurologische Abklärung.

#### 11.3.4 Procedere

##### Abwartendes Offenhalten

Bei einem Großteil der Patient:innen bessern sich die Kopfschmerzen innerhalb der ersten 3 Monate nach der Akutinfektion [162].

Wenn die erste Abklärung mittels Anamnese und neurologischer Untersuchung keinen Hinweis auf eine strukturelle Erkrankung ergibt (Fehlen von Red Flags bzw. Fehlen von Hinweisen auf organische Erkrankung), ist eine weiterführende Abklärung daher vorerst nicht notwendig.

##### Fachspezifische Untersuchung

Bei Auffälligkeiten in Anamnese und/oder klinischer Untersuchung leitliniengerechte Abklärung je nach Situation.

Bei Persistenz länger als 3 Monate ist jedenfalls eine fachärztliche Untersuchung durch Neurologen zu empfehlen.

#### 11.3.5 Wichtige Differenzialdiagnosen ohne postinfektiöse Ätiologie

Akuter Kopfschmerz:

- Migräneattacke ohne Assoziation mit COVID-19
- Cluster-Kopfschmerz
- Plötzlicher Blutdruckanstieg
- Kopfschmerz bei körperlicher Anstrengung, koitale Kopfschmerzen
- Vasodilatanzien, Nitrate
- Neuralgien
- Subarachnoidalblutung (SAB) und intrazerebrale Blutungen
- Meningitis oder Enzephalitis
- Schädel-Hirn-Trauma
- Erhöhter intrakranieller Druck
- Phäochromozytom
- Glaukomanfall (akutes Engwinkelglaukom)

Länger bestehender Kopfschmerz:

- Bekannter anfallsartiger Kopfschmerz (Migräne, Cluster …)
- Spannungskopfschmerzen
- Sinusitis, Otitis
- Kopfschmerzen, verursacht durch Erkrankungen der Zähne bzw. durch Bissanomalien
- Augenerkrankungen (z. B. Refraktionsfehler)
- Übergebrauch von Analgetika
- Posttraumatische Kopfschmerzen
- Intrakranielle Hypotension
- Tumoren
- Chronische Meningitis (Sarkoidose, Pilze, Tuberkulose)
- Hyperthyreose
- Hyperparathyreoidismus
- Hypoglykämie, Hypoxie, Hyperkapnie
- Vaskulitis, Sinus‑/Hirnvenenthrombose
- Chronische tägliche Kopfschmerzen: tägliche oder beinahe tägliche Kopfschmerzen, die eine Kombination unterschiedlicher Kopfschmerzarten darstellen können, wie etwa chronische Migräne, chronische Spannungskopfschmerzen, Medikamentenübergebrauchskopfschmerz etc.

**Behandlung**: Für den Kopfschmerz nach COVID-19 ist keine kausale Therapie bekannt, die symptomatische adäquate Therapie der Schmerzen steht im Vordergrund. Die Behandlung erfolgt entsprechend der vorherrschenden Kopfschmerzcharakteristik (z. B. Spannungstyp oder Migräne) analog der jeweiligen Empfehlungen. Siehe auch 12.1.

### 11.4 Dyspnoe

#### 11.4.1 Ausschluss potenziell gefährlicher Verlauf

- Erfassung der subjektiven und objektiven Beeinträchtigung (Pulsoxymetrie, Belastungstests). Bei subjektiver ausgeprägter Atemnot und/oder S_p_O_2_-Werten < 93 % (bei vorbestehender chronischer respiratorischer Erkrankung: deutlicher Abfall) besteht akuter Handlungsbedarf [19].
- Weitere „Red Flags“: rezentes (neues) Auftreten; plötzliche oder rasche Zunahme, zusätzliche Symptome (Thoraxschmerz, Husten, Hämoptysen, Fieber, Stauungszeichen, Hautkolorit, EKG-Veränderungen, kognitive Veränderungen).

#### 11.4.2 Zuordnung

##### Anamnese (Zeitpunkt des Auftretens, Dynamik, Belastungsabhängigkeit)

**Figure 10:**
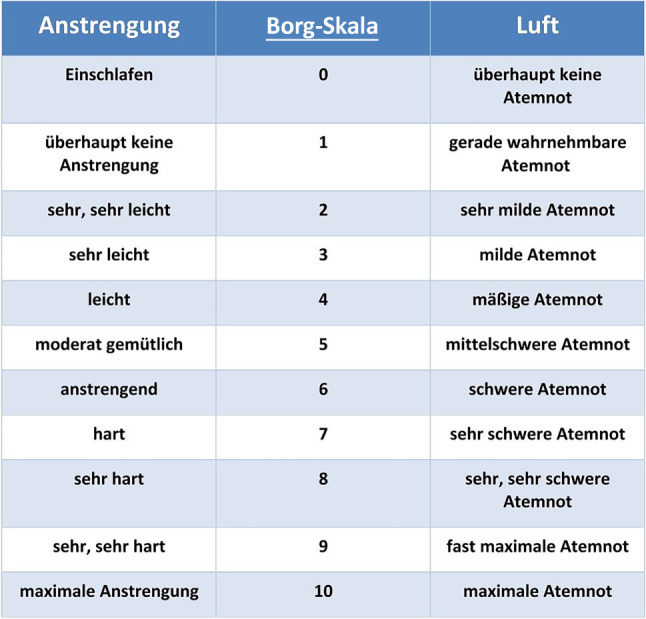

**Figure 11:**
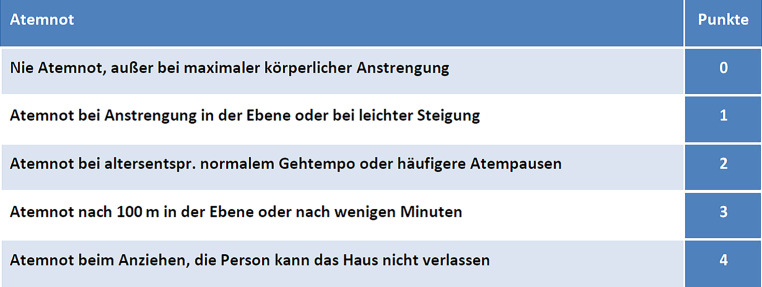

- Dyspnoe (nur) in Ruhe, bei Belastung, oder Bewegung (wie Vorwärtsneigen)?
- Tatsächlich Atemnot – oder mangelnde Leistungsfähigkeit, Müdigkeit bis Fatigue? Belastungsintoleranz ohne objektivierbare Dyspnoe?
- Postinfektiöse Dyspnoe äußert sich vor allem als Kurzatmigkeit bei Belastung und findet sich häufiger nach schwerem Verlauf, aber auch nach nicht-hospitalisiertem Verlauf (in ca. 10 %) [316]
- Frage nach Vorerkrankungen und weiteren Symptomen
- Einstufung Dyspnoe-Ausmaß: nach NYHA-Klassifikation, Borg-Skala (Abb. 10), mMRC (Abb. 11)

##### Klinische Untersuchung

- Fokussierte Basisuntersuchung: Auskultation Herz und Lunge, Suche nach Stauungszeichen, Hautkolorit, Beurteilung der Atmung mit Objektivierung der Dyspnoe: Atemfrequenz, Sprechen, Belastungstests unter Beobachtung (Gehtest, Stiegensteigen) und Einstufung nach NYHA, Abgrenzung muskulärer Schwäche (Abb. 11)

##### Basisdiagnostik

- Jedenfalls: S_p_O_2_ (auch im Selbstmonitoring!), RR, KBB, CRP

##### Weiterführende Untersuchungen

- Bei klinisch objektivierbarer Dyspnoe: Thoraxröntgen, EKG, NT-proBNP. Fehlende Hinweise auf kardiale Genese: Spirometrie, Diffusionsmessung, evtl. D‑Dimer bei entsprechenden Verdachtsmomenten bzw. Risikofaktoren (Schwangerschaft, „Pille“).

#### 11.4.3 Procedere

##### Abwartendes Offenhalten

Wenn eine erste Abklärung keine Hinweise auf reduzierte Leistungsfähigkeit oder sonstige deutlich Beeinträchtigungen erbringt und keine weiteren anamnestischen und klinischen Anzeichen für strukturelle Erkrankungen gefunden werden, kann in Absprache mit der/dem Betroffenen mit einer weiterführenden Abklärung zugewartet werden (Fehlen von Red Flags).

- Bis zu ca. 6 Wochen nach ambulanter Erkrankung ist eine milde Kurzatmigkeit häufig. Sollte diese anamnestisch bereits gebessert und mild sein, sind bei unauffälligem klinischem Status, unauffälligem Routinelabor und unauffälligem Thoraxröntgen vorerst keine weiterführenden Untersuchungen erforderlich.
- Klinische Kontrolle beim Hausarzt je nach Situation, zumindest nach 4 bis 6 Wochen [314].
  Auf die Notwendigkeit der Wiedervorstellung bei Verschlechterung ist dokumentiert hinzuweisen!

##### Fachspezifische Abklärung

- Hinweise auf kardiale Ursachen: Abklärung nach etablierten Routinen.
  Herzinsuffizienz, KHK, pulmonalarterielle Hypertension, Arrhythmien. Bei Zustand nach COVID-19 ist z. B. auch an entzündliche oder postentzündliche Ätiologie zu denken, s. Kapitel 7.
- Hinweise auf pulmonale Ursachen: Abklärung nach etablierten Routinen.
  Zustand nach COVID-19: bei Risikofaktoren oder klinischen Hinweisen (akutes Einsetzen, akute Verschlechterung) Ausschluss einer PE (D-Dimer).
  - Bei pathologischem Thoraxröntgen sowie ausgeprägter Dyspnoe mit Beeinträchtigung der Leistungsfähigkeit oder bei Verschlechterungstendenz ist die fachärztliche pneumonologische Abklärung erforderlich, um strukturelle Lungenschäden nach COVID-19 zu definieren wie residuale Pneumonie, organisierende Pneumonie, Hinweise für Fibrose, chronische PE – siehe dazu 8.1.

#### 11.4.4 Weitere wichtige Differenzialdiagnosen ohne postinfektiöse Ätiologie

Muskuläre Dekonditionierung z. B. nach einer protrahierten Erkrankung, Anämie, Adipositas, Skeletterkrankungen, neuromuskuläre Erkrankungen.

**Behandlung** siehe 12.2 und 12.3.1.

### 11.5 Husten

#### 11.5.1 Allgemeines

Husten ist bei einer akuten COVID-19-Erkrankung ein häufiges Symptom und bleibt nach durchgemachter Erkrankung bei vielen Patient:innen über Wochen bestehen, so wie bei vielen anderen respiratorischen Infektionen.

Bis 8 Wochen nach Beschwerdebeginn ist von einem postviralen Husten auszugehen, wo außer Anamnese und Status keine weitere Diagnostik notwendig ist. Voraussetzung ist das Fehlen von Warnzeichen.

Wenn aber der Husten über 8 Wochen hinausgeht, handelt es sich um einen chronischen Husten – dieser muss weiter abgeklärt werden (s. dazu Leitlinie der DEGAM [317]). Dies gilt auch nach COVID-19.

Entscheidend ist die Frage, ob es auch beschwerdefreie Episoden gibt, dann handelt es sich eher um rezidivierend auftretenden Husten, z. B. im Rahmen rasch aufeinanderfolgender Atemwegsinfektionen und eher nicht um Husten aufgrund einer SARS-CoV-2-Infektion.

#### 11.5.2 Ausschluss potenziell gefährlicher Verlauf

Kombination mit Dyspnoe, Belastungsassoziation, Hämoptysen, Thoraxschmerz, Stauungszeichen.

#### 11.5.3 Zuordnung

##### Anamnese

- Beginn, Dauer und Art des Hustens, Vorgeschichte (Hinweise auf Asthma/COPD, andere vorbestehende Erkrankungen), weitere Symptome, Auswirkungen auf Leistungsfähigkeit und Nachtschlaf
  - Zunehmender Husten nach Virusinfektion, vor allem in Verbindung mit Verschlechterung des Allgemeinzustandes, Dyspnoe und Hypoxie, lässt an eine bakterielle Superinfektion denken.

##### Klinische Untersuchung

- Auskultation von Herz und Lunge, Beurteilung des Oberbauchs (Reflux), s. dazu DEGAM-Leitlinie Husten [317].

##### Weitere Untersuchungen

- Labor: BB, CRP bei Verdacht auf bakterielle Infektion
- Weitere Basisuntersuchungen: Lungenfunktion mit ggf. Broncholyse, Thoraxröntgen, evtl. D‑Dimer, weitere Bildgebung (bei Verdacht auf Pulmonalembolie – klinisch bzw. bei Vorliegen zusätzlicher Risikofaktoren)

#### 11.5.4 Procedere

##### Abwartendes Offenhalten

Wenn keine Hinweise auf eine organisch-strukturelle Erkrankung gefunden werden, und die Beeinträchtigung nur mäßig ist, kann zugewartet werden (Grenze: 8 Wochen). Regelmäßige Kontrollen und die Wiedervorstellung bei Verschlechterung oder zusätzlicher Symptomatik werden vereinbart und dokumentiert.

##### Fachspezifische Abklärung

Bei belastender Symptomatik, die mit den angeführten Mitteln nicht erklärt und behandelt werden kann, erfolgt die Zuweisung an die entsprechend den Ergebnissen aus Anamnese und Untersuchung geeignete Stelle.

#### 11.5.5 Weitere wichtige Differenzialdiagnosen ohne postinfektiöse Ätiologie

Alle internationalen Leitlinien empfehlen nach einer Hustendauer von 8 Wochen die Abklärung auf Neoplasien, rez. Lungenembolien, Fremdkörperaspiration und chronische Linksherzinsuffizienz mit Lungenstauung.

##### Empfehlung

Unerklärter oder therapieresistenter Husten sollte jedenfalls nach einer Beschwerdedauer von mehr als 8 Wochen fachspezifisch abgeklärt werden. Ein Versuch mit Atemtraining kann empfohlen werden.

**Behandlung** siehe 12.4.3.

### 11.6 Thorakale Beschwerden

#### 11.6.1 Allgemeines

Thorakale Beschwerden treten noch Wochen nach akuter Infektion häufig auf. Bei thorakaler Post-COVID-Symptomatik sind die Abklärung kardialer und pulmologischer Ursachen von Relevanz im Hinblick auf die häufige Beteiligung dieser Organsysteme.

Pulmonale Genese: wenn keine organische Ursache gefunden wird, bestehen möglicherweise eine autonome Dysfunktion und Muskelschwäche im Rahmen des postviralen Zustandsbildes. Beispielsweise gibt es bei physiotherapeutischen Untersuchungen Hinweise für eine Einschränkung der Zwerchfellmobilität sowie Hinweise auf eine Muskelschwäche der Atemmuskulatur (siehe dazu 8.1, Pulmonologie).

Kardiale Genese: Eine kardiale Beteiligung ist bei anhaltenden Beschwerden nach COVID nicht selten (siehe dazu 8.2, Kardiologie). Bei der Abklärung steht die Detektion einer evtl. bestehenden Ischämie oder entzündlicher Herzerkrankung im Vordergrund.

Daneben muss mit allen anderen bekannten Ursachen für thorakale Beschwerden gerechnet werden. Auch hier gilt, dass der zeitliche Zusammenhang mit einer SARS-CoV-2-Infektion den Blick nicht trüben darf.

#### 11.6.2 Ausschluss potenziell gefährlicher Verlauf

##### Hinweise für instabile Situation? (Z. B.: akutes Koronarsyndrom, Pulmonalembolie …)

- Zeichen des akuten Kreislaufversagens (Schockindex > 1)
- (Unmittelbar vorangegangene) Synkope oder Kollaps
- Kaltschweißigkeit
- Aktuelle Ruhedyspnoe (Vorgehen s. Abschn. 9.4)
- Ausgeprägte Angst der/des Betroffenen
- Tachykardie

##### Weitere „Red Flags“

- Fieber, starke Schmerzen, akut beeinträchtigte Atmung
- Symptomatik vereinbar mit instabiler Angina pectoris
- Zeichen einer dekompensierten Herzinsuffizienz (Dyspnoe, Ödeme)

#### 11.6.3 Zuordnung

##### Anamnese

- Schmerzcharakteristik, Zeitpunkt des Auftretens, Dynamik, Atemabhängigkeit, Belastungsabhängigkeit, Bewegungsabhängigkeit, Leistungsminderung, Dyspnoe, Dyspepsie, Abhängigkeit von der Nahrungsaufnahme. Frage nach Verlauf der Akuterkrankung (Hinweise auf kardiale oder pulmonale Beteiligung?)

##### Klinische Untersuchung

- Fokussierte Basisuntersuchung: Inspektion, Auskultation Herz und Lunge, Palpation des Thorax (Rippen, WS), dynamische Untersuchung des Oberkörpers und der oberen Extremitäten, Hautkolorit, Palpation des Oberbauchs

##### Weitere Untersuchungen

- RR, 12-Kanal-EKG (wenn kardiale Ursache klinisch nicht ausschließbar), Belastungstests, Thoraxröntgen (wenn pulmonale Ursache nicht ausschließbar). D‑Dimer, CRP, BB nur bei entsprechenden Hinweisen und Verdachtsmomenten.

#### 11.6.4 Procedere

##### Abwartendes Offenhalten

Nach sicherem Ausschluss von Red Flags kann bei gutem klinischem Zustandsbild und fehlenden Hinweisen für reversible organspezifische Ursachen eine symptomatische Therapie entsprechend der vermuteten Pathogenese etabliert werden.

##### Fachspezifische Abklärung

Hinweise auf kardiale Ursachen:

- Abklärung nach etablierten Routinen (s. Abschn.8.2). Besonders zu beachten: Myokardischämie (EKG), entzündliche Herzerkrankungen

Hinweise auf pulmonale Ursachen:

- Abklärung nach etablierten Routinen (s. Abschn.8.1) – insbesondere bei gemeinsamem Auftreten mit Dyspnoe/Tachypnoe (siehe dazu 11.4):
- Akute Lungenembolie (D-Dimer), Pneumothorax, Pleuritis

Hinweise auf muskuloskeletale Ursachen:

- Bewegungsmangel (Isolierung, Kontaktreduktionsmaßnahmen)
- Patient:innen nach schwerem Verlauf (Hospitalisierung): Folge von Muskelabbau durch Immobilisierung

#### 11.6.5 Wichtige Differenzialdiagnosen ohne postinfektiöse Ätiologie

Thoraxschmerzen können sehr häufig durch eine Reihe muskuloskeletaler Probleme bedingt sein, ebenso finden sich gastrointestinale (Reflux, Zwerchfellhernie, Ulkus …) oder psychogene Ursachen. Kombinationen sind, wie immer, möglich. Empfehlungen und Leitlinien zur Differenzialdiagnostik des Thoraxschmerzes, s. dazu die gültigen Leitlinien wie die der DEGAM [318].

**Behandlung** siehe 12.3.1.

### 11.7 Störungen der Hirnleistung

#### 11.7.1 Allgemeines

Es kann sich hierbei um Probleme in folgenden Bereichen handeln:

- Aufmerksamkeit: „Brain fog“, Gedankenabrisse, Konzentrationsstörungen,
- Exekutivfunktionen: vorausschauendes Denken, deduktives Denken, Multitasking behindert, verlangsamtes Denken,
- Motorik: Bewegungen verlangsamt,
- Sprache: Wortfindungsstörungen, Verlangsamung,
- räumlich-visuelle, visuomotorische und -konstruktive Fähigkeiten,
- Gedächtnis: „Arbeitsgedächtnis“ – Kurzzeitgedächtnis beeinträchtigt.

#### 11.7.2 Ausschluss potenziell gefährlicher Verlauf („Red Flags“)

- Plötzliche deutliche Verschlechterung oder akutes Neu-Auftreten
- Auftreten multifokaler oder zentral neurologischer Symptome
- Hinweis auf reversible Ursache oder progrediente (Akut‑)Erkrankung?
- TIA bzw. Schlaganfall
- Akute Erkrankung (z. B. Enzephalitis)
- Hypoglykämie
- Akutes Delir
- Kognitive Verlangsamung im Rahmen einer schweren depressiven Episode? (Suizidale Gefährdung?)
- Denkhemmung im Rahmen einer schizophrenen Psychose
- Substanzgebrauch

#### 11.7.3 Zuordnung

##### Anamnese (nach Ausschluss von „Red Flags“ – mit Fremdanamnese wenn möglich)

- Inklusive Medikamenten- und Drogenanamnese sowie psychiatrischer Exploration

##### Klinische Untersuchung

- Gezielter neurologischer Status

##### Basisdiagnostik

- Ausschluss systemischer Ursachen (z. B. Anämie, respiratorische Insuffizienz, Schilddrüsenunterfunktion)
- Objektivierung der Gedächtnisleistungsstörung: einfach durchführbare kognitive Tests
  - Kurzer kognitiver Test zur Beurteilung einer möglichen Demenz [319]; z. B. 3 Worte, Uhrentest)
  - MMSE (Mini-Mental-Status-Examination) (über verschiedene Websites als pdf downloadbar)
  - MoCA (Montreal Cognitive Assessment) (über verschiedene Websites als pdf downloadbar)

#### 11.7.4 Procedere

##### Abwartendes Offenhalten

Dies ist nach Ausschluss potenziell gefährlicher Verläufe und reversibler Ursachen möglich, eine schrittweise Besserung (parallel mit der zunehmenden Normalisierung des zerebralen Glukosemetabolismus) ist in den meisten Fällen zu erwarten.

##### Fachspezifische Abklärung

- Bei Verdacht auf ein akutes zerebrales Geschehen oder akute reversible Ursache (vorhandene „Red Flags“) sollte eine entsprechende Abklärung entlang vorhandener Leitlinien inklusive zerebraler Bildgebung erfolgen.
- Die spezialisierte Abklärung wird bei gesicherter Neu-Manifestation/Therapieresistenz einer neurokognitiven Störung im Rahmen eines Long-COVID-Syndroms empfohlen. Dann erfolgt eine weiterführende Differenzialdiagnose nach den gültigen Leitlinien.
- Bei Persistenz der kognitiven Einschränkungen > 12 Wochen sollte eine Re-Evaluation erfolgen.

#### 11.7.5 Weitere wichtige Differenzialdiagnosen in Zusammenhang mit COVID-19

- Depression
- Biorhythmusstörung (Insomnie, fehlende Tagesstruktur)
- Andere organspezifische Ursachen als Folge von COVID-19
- Folgen eines Delirs im Rahmen der schweren Erkrankung
- Irreversible Schädigungen z. B. im Rahmen einer Hypoxämie/ARDS

#### 11.7.6 Weitere wichtige Differenzialdiagnosen ohne postinfektiöse Ätiologie

- Somatische Ursachen, z. B. Anämie, respiratorische Insuffizienz, Schilddrüsenfehlfunktion und metabolische Störungen, Blutdruckschwankungen
- (Meningo‑)Enzephalitis anderer Ursache (u. a. ZNS-Infektionen)
- Unerwünschte Neben- und Wechselwirkungen von Medikamenten oder anderen Substanzen
- Klinische Manifestation einer subklinischen Gehirnerkrankung (mit und ohne Assoziation zu COVID-19)
- Somatisierung
- Depression und Angststörungen

**Behandlung** siehe 12.3.7.

### 11.8 Schwindel („Vertigo“, „Dizziness“)

#### 11.8.1 Allgemeines

Primär ist zwischen Vertigo (Drehschwindel), Schwankschwindel und – im Sinne einer leichten Benommenheit – (Dizziness) zu unterscheiden. Im Zusammenhang mit COVID-19 werden eher der ungerichtete Schwindel und die „Dizziness“ beschrieben als die eigentliche Vertigo. Die englische Terminologie wird angeführt, weil die deutsche eine solche Unterscheidung nicht in ausreichendem Maße macht. Beschrieben wird hier der hausärztliche Abklärungsgang bei nicht eingeordnetem Präsentiersymptom Schwindel.

#### 11.8.2 Ausschluss potenziell gefährlicher Verlauf

- Relevante akute Beeinträchtigung – Vigilanz?
- Kurzanamnese bezüglich Schwindel – Charakter (Drehschwindel), Symptomdynamik (akut oder länger bestehend, plötzlich oder allmählich)
- Basiserhebung Vitalparameter (RR, HF rhythmisch?!, S_p_O_2_+AF, BZ)
- Auftreten gemeinsam mit anderen, multifokalen oder zentral neurologischen Symptomen?
  - Herzrhythmusstörung
  - TIA bzw. Schlaganfall
  - Neuritis vestibularis
  - Benigner paroxysmaler Lagerungsschwindel
  - Hypoglykämie
  - Dehydratation

#### 11.8.3 Zuordnung

##### Anamnese

- Lageabhängigkeit, Bewegungsabhängigkeit, Provokationsmöglichkeit
- Differenzierung Gleichgewichtsstörung/Schwindel/Kreislauflabilität (siehe dazu 11.8)
- Sturz- bzw. (gerichtete) Fallneigung?
- Begleitende vegetative Symptomatik (Übelkeit, Erbrechen)
- Dauer: rezidivierend? persistierend? → Bei Drehschwindel mit begleitender Hörminderung oder zusätzlichen neurologischen Symptomen ist eine spezialisierte Abklärung notwendig.
- Erfassung der subjektiven und objektiven Beeinträchtigung

##### Klinische Untersuchung

- Auskultation, gezielter neurologischer Status
- Auflösung/Provokation durch Lagerungsmanöver möglich? (BPLS)

##### Basisdiagnostik

- EKG bei Verdacht auf Herzrhythmusstörung, Blutdruckmessung inklusive liegend/stehend → „verkürzter Schellong-Test“ – 3 min aktiv stehend

#### 11.8.4 Procedere

##### Abwartendes Offenhalten

- Wenn keine zusätzlichen Symptome und keine Red Flags vorliegen, die beschriebene Basisuntersuchung ohne Ergebnis bleibt und die Beschwerden den Alltag nicht wesentlich beeinflussen, kann beim ungerichteten Schwindel auf eine weiterführende Diagnostik verzichtet werden. Dies gilt insbesondere, wenn die Störung erst im Gefolge der COVID-19 aufgetreten ist.

##### Fachspezifische Diagnostik

- Bei objektivierbaren kognitiven Störungen, Störungen der Vigilanz oder des Gedächtnisses (Außenanamnese!) wird eine neurologische Begutachtung empfohlen, vor allem wenn diese nicht klar in Zusammenhang mit der abgelaufenen Infektion stehen.
- Unspezifische „Dizziness“ bei anhaltenden, störenden Beschwerden und leitliniengerechtem Ausschluss alternativer Ursache mit hausärztlichen Mitteln: internistische/neurologische Abklärung erwägen.
- Zum Abklärungsgang bei gerichtetem Schwindel (Vertigo) wird auf die entsprechenden Leitlinien verwiesen (s. DEGAM-Leitlinie „Schwindel, akut in der Hausarztpraxis“ [320]).

#### 11.8.5 Weitere wichtige Differenzialdiagnosen in Zusammenhang mit COVID-19

- Orthostatische Dysregulation, POTS (siehe 8.10 AD, 8.2 Kardiologie, 11.9 Kreislauflabilität)
- Somatisierendes Verhalten: Angst‑/Panikattacken, Depression mit Schwindel als Manifestation einer Somatisierung
- Unspezifische Gangunsicherheit

#### 11.8.6 Weitere wichtige Differenzialdiagnosen ohne postinfektiöse Ätiologie

- Dehydratation, Anämie
- Verspannungen der Nackenmuskulatur
- Benigner paroxysmaler Lagerungsschwindel (BPLS)
- Orthostasereaktion (s. Abschn. 8.10 und 12.3.3.2)
- Kardiale Ursachen (Arrhythmie, myokardiale Genese etc.)
- Neuritis vestibularis, Morbus Menière
- Altersassoziierte unspezifische Schwindelsymptomatik
- Polypharmazie
- Angst/Panik
- Andere neurologische Ursachen (z. B. MS, Epilepsie, Polyneuropathie)
- Andere aktive/akute Infektionen oder Erkrankungen (Schilddrüsendysfunktion, Hypo- oder Hypertonie)

**Behandlung** siehe 12.3.3.2.

### 11.9 Kreislauflabilität

#### 11.9.1 Allgemeines

Kreislaufregulationsstörungen sind vor allem in den ersten Wochen nach der Infektion mit SARS-CoV‑2 nicht selten beschrieben [138]. Sie müssen von organischen, vor allem kardialen Krankheiten (auch: mögliche kardiologische Folgen von COVID-19) abgegrenzt werden sowie von der im Gefolge von COVID-19 häufig beschriebenen **autonomen Dysregulation **(Abschn. 8.10).

#### 11.9.2 Ausschluss potenziell gefährlicher Verlauf

- Plötzliche Einschränkung des Bewusstseins/Bewusstlosigkeit?
- Bei Synkope subjektive und objektive Beeinträchtigung (Vitalparameter: RR, HF, AF, S_p_O_2_, Blutzucker und Körpertemperatur)
- Weitere „Red Flags“: Arrhythmie, Stauungszeichen, Hautkolorit blass oder zyanotisch, neurologische Symptomatik

#### 11.9.3 Zuordnung

##### Anamnese

- Differenzierung zwischen Schwindel, Gleichgewichtsstörung, autonomer Dysregulation: Posturales Tachykardiesyndrom (POTS – Orthostaseintoleranz, Tachykardie bei Orthostase, Palpitationen, Schwindelgefühl, Sehstörungen, Präsynkopen und Belastungsintoleranz), orthostatische Hypotonie (OH) (siehe 8.10)
- Prodrome? Unter körperlicher Anstrengung? Potenzielle vasovagale Auslöser bzw. situativ erklärbar?
- Dauermedikation? Neue Medikation? Trinkmenge? Substanzgebrauch?
- Hinweis auf PEM (siehe dazu 8.9 und 11.1)
- Weitere Symptome (Müdigkeit/Fatigue, kognitives Defizit, mit Mastzellaktivierung vereinbare Symptome?)

##### Klinische Untersuchung

- Auskultation Herz/Lunge
- Blutdruckmessung, Schellong-Test (Abb. 2)
- Zeichen für Dehydratation
- Hinweise auf kardiorespiratorische Ursachen (Hautkolorit, Stauungszeichen, Anämie?)

##### Basisdiagnostik

- Basislabor inklusive BSG/CRP, EKG

#### 11.9.4 Procedere

##### Abwartendes Offenhalten

- Wenn Hinweise auf organische Ursachen in der Basisabklärung nicht gefunden werden, wenn die Beschwerden im Zusammenhang mit COVID-19 aufgetreten sind, den Alltag nicht stören (Klok-Skala < 2 s, Abb. 8) und keine weiteren Symptome bestehen, kann unter allgemeinen Maßnahmen (Bewegung, ausreichende Flüssigkeitszufuhr etc.) die Besserung abgewartet werden (s. dazu Behandlung der AD, siehe 8.10 und 12.3.3). Die Patient:innen sollten informiert werden, sich bei Verschlechterung oder zusätzlich auftretenden Symptomen wieder vorzustellen.

##### Fachspezifische Abklärung

- Sind die Kreislaufregulationsstörungen alltags- bzw. arbeitsrelevant (Klok-Skala > 2, Abb. 8), kann eine zeitnahe Abklärung erwogen werden, bei Persistenz über 12 Wochen hinaus ist die Re-Evaluierung aber jedenfalls empfohlen.
- Bei Hinweisen auf eine strukturelle bzw. Organsystem bezogene Erkrankung (neurologisch, pulmologisch, endokrinologisch, kardiologisch …) ist eine fachspezifische Abklärung entlang der üblichen Leitlinien empfohlen [321].
- Bei Hinweisen auf AD als postvirales Zustandsbild nach COVID und ausbleibender Besserung auf therapeutische Maßnahmen (siehe 8.10 und 12.3.3): durch eine spezialisierte Stelle oder eine spezialisierte Neurolog:in.

#### 11.9.5 Wichtige Differenzialdiagnosen In Zusammenhang mit COVID-19, z. B.

- Kardiale Genese nach COVID-19 (aber auch unabhängig davon möglich):
  - Herzrhythmusstörungen
  - Myokarditis
  - Herzinsuffizienz
- Postinfektiöse Thyreoiditis
- Postinfektiöse Fatigue

#### 11.9.6 Wichtige Differenzialdiagnosen ohne postinfektiöse Ätiologie, z. B.

- Dehydratation bzw. Elektrolytstörungen (z. B. Hyponatriämie), Anämie
- Andere aktive Infektionen
- Unerwünschte Neben- oder Wechselwirkung bei Polypharmazie
- Somatisierendes Verhalten im Rahmen einer psychischen Erkrankung (z. B. Angst/Depression)
- Schilddrüsendysfunktion
- Multifaktoriell, unspezifisch

**Behandlung** siehe 12.2 und 12.3.3.2.

### 11.10 Schlafstörung

#### 11.10.1 Allgemeines

Post COVID-19 werden Schlafprobleme häufig beschrieben. Die Ursachen sind vielfältig und bisher nicht sicher spezifisch mit der Viruserkrankung assoziiert [18].

#### 11.10.2 Ausschluss potenziell gefährlicher Verlauf

##### Red Flags

Ausgeprägt depressive, manische oder psychotische Komponente, schädlicher Medikamenten- oder Drogenkonsum.

#### 11.10.3 Zuordnung

##### Anamnese

- Subjektiv zeitlicher Zusammenhang mit COVID-19 oder Verstärkung vorbestehender Beschwerden
- Ein- oder Durchschlafstörung, Störung des Schlafrhythmus, Tagesmüdigkeit, vorzeitiges Erwachen
- Atemaussetzer – auch ohne Schnarchen (Fremdanamnese), Schlaf tagsüber
- Fragen nach negativen Gedanken, Gedankenkreisen, Angst, Aufgeregtheit. Gefragt wird auch nach Schlafgewohnheiten, Lebensstil inklusive Medikamenten, psychosozialen belastenden Faktoren mit oder ohne Zusammenhang mit der Pandemie und störenden körperlichen Sensationen (Juckreiz, Schmerzen, „restless legs“, Atemnot etc.). Sorgen bezüglich eines eventuell behindernden Verlaufs von COVID-19, s. dazu Leitlinie Schlafstörung der DEGAM, Anwenderversion [322]

##### Klinische Untersuchung

- HNO-Bereich, BMI, RR

##### Weitere Untersuchungen

- Nur bei Hinweisen auf somatische Ursache: Schlaftagebuch, Schlaflabor

#### 11.10.4 Weitere wichtige Differenzialdiagnosen ohne postinfektiöse Ätiologie

- Vorbestehende schlafmedizinische Probleme (Einschlafstörung, Durchschlafstörung, nicht erholsamer Schlaf, Tagesmüdigkeit) oder psychische Probleme können im Rahmen der Pandemie verstärkt werden oder stärker empfunden werden.
- Selten: somatische Ursachen (Hinweise aus der Anamnese). Siehe dazu S3-Leitline der Deutschen Gesellschaft für Schlafforschung und Schlafmedizin e. V. (DGSM) [323].

**Behandlung** siehe 12.2 und 12.3.

### 11.11 Nerven- und Muskelaffektionen

#### 11.11.1 Allgemeines

Myalgien kommen im Akutstadium der COVID-Infektion häufig (~20 %) vor. Circa 50 % der Patient:innen erholen sich von ihren Myalgien innerhalb von wenigen Tagen. In ca. 6 % der Fälle werden persistierende muskuloskeletale Beschwerden berichtet [143].

Die Nerven und Muskelaffektionen können sehr schmerzhaft sein und bei den Patient:innen eine große Belastung hervorrufen. Im Rahmen von Post-COVID-Zuständen wurden z. B. Hinweise für eine Myopathie in Muskelbiopsien von Betroffenen gefunden. Inwieweit Entzündungsreaktionen und kapilläre Läsionen zu Fatigue und Schmerzen beitragen können, wird diskutiert [279].

Aber auch Small-Fiber-Neuropathie wurde im Zusammenhang mit Long COVID beschrieben sowie Verschlechterungen und/oder Neuauftreten von Erkrankungen, welche mit Nerven- und Muskelaffektionen einhergehen.

#### 11.11.2 Ausschluss potenziell gefährlicher Verlauf

- Sensible Defizite, motorische Defizite, Bewusstseinseintrübungen, kognitive Defizite, Wesensveränderung, Agitation & Delir, Sehverschlechterung, Bewegungsstörungen, Sprachstörungen, Schluckstörungen, Koordinationsstörungen, (nicht‑)konvulsive Anfälle, Urin- und Stuhlinkontinenz, kardiovaskuläre Komplikationen, Arrhythmien, respiratorische Insuffizienz
- Blasse, kalte Extremität bei deutlich unterschiedlichen Fußpulsen, Schmerzen oder Schwellung, rote, dicke Extremität im Seitenvergleich

#### 11.11.3 Zuordnung

##### Anamnese

- Zeitpunkt des Auftretens, Lokalisation, Dauer, Dynamik, Belastungsabhängigkeit, Provokations‑/Linderungsfaktoren, Ansprechbarkeit auf Schmerztherapie
- Bewegungsanamnese
- Weitere Symptome (besonders Fatigue 11.1, chronische Schmerzen, autonome Dysfunktion 8.10, Kreislauflabilität 11.9)
- Vorerkrankungen, Spitalaufenthalt, Familienanamnese, Medikamente (z. B. Statine), Alkohol, andere toxische Einwirkungen

##### Klinische Untersuchung

- Zur Objektivierung der subjektiv empfundenen Beschwerden:
- Je nach Anamnese: Suche nach Zeichen zugrunde liegender Erkrankungen (Differenzialdiagnosen s. weiter unten)
- Sorgfältige klinische Untersuchung inklusive Motorik, Sensibilität, Reflexe und Durchblutung im Seitenvergleich

##### Weitere Untersuchungen

- Labor: BB, BZ, evtl. CRP-, D‑Dimer-Schnelltest bei konkreten klinischen Hinweisen, evtl. CK (Rhabdomyolyse)
- Weitere Laborwerte sowie Anwendung von bildgebenden und apparativen Verfahren je nach Verdachtsdiagnose

#### 11.11.4 Procedere

##### Abwartendes Offenhalten

- Wenn eine erste Abklärung keine Hinweise auf neurologische/internistische/orthopädische Pathologien erbringt, kann in Absprache mit den Patient:innen mit einer weiterführenden Abklärung zugewartet werden (wichtig ist das Fehlen von „Red Flags“ und auch sonst unauffälligen Untersuchungsergebnissen).
- Eine klinische Kontrolle bei der Hausärztin/beim Hausarzt wird je nach Zustandsbild empfohlen. Eine Persistenz über 12 Wochen sollte zur Re-Evaluierung führen.
- Bei deutlicher Beeinträchtigung im Alltag: evtl. frühere Weiterleitung an spezialisierte Ambulanz oder spezialisierte Neurolog:in.
- Auf die Notwendigkeit der Wiedervorstellung bei Verschlechterung ist dokumentiert hinzuweisen!

##### Fachspezifische Abklärung

- Critical-Illness-Neuro-Myopathie (ICU – Aufenthalt? Dauer ICU-Aufenthalt? Muskelatrophien? Areflexie?)
- Rhabdomyolyse (wenn CK > 10.000 U/l, Nierenverschlechterung, Harn braun)
- Guillain-Barré-Syndrom (Von distal symmetrisch aufsteigende Par- und Hypästhesien bis hin zu schweren Tetraparesen. Auch bilaterale Fazialisparesen, Augenmuskelparesen oder Miller-Fisher-Syndrom. Respiratorische Insuffizienz)
- Konus‑, Cauda-Syndrom, Reithosenanästhesie
- TVT, PAVK

Zur weiteren spezialisierten Abklärung einer unklaren Myalgie siehe „Diagnostik und Differenzialdiagnose bei Myalgien“, S1-Leitlinie der Deutschen Gesellschaft für Neurologie (DGN): [171].

#### 11.11.5 Differenzialdiagnosen (mit und ohne Zusammenhang mit einer SARS-CoV-2-Infektion)

- Nerven- und Muskelaffektionen im Rahmen der Verschlechterung einer vorbestehenden Erkrankung
- Small-Fiber-Neuropathie (offener Stellenwert)
- Persistenz der Schwäche und Muskelatrophie nach Spitalaufenthalt oder ICU
- (Verschlechterung eines vorbestehenden) muskuloskeletalen Schmerzsyndroms, Wirbelsäulen‑, Gelenkbeschwerden
- Arthritis, Arthrose, rheumatologische Erkrankungen
- Polyneuropathie, z. B. im Rahmen von Diabetes mellitus Typ II, Hypothyreose, Alkoholabusus, Vitamin‑B_12_-Mangel (selten B_6_, B_1_, E), hereditär, bei HIV-Infektion
- Myositis, Dermatomyositis, systemische Autoimmunerkrankung
- Medikamentennebenwirkungen (insbesondere Statine, Ciprofloxacin, Bisphosphonate, Aromatasehemmer, Fibrate)
- Myopathien, Schilddrüsenerkrankung, Nebenniereninsuffizienz, Vitaminmangel, Leber- oder Nierenerkrankungen, Elektrolytverschiebungen, Krebserkrankungen
- Verschlechterung der körperlichen Belastbarkeit wegen z. B. Bewegungsmangel/vermehrten Sitzens während der Pandemiemaßnahmen und psychischer Faktoren
- TVT, PAVK

**Behandlung** siehe 12.1, 12.2 und 12.3.

## 12. Behandlung

### 12.1 Übersicht

Eine kausale Behandlung bei anhaltenden Symptomen im Sinne einer postviralen Erkrankung nach COVID-19 ist noch nicht bekannt. Das Management erfolgt symptomorientiert sowie in der Anleitung zum Selbstmanagement, in Beratung und Begleitung. Für einige der Symptome werden neue medikamentöse Ansätze vorgeschlagen, belastbare kontrollierte Studien sind bisher nicht publiziert, jedoch wird derzeit eine Reihe von Wirkstoffen untersucht:

Fawzy et al. fanden in ihrem Systematic Review [324] zu laufenden Studien zu therapeutischen Möglichkeiten hinsichtlich Post-Acute-COVID-19 388 registrierte Studien. Diese Studien weisen ein hohes Maß an Heterogenität auf. 144 einzelne Maßnahmen für PACS werden untersucht. Die meisten zielen auf eine allgemeine Linderung der Symptome ab und sind daher für einzelne PatientInnen unspezifisch. 331 Studien untersuchten monotherapeutische Strategien, während 39 Studien eine Kombination von Interventionen beinhalteten. Es besteht weiterhin ein Bedarf an qualitativ hochwertigen und methodisch soliden Studien mit stringenteren Einschlusskriterien für Subpopulationen und standardisierten Outcome-Parametern unter Berücksichtigung der aktuellen WHO-Empfehlungen [325], um in Zukunft spezifische Behandlungsempfehlungen geben zu können. Weitere Hintergründe zur Pathophysiologie und zu möglichen Therapien werden bei Davis et al. diskutiert [77].

#### 12.1.1 Zentrale therapeutische Ansätze im Überblick

- Details siehe 12.2 und 12.3
- Wenn eine dem Symptom zugrunde liegende Pathologie identifiziert werden kann, erfolgt die Behandlung entsprechend diesem Befund nach den üblichen Regeln und Leitlinien.
- Des Weiteren stehen die symptomatische Therapie mit begleitender Patient:innen-adaptierter Unterstützung sowie eine adäquate Aufklärung über die Prognose im Vordergrund (siehe dazu 12.2 und 12.3).
  - Für Fatigue und für autonome Syndrome sollte eine medikamentöse Therapie jedenfalls nicht Therapie erster Wahl sein (siehe 12.2.1 und 12.3.3).
  - Für Symptome, die mit dem Konzept der Mastzellüberaktivierung vereinbar sind, kann eine probatorische Therapie mit H_1_- oder H_2_-Rezeptorenblockern angeboten werden (siehe 12.3.4).
- Als wichtige therapeutische Maßnahme bei Vorliegen von Post-Exertional-Malaise (PEM) oder „post-exertional symptom exacerbation“ (PESE), z. B. im Rahmen einer chronischen Erschöpfung/Fatigue, autonomer Dysfunktion, kognitiven Störungen und anderen Symptomen von PASC wird die Aufklärung und Instruktion von „Pacing“ empfohlen (siehe dazu 12.2.1).
- Wenn PEM/PESE ausgeschlossen werden kann (siehe 11.1), wird eine Aktivierungstherapie („graded exercise“) angestrebt (siehe 12.2.2).
- Ein weiterer therapeutischer Ansatz ist die Unterstützung der **Wiedereingliederung in den Alltag bzw. die Arbeitswelt bzw. die Rehabilitation** (Kapitel 13).

#### 12.1.2 Behandlung im primärversorgenden Bereich – Überblick und Eckpunkte

- Jeder Behandlungsentscheidung geht die sorgfältige Abklärung der Ätiologie voraus (Kapitel 8 und 11 Differenzialdiagnostik).
- **Behandlung struktureller Erkrankungen** (Aggravierung vorbestehender Komorbiditäten oder neu aufgetretene organische Störungen) je nach Situation im hausärztlichen oder spezialisierten Setting oder in interdisziplinärer Kooperation.
- **Behandlung von Störungen**, die als Folge der Infektion mit SARS-CoV‑2 identifiziert wurden und keine alternative Diagnose aufweisen („post-COVID condition“): Betreuung und Monitoring vorzugsweise im Team der hausärztlichen Primärversorgung [18, 19]. Über die in den meisten Fällen gute Prognose sollten die Patient:innen informiert werden. Eine Objektivierung des individuellen Leidensdrucks und des Ausmaßes der Beeinträchtigung bildet eine weitere Entscheidungsgrundlage für die Wahl der Behandlungsstelle (siehe Kapitel 9). Die Post-COVID-19-Skala des funktionellen Status („Klok-Skala“, Abb. 8) als validiertes Tool hilft bei der Bewertung der bestehenden Leistungseinschränkung.
  - Auch wenn keine wesentliche funktionelle Einschränkung vorliegt, soll eine **Coping**strategie gefunden werden, siehe 12.2.3, anlassbezogene oder auch terminlich fixierte Kontrollen sollten den Betroffenen empfohlen werden.
  - Die Ermittlung und Berücksichtigung **psychosozialer Umstände**, ob durch die Infektion oder die Pandemie und ihre Folgen bedingt oder auch davon unabhängig bestehend, sind essenziell und Teil guter hausärztlicher Praxis.
  - Zuziehung von **Gesundheitsberufen** (Ergotherapie, Physiotherapie, Psychotherapie …) situationsabhängig.
  - **Pacing bei PEM/PESE** siehe dazu 12.2.1.
  - **Aktivierende Therapie („graded exercise“) nach Ausschluss von PEM/PESE** (2.2.3) und insbesondere bei POTS, das ebenfalls mit einer Belastungsintoleranz einhergehen kann. Hier wäre eine entsprechende aktivierende Therapie, abhängig von der Tolerabilität, auch bei Belastungsintoleranz indiziert (siehe 12.3.3).
  - **Medikamentöse Behandlungsansätze** zur Therapie spezieller Symptome siehe 12.3. Medikamentöse Therapien sind (insbesondere im primärversorgenden Bereich) meist nachrangig. Vorrangig sind Patientenberatung, nichtpharmakologische Ansätze und Patientenführung, siehe dazu 12.2.1, 12.2.2 und 12.2.3.
  - **Kooperation mit spezialisierten Stellen** zur Behandlung typischer COVID-assoziierter Beschwerdebilder (siehe Kapitel 12): nach Prüfung von Indikation und Eignung der Patient:in mit Vorbefunden (siehe 9.2).
  - Die Einleitung **ambulanter oder stationärer Rehabilitation**smaßnahmen (siehe Kapitel 13) sollte ab einer Beeinträchtigung 2. Grades auf der Post-COVID-19-Skala des funktionellen Status (Klok-Skala, Abb. 8) überlegt werden, wenn die Beschwerden mehr als 3 Monate andauern und keine klare Besserungstendenz ersichtlich ist. Bei starker Beeinträchtigung auch schon früher.
    Je schwerer der Verlauf der Akuterkrankung, desto wahrscheinlich wird die Notwendigkeit einer strukturierten Rehabilitation. Auch hier ist es wiederum wichtig, das Vorliegen einer PEM oder PESE zu berücksichtigen. Die Rehabilitation muss jedenfalls individualisiert, personen- und kontextsensibel aufgebaut sein (siehe Kapitel 9).
  - Wenn sich eine langfristige Problematik zeigt, sollte die Überweisung zu einer Anlaufstelle erfolgen, die auf postvirale Zustandsbilder spezialisiert ist, psychotherapeutische und/oder ergotherapeutische Begleitung angeboten werden, siehe Kapitel 9. Auch das Hinzuziehen von Hauskrankenpflege, Heimhilfe oder Sozialarbeit kann hilfreich sein.
  - Es wird eine Reihe von Behandlungsansätzen angeboten wie Nahrungsergänzungsmittel, pflanzliche Wirkstoffe, für die Belege für eine Wirksamkeit großteils fehlen, sowie homöopathische Mittel, wo Belege für eine Wirksamkeit gänzlich fehlen. Auch bei diesen pflanzlichen Substanzen oder Nahrungsergänzungsmitteln können schädliche Neben- und Wechselwirkungen nicht grundsätzlich ausgeschlossen werden [19].
  - Zu Versorgungsorganisation, Kooperationen und Zuständigkeiten: Kapitel 9 Versorgungsweg.

#### 12.1.3 Spezielle medikamentöse Optionen (keine robuste Evidenz, Off-label-Therapie)

- Für einige der Hauptsymptome von Post COVID wird empirisch bzw. im Rahmen von Studien eine Reihe von Medikamenten eingesetzt. Diese sind noch wenig untersucht. Der medikamentöse Zugang ist in der Regel Second-Line-Therapie.
- Aufgrund der unzureichenden Studienlage kann diese Leitlinie derzeit keine Empfehlungen zur medikamentösen Therapie geben.
- Therapeutische Impfung: Die Datenlage zu einer therapeutischen Vakzinierung ist derzeit unzureichend. Diese ist daher Studien vorbehalten.

##### Empfehlung

Die Behandlung sollte entsprechend der Ursache erfolgen. Wenn eine solche nicht bestimmt und/oder behandelt werden kann, sollte die symptomatische Behandlung angeboten werden.

##### Empfehlung

Im Vordergrund bei der Behandlung postviraler Zustände nach COVID-19 stehen nicht-medikamentöse Optionen.

##### Empfehlung

Medikamentöse Therapieformen sind in Erprobung, Empfehlungen können derzeit noch nicht formuliert werden

##### Empfehlung

Behandlung, Begleitung und Monitoring sollten jedenfalls erfolgen, auch wenn die Symptomatik unklar erscheint und/oder ein kausaler Zusammenhang mit COVID-19 nicht gesichert werden kann. Das Behandlungskonzept wird individuell geplant: entsprechend den Ergebnissen der Abklärung und in Zusammenschau mit subjektivem Leidensdruck und den Vorstellungen und Möglichkeiten der Betroffenen.

##### Empfehlung

Wenn von Patient:innen Wünsche nach nicht überprüften therapeutischen Konzepten geäußert werden, sollten diese auf mögliche schädliche Wirkungen überprüft werden (soweit dies möglich ist) und ansonsten sollte offen und realistisch erklärt werden, dass es keine Belege für deren Wirksamkeit gibt, und darauf aufmerksam gemacht werden, wenn sich mögliche schädliche Wirkungen nicht ausschließen lassen.

### 12.2 Allgemeinmaßnahmen im Detail

#### 12.2.1 Pacing bei gesichertem Vorliegen von Post-Exertional-Malaise (PEM) und/oder Post-Exertional-Symptom-Exazerbation (PESE), ohne autonome Dysfunktion

Die Indikation für ein Pacing muss sorgfältig gestellt werden – das Vorliegen von PEM/PESE ist genau zu prüfen (mittels aufmerksamer Anamnese und Fragebogen-gestützt), da Pacingtherapie bei Fatigue ohne PEM bzw. bei AD kontraproduktiv sein und zu einer erheblichen Verzögerung der Wiederherstellung führen kann (s. dazu 8.9 und 8.10).

Andererseits führt die Nicht-Beachtung eines vorhandenen PEM zu einer für einige Betroffenen erheblichen und vor allem auch anhaltenden Verschlechterung ihres Zustands (siehe 8.9).

Pacing ist ein personenzentriertes Verfahren, das Patient:innen ermöglichen kann, ihre körperliche, kognitive und emotionale Energie innerhalb individueller Grenzen zu steuern durch sorgfältige Planung, wo und wie die verfügbare Energie eingesetzt werden kann. Es ist ein Instrument, um bei Vorliegen von Post-Exertional-Malaise und/oder Post-Exertional-Symptom-Exazerbation eine längerfristige oder dauerhafte Zustandsverschlechterung/Zunahme von Symptomen zu verhindern.

Ein engmaschiges ärztliches Follow-up ist essenziell und ermöglicht die individuelle Anpassung der Therapie sowie das Erreichen von Therapiezielen.

**Durchführung**:

Das Pacing erfolgt symptomgesteuert. Aktivitätsprotokolle sowie Herzfrequenz- und Aktivitätsmonitore können verwendet werden, jedoch nur, um den Patient:innen zu verdeutlichen, wann sie ihre spezifischen Energiegrenzen überschreiten. Vorsicht ist bei der Verwendung von solchen Hilfsmitteln angebracht, da Patient:innen sich vor allem an ihren Wahrnehmungen orientieren sollen, nicht an Messwerten, die bei disponierten oder nicht ausreichend sorgsam geführten Personen in eine Fixierung führen können. Pacing ist eine anspruchsvolle Aufgabe, und Rückschläge sind unvermeidlich, zumal die Toleranzgrenze für Aktivität interindividuell und auch intraindividuell von Tag zu Tag variieren kann. Zudem: Die Zustandsverschlechterung setzt oft erst zeitverzögert ein, v. a. bei kognitiven Symptomen, Zusammenhänge zur auslösenden Aktivität sind oft schwer einzuschätzen. Pacing kann ergotherapeutisch unterstützt werden, vor allem bei Problemen in Vermittlung oder Anwendung (graduieren von Tätigkeiten, Betätigungsbalance im Kontext der aktuellen Gesundheitssituation, Beratung zu Umweltmodifikationen und Prioritätensetzung im Alltag in Verbindung mit Energie- bzw. Fatigue-Management).

Die derzeit größte Herausforderung stellt das gleichzeitige Vorliegen einer Belastungsintoleranz und autonomer Dysfunktion dar. Engmaschige Kontrollen, sorgsame Begleitung ermöglichen die Abgrenzung eines PEM/PESE von einer AD-bedingten Belastungsintoleranz und damit die individuelle Anpassung der Therapie. Der Erforschung einer idealen Therapie in diesen Fällen sollte unbedingt Priorität eingeräumt werden, da die Patient:innen sonst schnell in eine Negativspirale entweder für das eine oder andere Krankheitsbild kommen.

##### Empfehlung

Personen, die infolge einer Infektion mit SARS-CoV‑2 an PEM oder PESE leiden, sollen in die Methode des Pacings eingeführt und entsprechend monitiert werden.*

* Das Vorliegen von PEM darf aus Sicht der Neurologie und Psychiatrie einer leitliniengerechten Therapie neurologischer und psychiatrischer Erkrankungen nicht im Wege stehen.

#### 12.2.2 Symptomtitriertes Training/Exercise Training („graded exercise“) (bei Fehlen von PEM/PESE sowie bei AD)

Wenn bei anhaltenden Symptomen eines postviralen Zustandes kein PEM vorliegt, sowie bei Vorliegen einer AD wird die Aktivierungstherapie angestrebt. Dabei sollen Belastungsgrenzen individualisiert und symptomorientiert ausgeweitet werden („graded exercise“). Parallel ist jedoch auf Energiemanagement im Alltag zu achten, um Überforderung zu vermeiden.

Dies gilt für alle Bereiche: körperliche und kognitive Leistungsfähigkeit, emotionale und mentale Belastbarkeit [326–328].

**Durchführung**:

- Langsame Wiederaufnahme von Alltagstätigkeiten und -belastungen auf niedrigstmöglichem Niveau, evtl. mit ergotherapeutischer Anleitung
- Steigerung des Niveaus, wenn die jeweilige Belastung über einen längeren Zeitraum gut toleriert wird (subjektiv und gemessen durch RR, HF, S_p_O_2_)
- Bei Verschlechterung der Symptome: Pause und Rückkehr zum absolvierbaren Niveau nach Abklingen der akuten Beschwerdesymptomatik („symptomtitriertes Training“)
- Evaluation einer Rehabilitationsmöglichkeit bzw. -notwendigkeit
- Individualisierte, personen- und kontextsensible Rehabilitation

##### Empfehlung

Personen, bei denen das Vorliegen von PEM/PESE mit hoher Wahrscheinlichkeit ausgeschlossen werden kann, sollten zu aktivierenden Therapieformen motiviert werden.*

* Das Vorliegen von PEM darf aus Sicht der Neurologie und Psychiatrie einer leitliniengerechten Therapie neurologischer und psychiatrischer Erkrankungen nicht im Wege stehen.

#### 12.2.3 Coping

Wesentlich ist die Vermeidung von unnötiger Angst und Unsicherheit aufseiten der Betroffenen. Dazu gehört die Information über die meist gute Prognose der Beschwerden ebenso wie die umfassende Information über die Sinnhaftigkeit eventueller Therapieangebote. Die Betroffenen müssen ernst genommen werden hinsichtlich ihres Leidensdruckes, berichteten Beschwerden muss im jeweils sinnvollen Ausmaß nachgegangen werden (s. Kap. 10 und 11). Berichte von Betroffenen weisen darauf hin, dass der Leidensdruck durch den Eindruck verstärkt wird, dass Symptome vonseiten der Behandler missinterpretiert oder in ihrer subjektiven Bedeutung für die Patient:innen unterschätzt werden. Eine vertrauensvolle Arzt-Patient-Beziehung ist immer, aber besonders in Situationen fehlender kurativer Therapieoptionen, für den Krankheitsverlauf von wesentlicher Bedeutung.

Ein individueller, biopsychosozial orientierter und kontextsensibler Behandlungsplan sollte immer dann gemacht werden, wenn Symptome als belastend empfunden werden und eine behandelbare Ätiologie nicht gefunden werden kann. Er kann folgende Bereiche umfassen (nach NICE [18]):

- Selbstmanagement der Symptome („was hilft mir“),
- Selbstkontrollen (Tagebuch, Pulsoxymeter etc.) unter Vermeidung einer Fixierung, s. oben,
- Vermittlung von Anlaufstellen,
- Unterstützungsmöglichkeiten („wer hilft mir“ – familiär, weitere Umgebung, professionell),
- Salutogenese („welche sind meine gesunden Anteile, was kann ich gut, wie und wo fühle ich mich wohl“),
- Empfehlung von verlässlichen und Warnung vor unzuverlässigen Internetquellen.

Je nach Situation können und sollen Angehörige anderer Berufsgruppen eingebunden werden.

##### Empfehlung

Angemessene Information darüber, dass in den meisten Fällen eine Besserung der Beschwerden von selbst eintreten wird, ist essenziell, ebenso aber ein Ernstnehmen des individuell empfundenen Leidensdrucks.

Die Vermeidung einer Fixierung auf die Symptome sowie von Übermedikalisierung (von Überdiagnostik bis Übertherapie) steht im Vordergrund.

### 12.3 Spezielle Behandlungsansätze im Detail

Im Folgenden werden die nach COVID-19 am häufigsten beschriebenen Symptome angeführt und Empfehlungen, meist aus der Erfahrungsmedizin stammend, zusammengefasst.

#### 12.3.1 Dyspnoe

(Differenzialdiagnostik Kapitel 11.4)

Der Einsatz von oralem Kortison muss im Einzelfall und nach pneumonologischer Indikationsstellung bei stagnierender Besserung und einer Bildgebung passend zu einer organisierenden Pneumonie erwogen werden [113].

Für den Einsatz einer antifibrotischen Therapie gibt es keine Evidenz.

Inhalierbare Kortikosteroide oder Betamimetika werden dann empfohlen, wenn es Hinweise auf eine obstruktive Komponente und/oder eine bronchiale Hyperreagibilität gibt (Anamnese, Klinik Spirometrie) und die Kriterien laut Leitlinien dafür erfüllt sind.

Eine milde bis moderate Dyspnoe ist nach COVID-19 nicht selten und remittiert normalerweise auch ohne Behandlung nach einigen Wochen. Ein Versuch mit dem beschriebenen Pacing (siehe 12.2.1) lohnt sich, wenn zusätzlich PEM oder PESE vorliegen. Wenn Dyspnoe im Rahmen einer POTS auftritt: Vorgehen unter 12.3.3 beschrieben.

**Figure 12:**
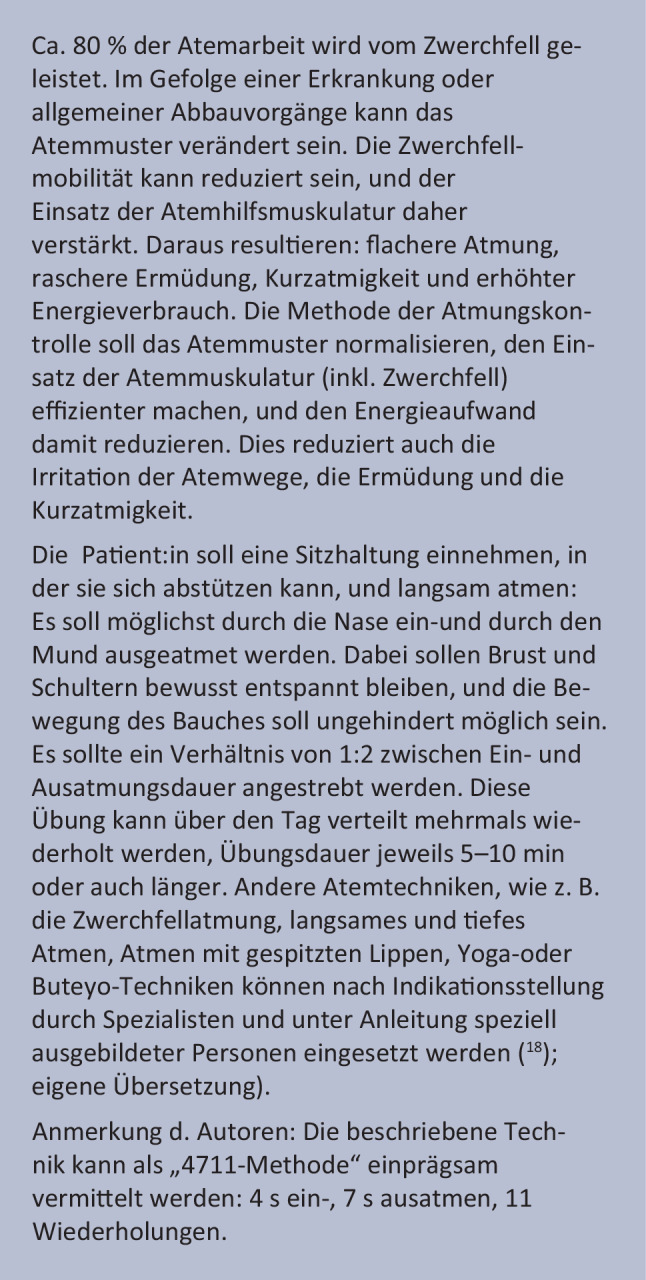

Das Erlernen von Atemtechnik (Abb. 12) kann Erleichterung schaffen.

Symptomatik und Leistungsfähigkeit von Patient:innen nach schwerem Verlauf bessern sich im Rahmen einer postakuten frühen, multimodalen Rehabilitation [329]. Des Weiteren ist eine multimodale Rehabilitation auch ohne Nachweis einer Organpathologie empfohlen, auch wenn die Evidenzlage für die Interventionen noch unzureichend ist [330].

Anleitung Atemtechnik: Abb. 12

#### 12.3.2 Leistungseinschränkung/Fatigue/mit oder ohne PEM

(Zur Differenzialdiagnostik Kapitel 11.1, Überblick Kapitel 8.9)

- Wenn keine organische Erkrankung als Ursache für die Leistungseinschränkung/Erschöpfung gefunden wird und sie als Einzelsymptom vorkommt, ist auch hier die Prognose in den meisten Fällen gut. Wie bei etlichen anderen Infektionskrankheiten bessert sich die Symptomatik im Laufe einiger Wochen, bei Persistenz > 12 Wochen ist eine Reevaluation notwendig.
- Wenn die Evaluierung keine zugrunde liegende Ätiologie erbringt (Details siehe 9.2) stehen Maßnahmen wie Coping (siehe dazu 12.2.3) und beim Vorliegen von PEM und/oder PESE Pacing (siehe 12.2.1) im Vordergrund. Bei autonomer Dysfunktion, und wenn kein PEM/PESE vorliegt, ist die korrekte Methode die symptomorientierte, begleitete Aktivierungstherapie (beschrieben unter 12.2.2). Die Evaluierung der psychischen und sozialen Situation sollte nicht vergessen werden.
- Bei Persistenz > 12 Wochen oder starker Beeinträchtigung: Begutachtung hinsichtlich weiterführender therapeutischer Ansätze, Überweisung an eine Stelle mit spezialisierter Expertise hinsichtlich postviraler Zustände (Kap. 9 Versorgungspfad). Wenn die Beeinträchtigung auf der Post-COVID-19-Skala des funktionellen Status (Abb. 8) mit 2 oder mehr eingestuft wird, sollten rehabilitative Maßnahmen erwogen werden. Siehe dazu auch die Fatigue Assessment Scale (Abb. 9). Bei der Entscheidung über Art und Zeitpunkt der Rehabilitation ist ebenfalls das Vorliegen einer PEM zu berücksichtigen.
- Nur nachgewiesene Mängel sind zu substituieren (z. B. Vitamin-D-Mangel), eine unbegründete Einnahme von Nahrungsergänzungsmitteln ist zu vermeiden, da Evidenzen fehlen und nur erste kleine Studien existieren, s. dazu 12.1.3.
- Bei gleichzeitigem Vorliegen einer PEM und/oder PESE: Pacing, s. Abschn. 12.2.1.
- Bei AD und bei Fehlen von PEM/PESE: symptomgetriggerte Aktivierungstherapie (beschrieben unter 12.2.2).
- Bei gleichzeitigem Vorliegen eines POTS als Ursache (Kapitel 8.10.4.1 und 12.3.3).
- Vorliegen einer mit dem Konzept der Mastzellüberaktivierung zu vereinbarenden Symptomatik: beschrieben in Kapitel 8.8.4, zur Behandlung unter 12.3.4; Behandlung begleitende Schlafstörung, Schmerzen, kognitive Dysfunktion – s. diesbezügliche Kapitel.
- Zu weiteren diskutierten Therapieformen s. Überblick in Kapitel 12.1.

#### 12.3.3 Autonome Dysfunktion

(Überblick Kapitel 8.10, Differenzialdiagnostik Kapitel 11.8 und 11.9)

##### 12.3.3.1 Posturales orthostatisches Tachykardiesyndrom (POTS)

(Überblick Abschn. 8.10.2.2)

Nach Diagnose eines POTS soll auf eine erhöhte Flüssigkeitszufuhr hingewiesen und über physikalische Gegenmanöver (in die Hocke gehen, Beine überkreuzen, ein Bein höherstellen, isometrischer Faustschluss) aufgeklärt werden. Eine salzreiche Ernährung kann empfohlen werden, insbesondere wenn keine Blutdruckspitzen in der 24-h-Blutdruckmessung bestehen. Des Weiteren können hüfthohe Stützstrümpfe oder eine Abdominalbandage verordnet werden. Oft bringen diese einfachen Maßnahmen schon signifikante Linderung. Aggravierende Faktoren wie größere, insbesondere kohlenhydratreiche Mahlzeiten, erhöhte Temperaturen (Sauna) und Alkohol sollen vermieden werden. Eine Dekonditionierung ist durch individuell angepasste Aktivität zu verhindern, die Evidenz für körperliche Betätigung ist stark.

Bei Fortbestehen der Symptome können unterschiedliche Pharmaka („off-label“) Anwendung finden je nach vorherrschendem Symptomkomplex: bei Tachykardie [295]: Propranolol („1st line“) oder Ivabradin, bei niedrigem Blutdruck im Liegen Midodrin („1st line“), bei Hypovolämie Fludrocortison („1st line“) und bei hyperadrenergen Zuständen Clonidin („2nd line“ – Achtung sehr niedrig dosieren).

Für die Behandlung von POTS-Patient:innen empfiehlt sich aufgrund der Komplexität des Krankheitsbildes und der vielen, aber allesamt nur „off-label“ verfügbaren Therapieoptionen die Anbindung an eine Spezialambulanz, die die Betreuung anbietet.

Zur Evaluierung des Managements hat sich, insbesondere nach Therapieänderung, die Durchführung einer 24-h-Blutdruckmessung bewährt. Des Weiteren ist empfohlen, dass Patient:innen ein Blutdruck/HF-Tagebuch führen, wo regelmäßige Liegend/Sitzend/Stehend-Messungen eingetragen sind, eventuell ergänzt durch ein Symptomtagebuch.

Kurzer Überblick über die Maßnahmen:

- Kompressionstherapie (hüfthohe Stützstrümpfe, abdominelle Bandagen)
- Salzreiche Kost (Steigerung auf 8–10 g/Tag) und Flüssigkeit (3 l/Tag) bei fehlenden Kontraindikationen, wie z. B. Blutdruckspitzen
- Physikalische Gegenmanöver (in die Hocke gehen, Beine überkreuzen, ein Bein höherstellen, isometrischer Faustschluss)
- Vermeidung von kohlehydratreichen Mahlzeiten, erhöhten Temperaturen (Sauna) und Alkohol
- Aktivierende Bewegung und Grundlagenausdauertraining nach individueller Verträglichkeit
- Medikamente haben noch keine Zulassung, zum eventuellen Off-label-Einsatz s. Abschn. 12.1.3. Je nach dominierender Symptomatik eventuell ein Therapieversuch nach entsprechender Aufklärung und Dokumentation. Sorgfältiges Abwägen von möglichem Nutzen und unerwünschten Wirkungen ist grundsätzlich Voraussetzung („off-label“!):
  - Tachykardie: (kardioselektive) β‑Blocker
  - Niedriger Blutdruck im Liegen: Midodrin
  - Hypovolämie: Fludrocortison (sollte nur von in autonomen Erkrankungen spezialisierten Behandlungszentren erfolgen)

##### 12.3.3.2 Orthostatische Hypotonie

(Grundlagen unter 8.10.4; Differenzialdiagnostik siehe 11.8 und 11.9)

- Prodromi wie Schwarzwerden vor Augen, Muskelschmerzen im Nacken etc. (individuell für Patient:in) BEACHTEN
- Körperliche Aktivität
- Schlafen mit erhöhtem Oberkörper
- Reichlich Flüssigkeitsaufnahme (Selbstkontrolle über Harnfarbe)
- Keine salzsparende Ernährung
- Physikalische Gegenmaßnahmen wie Partystand, Vornüberbeugen, ein Bein hochstellen, Hocke
- Hüfthohe Stützstrümpfe, Bauchbinde
- Zu vermeiden je nach individueller Verträglichkeit: kohlenhydratreiche Mahlzeiten, heiße Bäder/heiße Duschen/Sauna, Alkohol, rasches Aufstehen vor allem morgens
- Medikation: z. B. Midodrin wenn nicht-medikamentöse Maßnahmen nicht ausreichen und keine Kontraindikationen vorliegen insbesondere bei Patient:innen mit Liegendhypertonie

Für pharmakologische Interventionen bei OH gibt es, bis auf Midodrin und Droxidopa, nur geringe Evidenz [331]. Sollte eine pharmakologische Therapie indiziert sein, ist zuerst das Vorhandensein einer Liegendhypertonie festzustellen („supine hypertension“ [SH]). Dafür hat sich neben autonomer Funktionstestung insbesondere die 24-h-Blutdruckmessung bewährt.

SH bei OH wird eingeteilt in [332]:

- milde SH: systolischer RR 140–159 mm Hg oder diastolisch 90–99 mm Hg,
- moderate SH: systolischer RR 160–179 mm Hg oder diastolisch 100–109 mm Hg,
- schwere SH: systolischer RR ≥ 180 mm Hg oder diastolisch BP ≥ 110 mm Hg.

Wenn SH vorhanden ist, dürfen lang wirksame Substanzen wie Fludrocortison nicht verschrieben werden, eher sind Midodrin und Droxidopa zu wählen, um die OH zu therapieren [333]. Sollte SH behandlungsbedürftig sein, wird man initial nicht-pharmakologische Maßnahmen empfehlen (z. B. eine kohlenhydratreiche Jause vor dem Schlafengehen). Nur wenn nicht pharmakologische Maßnahmen nicht ausreichen, kann an pharmakologische Therapien wie Nitroglycerin bzw. Losartan gedacht werden [332].

##### Empfehlung

Bei Störungen im Bereich des autonomen Nervensystems werden als Erstlinientherapie physikalische und Lebensstilmaßnahmen empfohlen.*

* Die Co-Autorinnen Kathryn Hoffmann und Eva Untersmayr sind mit dieser Empfehlung in der vorliegenden Formulierung im Dissens.

#### 12.3.4 Mit dem Konzept der Mastzellüberaktivierung vereinbare Symptomatik („MCAS“)

Wenn belastende klinische Symptome vorliegen, kann entsprechend Expertenmeinungen ein Therapieversuch mit H_1_/H_2_-Blockern (Famotidin, Cetirizin, Desloratadin, Dimetindenmaleat oder Fexofenadin) unternommen werden. Obwohl auch hierfür keine belastbare Evidenz hinsichtlich der Wirksamkeit in dieser Indikation vorliegt, scheint eine probatorische Therapie gerechtfertigt, da es sich um gut erprobte und an Störwirkungen arme Wirkstoffe handelt. Bei ausbleibendem Behandlungserfolg wird die Medikation gestoppt. Wie immer und vor allem bei Off-label-Medikation erfolgen die exakte Beratung und Dokumentation der Entscheidung. Mehr dazu unter 8.8.4.

##### Empfehlung

Ein Therapieversuch mit H_1_- oder H_2_-Blockern erscheint bei belastenden Symptomen, die mit dem Konzept des MCAS vereinbar sind, gerechtfertigt.

#### 12.3.5 Husten

(Differenzialdiagnostik s. Kapitel 11.5)

Empirisch und analog zu den Empfehlungen bei postinfektiösem Husten kann ein Therapieversuch mit einem inhalativen Steroid eingeleitet werden. Beta-2-Sympathomimetika werden nur bei Indikation und entsprechend den Leitlinien eingesetzt. Bei fehlender Besserung weiterführende Abklärung und je nach deren Ergebnis gezielte Therapie nach pneumonologischer Begutachtung (siehe dazu 8.1). Bei anhaltendem Reizhusten ohne Substrat ist das Erlernen von Atemtechnik eine Option (Abb. 12). Auch eine logopädische Therapie wäre anzudenken.

#### 12.3.6 Sensorineurale Riechstörungen

(Differenzialdiagnostik Kapitel 11.2, Überblick Kapitel 7, 8.4)

Die Therapie COVID-19-bedingter Riechstörungen unterscheidet sich nicht von der Therapie sensorineuraler Riechstörungen anderer Genese. Da die Störung nach COVID-19 aber recht häufig ist, wird sie an dieser Stelle ausführlicher beschrieben.

Aufgrund der kontinuierlichen Erneuerung der Riechnervenzellen besteht eine hohe Regenerationsfähigkeit des Riechvermögens. Als eine in Studien wirksam bewertete Therapieform hat sich die *Durchführung eines strukturierten Riechtrainings* erwiesen [334]. Dazu sollen die betroffenen Patient:innen zumindest 2‑mal täglich über einen Zeitraum von jeweils 2 min an insgesamt 4 verschiedenen Duftölen riechen. Die entsprechenden Fläschchen können in der Apotheke oder Drogerie ohne Rezept gekauft werden (es eignen sich unterschiedliche Duftqualitäten, wie blumig, fruchtig, würzig, harzig, rauchig etc.). Aufgrund der komplexen zentralen Prozesse bei der Wahrnehmung von Duftstoffen, scheint die bewusste und konzentrierte Durchführung des Trainings wichtig zu sein. Es soll dadurch zu einer beschleunigten Re-Organisation der Verbindungen der Riechnerven im Riechkolben und in weiterer Folge in höheren zentralen Hirnarealen in Stirn- und Schläfenlappen kommen, die für die Wahrnehmung von Duftstoffen verantwortlich sind. Das Riechtraining sollte zumindest für 6 bis 9 Monate durchgeführt werden [335, 336].

Immer sind die Betroffenen darauf hinzuweisen, auf eine Nikotinkarenz zu achten, da Rauchen die olfaktorische Sensitivität vermindert [337].

Es existieren derzeit keine evidenzbasierten medikamentösen Therapien bei sensorineuralen Riechstörungen [194].

Auf jeden Fall sollten Patient:innen auf mögliche Gefahren der Riechstörung wie die verspätete Wahrnehmung von verdorbenen Lebensmitteln, Feuer, Verbranntem oder austretendem Gas hingewiesen werden [338]. Auch ist auf entsprechende Körperhygiene und bei Bedarf auch auf die Installation von Rauchmeldern zu achten.

Siehe auch Abb. 3.

##### Empfehlung

Bei postviralen Störungen des Riechvermögens soll die Durchführung eines strukturierten Riechtrainings angeboten werden.

#### 12.3.7 Kognitive Dysfunktion

(Differenzialdiagnostik Kapitel 11.7)

In diesem Bereich sind der Ausschluss von Differenzialdiagnosen und das aktive Nachfragen nach weiteren Symptomen besonders wichtig.

Symptome der kognitiven Dysfunktion kommen oft im Rahmen von POTS und/oder MCAS-like-Symptomen vor. Siehe dazu Kapitel 8.8 und 8.10.

Bei Vorliegen von PEM/PESE kann ein Therapieversuch mit kognitivem Pacing (ergotherapeutische Unterstützung) gemacht werden. Unbedingt Re-Evaluierung der diesbezüglichen Symptome nach 3 Monaten, da gerade in diesem hochsensiblen Bereich nichts verschleppt werden sollte.

Weitere diesbezügliche Therapieversuche [339] werden nur in Kooperation mit entsprechenden Fachärzt:innen bzw. Ärzt:innen mit Expertise in postviralen Erkrankungen empfohlen.

#### 12.3.8 Muskelschmerzen

(Differenzialdiagnostik Kapitel 11.11)

Die medikamentöse Therapie bei unspezifischen Muskelschmerzen ist zunächst rein symptomatisch, mittels (kurzzeitig) NSAR, Paracetamol und Metamizol. Diese soll die schrittweise Wiederaufnahme der täglichen körperlichen Aktivität unterstützen. Bei Therapieresistenz/Persistenz ist die Re-Evaluierung und evtl. Kooperation mit Kolleg:innen der Sonderfächer zu empfehlen, das Vorgehen erfolgt auch hier entsprechend den gültigen Leitlinien.

Patient:innen mit anhaltenden Beschwerden ohne PEM/PESE profitieren besonders von physikalischen Therapien/Physiotherapie.

Bei PEM Pacing-Ansatz beachten, um Post-Exertional-Malaise zu verhindern, welche zur Chronifizierung der Erkrankung führen kann, zum Pacing siehe 12.2.1.

#### 12.3.9 Hauterkrankungen

(Überblick Kapitel 8.5)

- Bei urtikariellen Exanthemen: niedrig dosierte systemische Kortikosteroide und Antihistaminika
- Bei konfluierenden, erythematösen/makulopapulösen/morbilliformen Exanthemen: topische und systemische Kortikosteroide
- Bei papulovesikulösen Exanthemen: „wait and see“
- Bei akralen Pernionen: „wait and see“
- Bei Livedo-reticularis/racemosa-Hautveränderungen: „wait and see“
- Bei vaskulitischen Hautveränderungen: topische und systemische Kortikosteroide
- Bei durch das Coronavirus getriggerten anderen dermatologischen Erkrankungen: Einleitung einer Therapie entsprechend den Leitlinien der einzelnen Dermatosen
- Bei Effluvium: symptomatische Therapie, wie z. B. topisches Minoxidil

## 13. Nachsorge und Rehabilitation

### 13.1 Allgemeines

Der übliche Weg nach COVID-19 ist die Wiederaufnahme der Berufs- bzw. Alltagstätigkeit nach Abklingen der akuten Beschwerden und Ende einer eventuellen Kontaktbeschränkung (legistische Grundlagen beachten!).

Bekannt ist, dass eine relevante Anzahl der Betroffenen auch nach als mild bezeichneten Verläufen ohne Hospitalisierungsnotwendigkeit noch über mehrere Wochen an Beschwerden leidet, in einer österreichischen Studie waren es 70 % – davon über 40 %, die mehr als ein Symptom angaben (Sample aus der Primärversorgung, inklusive 9 % hospitalisierte Patient:innen [33]), eine grundsätzliche Abklärung vor Wiederaufnahme der beruflichen Tätigkeit sollte zur Vermeidung einer Chronifizierung erwogen werden.

Die Differenzierung **organspezifischer Langzeitfolgen** (irreversibel oder Verschlechterung vorbestehender chronischer Erkrankungen) von funktionellen, meist reversiblen Folgezuständen ohne strukturelle Erkrankung ist für die Entscheidung zur Rückkehr zu körperlich beanspruchenden Aktivitäten (Beruf, Sport oder andere Aktivitäten) wesentlich, s. dazu Kapitel 8. Kardiale Folgen werden häufiger und in größerer Vielfalt beschrieben als zunächst angenommen (siehe dazu 8.2). Strukturelle Schädigungen müssen abgeklärt und behandelt werden, die Belastungsgrenzen müssen unter medizinischer Begleitung oder im Rahmen einer Rehabilitation ausgelotet werden.

**Nicht-organspezifische Störungen **(wie Fatigue, AD, MCAS, kognitive Beeinträchtigungen) benötigen meist Geduld und allgemein-rehabilitative Maßnahmen. Rückkehr- und Trainingspläne werden entsprechend Befunden und Klinik ausgerichtet (siehe 12.2 und 12.3), die Schwere des Verlaufs der Akuterkrankung spielt demgegenüber eine untergeordnete Rolle [18, 127, 340, 341].

Menschen mit PEM und/oder PESE brauchen zusätzlich immer Pacing, wobei in Kombination mit anderen Therapieformen immer „Pacing first“ gilt.

Wesentlich für weitere Entscheidungen sind die Beurteilung der Beeinträchtigung von Alltags- und Arbeitsfähigkeit aufgrund der Symptomatik und der subjektive Leidensdruck. Anmerkung: Die Symptomatik kann stark fluktuieren [18]. Die Post-COVID-19-Skala des funktionellen Status ist ein geeignetes Tool (Abb. 8; [342]).

### 13.2 Wiedereingliederung im hausärztlichen Setting

#### 13.2.1 Rückkehr in den Alltag (ohne wesentliche körperliche Belastung)

- Voraussetzung abgelaufene Differenzialdiagnostik, siehe Kapitel 11
- Alle Patient:innen nach COVID-19 sollten darüber aufgeklärt sein, dass persistierende Symptome auch nach mildem und moderatem Verlauf möglich sind, dass diese sich aber häufig im Verlauf von einigen Wochen, längstens Monaten zurückbilden.
- Einschränkungen der Leistungsfähigkeit sollten besprochen bzw. je nach Ausmaß abgeklärt werden (siehe 10.1 und 10.2). Bei starker oder anhaltender Einschränkung wird die Kooperation mit den jeweiligen Professionen (wie Ergotherapie, Psychologie, Psychotherapie, Sozialberatung) gesucht.
- Die Kernpunkte (nach Ausschluss relevanter struktureller Folgeschäden) sind:
  - Ist die Bewältigung der täglichen Aktivitäten möglich [340]?
  - Wie hoch ist die Alltagsbelastung (gemeinsame Abschätzung): Ausmaß der körperlich erforderlichen Fitness? Störungen der Kognition relevant für Arbeit/Selbstversorgung/Freizeitbeschäftigungen/Bedienen von Maschinen oder Transportmitteln?
  - Sind Möglichkeiten zur Erholung gegeben?
  - Gibt es Aktivitäten zur Gesunderhaltung und Stärkung der biopsychosozialen Gesundheit/Genesung im Alltag – sind sie durchführbar/organisierbar?
  - Fühlt sich die/der Betroffene psychosozial stark genug, um wieder arbeiten zu gehen?
  - Zur Stärkung von alltagsbezogenen Lebenskompetenzen sind die facheinschlägigen Professionen hinzuzuziehen. Dies betrifft insbesondere Ergotherapie, Physiotherapie, GuK, Sozialarbeit, Diätologie, evtl. Logopädie, Psychotherapie und Psychologie.

Als Technik zur Wiedererlangung von Alltagsfähigkeiten soll Pacing eingesetzt werden, das sich vor allem bei der Symptomatik bei Post-Exertional-Malaise (PEM) bewährt hat (Details Abschn. 12.2.1).

##### Empfehlung

Die Beratung hinsichtlich Selbstmanagement und die Planung des „Weges zurück“ in Alltag, Sport und Arbeit erfolgen individualisiert unter Berücksichtigung des persönlichen Kontextes. Empfohlen sind die Festlegung realistischer Ziele und klare Vereinbarungen über Belastungsgrenzen sowie ein Monitoring.

#### 13.2.2 Wiederaufnahme des Sports/Trainingstherapie

Auch für die Freigabe zum Sport muss das individuelle Risiko bewertet werden.

Organische Folgeschäden müssen ausgeschlossen werden, vor allem wenn eine kardiale Beteiligung vermutet wird.

Bei Symptomfreiheit nach der akuten Phase der Erkrankung gilt ansonsten:

##### Amateursport

Für die Rückkehr in den Sport wird der Schweregrad der Erkrankung als wichtig angesehen: Selbst bei asymptomatischer Erkrankung gilt eine wesentliche Belastungsreduktion bzw. Trainingspause für zumindest 3 Tage, bei milden Verläufen eine „absolute“ Trainingspause während anhaltender Symptome und danach für zumindest 3 symptomfreie Tage. Danach sollte weitere 2 Wochen auf intensive Belastungen verzichtet werden. Bei Auftreten von Beschwerden sollte zumindest der hausärztliche Kontakt gesucht und das weitere Vorgehen (Pacing) besprochen werden.

Mit zunehmender Schwere der Erkrankung steigt auch die Wahrscheinlichkeit für etwaige Komplikationen/Spätfolgen und somit auch die Dauer der empfohlenen Sportpause (z. B. bei Pneumonie: mindestens 4 Wochen, Myokarditis 3 bis 6 Monate). Im Konsensuspapier der sportmedizinischen Universitäts- und Landesinstitute Wien, Salzburg und Innsbruck wird daher relativ pragmatisch unterschieden, eine Sportpause bis zur völligen Genesung bei leichten, moderaten und schweren Verläufen empfohlen [343].

Nach vollständiger Genesung sollte die Rückkehr zum Sport **frühestens 7 bis 10 Tage** nach Erlangen der Symptomfreiheit angestrebt werden und über 2 Wochen mit noch deutlich reduzierter minimaler Belastung stattfinden [340, 344], die weitere Belastungssteigerung sollte auch in ausreichend großen Intervallen (bei milden Verläufen frühestens nach 3 Tagen [344], ansonsten wöchentlich) erfolgen. Bevor eine Rückkehr zur sportlichen Belastung angedacht werden kann, sollten die Aktivitäten des täglichen Lebens und die Absolvierung einer Wegstrecke von 500 m in der Ebene ohne Erschöpfungszeichen oder Atemnot absolviert werden können [53].

Als einfache Variante des Selbstmonitorings bei Belastung wird in der Literatur die Borg-RPE-Skala oder Borg-CR10-Skala (Abb. 10) für Laien angegeben. Weitere Hilfestellung können Ruhepuls und Pulsraten sein. Wichtig sind klare Vereinbarungen, wann sofort medizinischer Rat gesucht werden sollte. Kommt es zum Auftreten von Symptomen, sollte sofort wieder eine Belastungspause bis zu einer Symptomfreiheit von 24 h gemacht werden und auf die vorherige Belastungsstufe – welche ohne Symptome bewältigt wurde – zurückgegangen und ggf. neuerlich ärztlicher Rat gesucht werden. Nähere Details und hilfreiche Tipps dazu: BMJ – Return to play [53] und – ins Deutsche übersetzt – COVID-19 und return to play – Sportärztezeitung [345] sowie das Konsensuspapier der deutschen Gesellschaft für Sportmedizin und Prävention [346].

Bei Patient:innen mit Tachykardie, Orthostase, Belastungsintoleranz und/oder Dekonditionierung ist es empfehlenswert, zu Beginn Sport in liegender oder halbliegender Position auszuüben und eine aufrechte Position zu vermeiden [131]. Dies gelingt z. B. mittels Liegeergometer, Ruderergometer, Schwimmen, aber auch Radfahren am Ergometer ist möglich. Mit Besserung der Orthostasesymptomatik wird auch eine aufrechte Position immer mehr toleriert (s. dazu Abschn. 8.10).

##### Leistungssport

Für Athlet:innen wurde die Wiederaufnahme des Sports nach Infektionskrankheiten untersucht, hier gibt es Leitlinien aus der Europäischen Kardiologischen Gesellschaft [347] und der Deutschen Gesellschaft für Sportmedizin, die nach Infekt ein

- 12-Kanal-EKG,
- Echo,
- BB, Troponin, CRP

empfehlen.

Wenn diese negativ sind, dann gilt das Risiko eines kardiovaskulären Events in der Zukunft als sehr gering. In speziellen Fällen (Myokarditissymptome, Auffälligkeiten im Echo) sollte eine MRT des Herzens angeschlossen werden [347, 348]. Von pneumologischer Seite ist eine symptomorientierte Untersuchung (wie unter 8.1 beschrieben) ausreichend.

Bei Athlet:innen mit anhaltenden kardiopulmonalen Symptomen oder Hinweisen auf eine myokardiale oder perikardiale Beteiligung (im MR oder durch andere Untersuchungen) sollten vor Wiederaufnahme von Sport ein maximaler Belastungstest und ein ambulantes Rhythmusmonitoring erfolgen. Bei suspizierter Myokarditis darf der Belastungstest nach Ausschluss einer Myokarditis im MR durchgeführt werden.

Im Falle einer Myokarditis erfolgt eine 3‑ bis 6‑monatige Sportpause. Die Wiederaufnahme des Sports ist erst nach kardiologischer Freigabe möglich [131].

##### Empfehlung

Die Rückkehr zum Sport sollte auch bei völliger Genesung frühestens 10 Tage nach Erlangen der Symptomfreiheit angestrebt werden und über 2 Wochen mit minimaler Belastung stattfinden. Die weitere Belastungssteigerung sollte auch in ausreichend großen Intervallen (zumindest wöchentlich) erfolgen. Je schwerer die Akuterkrankung verlief, desto vorsichtiger ist der Weg zurück zu planen. Voraussetzung ist der Ausschluss organisch-struktureller Folgen der Erkrankung an COVID-19.

#### 13.2.3 Rückkehr an den Arbeitsplatz

- Für körperlich stark beanspruchende Tätigkeiten gilt sinngemäß die Empfehlung wie für die Wiederaufnahme des Sports.
- Nicht zu vergessen: Auch für die Modalität des Arbeitsweges (Gehen, Fahrrad fahren u. Ä.) gelten die gleichen Kriterien wie für den Beginn der sportlichen Belastung.
- Belastungsgrenzen und Berufseignung bei anhaltenden starken Einschränkungen sollten während einer Rehabilitation (Ergotherapie, Arbeitspsychologie) erhoben werden und je nach Situation vor Arbeitsantritt mit den zuständigen Präventivkräften im Betrieb (Sicherheitsfachkraft, Betriebsärzt:in) und den zuständigen Institutionen (Arbeitsinspektion, AUVA) besprochen werden. Je nach Situation können weitere Berufsgruppen für ein Wiedereingliederungsmanagement einbezogen werden (Case Management, Ergotherapie, Soziale Arbeit).
- In vielen Fällen können (vorübergehende) Anpassung von Arbeitsplatz und Arbeitsbedingungen den Wiedereintritt ins Berufsleben erleichtern bzw. vorverlegen. Auch hier sind die Präventivkräfte am Zug, idealerweise in Kooperation mit den hausärztlichen Primärversorger:innen.
- Eine Krankschreibung erfolgt nach den gleichen Grundsätzen wie immer, das Kriterium ist die tatsächliche, anforderungsbezogene Leistungsfähigkeit der Betroffenen. Die Diagnose sollte sich auf das jeweilige dominierende Symptom beziehen.

Eine Schwierigkeit, die sich aufgrund der derzeitigen Überlastung der Rehabilitationsstrukturen ergibt, ist die Überbrückung der Zeitspanne bis zum Antritt der Rehabilitation. Eine Arbeitsaufnahme in dieser Zeit wird für körperlich arbeitende Personen meist nicht gefahrlos möglich sein, woraus sich Probleme ergeben können. Die Begutachtung durch und gemeinsame Entscheidung mit Spezialisten je nach Gegebenheiten ist dringend empfohlen. Auch die Kontaktnahme mit Arbeitnehmerschutzeinrichtungen (AK, Gewerkschaft) sollte den Betroffenen angeraten werden. Case Manager der Krankenkassen können bei der Organisation der Wiedereingliederung unterstützen, soweit solche verfügbar sind.

Spekulative Annahmen über eine tatsächliche Arbeitsrückkehr sollten gegenüber den Arbeitgebenden aufgrund der unklaren Krankheitsdauer vermieden werden (daher maximal „voraussichtliche Rückkehr“), es sollte eher möglichst konstruktiv über eine gestufte Rückkehr in den Arbeitsprozess mit dem Arbeitgebenden gesprochen werden.

Bei Inanspruchnahme von Wiedereinstiegsmodellen wie die Wiedereingliederungsteilzeit (ÖGK) oder den Diensterleichterungen (BVAEB) empfiehlt es sich daher, von Beginn an den maximal möglichen Zeitraum anzunehmen: Verkürzt kann bei schnellerer Genesung immer werden, ein Verlängern des schrittweisen Wiedereinstiegszeitraums ist nur schwer möglich.

##### Empfehlung

Für die Planung der Rückkehr an den Arbeitsplatz sind neben Schwere der Akuterkrankung und weiter bestehender Symptomatik auch die individuellen Arbeitsplatzanforderungen und Arbeitsbedingungen zu berücksichtigen.

### 13.3 Rehabilitation

#### 13.3.1 Indikation

Anhand der Post-COVID-19-Skala des funktionellen Status (Abb. 8) lässt sich rasch die Indikation für rehabilitative Maßnahmen festmachen. Ab dem Stadium 2 können nach der ärztlichen Abklärung je nach Schweregrad Rehabilitationsverfahren beantragt werden, um eingeschränkte Körperfunktionen und Aktivitäten zu verbessern und eine bestmögliche Teilhabe in sozialer und beruflicher Hinsicht zu erreichen. Dies erfolgt anhand der Einteilung nach der International Classification of Functioning, Disability and Health (ICF) im Sinne einer biopsychosozial ausgerichteten medizinischen Trainingstherapie und multiprofessionellen Rehabilitation.

Besteht Rehabilitationsbedarf aufgrund pneumologischer, neurologischer, psychiatrischer oder kardiologischer postinfektiöser Schädigungen, kann eine indikationsspezifische Rehabilitation erfolgen. Die Therapien werden je nach Einschränkung von Körperfunktionen und Aktivitäten geplant und fokussieren auf die bestmögliche Teilhabe. Rehabilitationsverfahren erfolgen multimodal unter Einbeziehung verschiedener Fachdisziplinen.

Hierzu zählen z. B.: Physiotherapie, Trainingstherapie, Ergotherapie, Psychologie, Logopädie, Diätologie, Massage.

Die WHO teilt die Rehabilitation in 4 Phasen.

- **Phase I** entspricht der Mobilisation im Krankenhaus.
- **Phase II** kann als Anschlussheilverfahren entweder ambulant oder stationär erfolgen.
- **Phase III** ist eine Anschlussrehabilitation, die ambulant erfolgt, um die Nachhaltigkeit der Phase-II-Rehabilitation zu verbessern und die Situation bei schwereren Verläufen zu stabilisieren.

**Phase IV** bedeutet eine „Verstetigung“ in dem Sinne, als die Patient:in das Erlernte ein Leben lang weiterführen sollte.

Anhand der bisher publizierten Studien lässt sich folgende sehr allgemeine Empfehlung für die medizinische Trainingstherapie ableiten:

- Die Rehabilitation sollte Ausdauer- und Krafttraining beinhalten
- Krafttraining der großen Muskelgruppen mit 1 bis 2 Sätzen
- Ausdauertraining 5–30 min, 180 min/Woche bei etwa 5–8 MET (Metabolic Equivalent)
- Falls es zu Symptomen wie PEM kommt, ist die Dosierung im Sinne der Intensität und des Umfangs individuell und sofort anzupassen. Siehe Kapitel 12.2 zum Pacing. Aus diesem Grund sollten Patient:innen vorher auf das Vorliegen von PEM abgeklärt werden und entsprechend von Anfang an nur einer Rehabilitationseinrichtung zugewiesen werden, wenn diese Pacing anbietet. Falls nicht, muss mit der Zuweisung zur Reha gewartet werden, bis sich der Zustand des Patienten/der Patientin substanziell in Bezug auf PEM verbessert.
- Grundsätzlich sind spezifische Rehabilitationsangebote für Menschen mit PEM derzeit nicht verfügbar, dies sollte sich in Zukunft unbedingt im Sinne einer Stabilisierung und Verbesserung der Symptomatik auch für die Patient:innen ändern (s. auch angepasste Versorgungsleitlinie).

Eine detaillierte Beschreibung der Rehabilitationsmaßnahmen nach Infektion mit SARS-CoV-2/COVID-19-Erkrankung findet sich in der S2K AWMF Living Guideline und in der WHO Guideline [13, 325].

##### Empfehlung

Mit jeder Patient:in nach SARS-CoV-2-Infektion, die nach einem schweren oder kritischen Verlauf (= stationär/Intensivstation) nach Entlassung zur Erstbehandler:in im PCFS-Stadium ≥ 2 kommt, sollte ein Rehabilitationsantrag besprochen werden.*

* Die Co-Autorin Eva Untersmayr ist mit dieser Empfehlung in der vorliegenden Formulierung im Dissens.

##### Empfehlung

Jede Patient:in nach SARS-CoV-2-Infektion mit leichtem oder moderatem Verlauf (= ambulant), die im PCFS ≥ 2 zur Erstkontrolle kommt, sollte nach 4 bis 6 Wochen reevaluiert werden. Ist der PCFS immer noch/unverändert ≥ 2, ist eine Rehabilitation indiziert.*

* Die Co-Autorinnen Kathryn Hoffmann und Eva Untersmayr sind mit dieser Empfehlung in der vorliegenden Formulierung im Dissens.

#### 13.3.2 Evidenz

##### Pneumologisch

Long COVID mit **Dyspnoe, körperlicher Minderbelastbarkeit und/oder Fatigue** kann sowohl bei Patient:innen nach einem kritischen, aber auch nach einem milden Verlauf bestehen bleiben. Erste Publikationen konnten die Machbarkeit, Sicherheit und Effektivität von Rehabilitationsmaßnahmen nach einem schweren Verlauf zeigen [349–351]. Die Leistungsfähigkeit sowie lungenfunktionelle Einschränkungen konnten verbessert werden.

Nach einer stationären 3‑wöchigen pneumologischen Rehabilitation verbesserten sich sowohl körperliche Leistungsfähigkeit klinisch relevant (6-min-Gehtest: mittelschwer Betroffene +48 m [95 %-Konfidenzintervall, KI 35–113 m], schwer Betroffene +124 m [75–145 m] [349]) um im Mittel ca. 100 m, wie auch psychische Parameter wie Angst, Depression und Flashbacks. Dieselben Erfahrungen haben wir in 6 Wochen ambulanter Rehabilitation gemacht (Verbesserung 6‑min-Gehtest – NNT 1,4, Reduktion Fatigue – NNT 1,9, Verbesserung Dyspnoe – NNT 1,8, Verbesserung der PCFS – NNT 1,2). Weitere Studien zeigten eine Verbesserung der restriktiven Lungenfunktionsveränderungen, der Diffusionsstörung und der Atemmuskelkraft. Eine prospektiv randomisierte Studie mit inspiratorischem Atemmuskeltraining zeigte eine Verbesserung der Atemmuskelkraft und der Dyspnoe. Große prospektiv randomisierte Studien zu den Effekten einer multiprofessionellen Rehabilitation fehlen noch.

##### Neurologisch

Long-COVID-Patient:innen **mit Störungen von globalen oder spezifischen mentalen Funktionen, der Sprache, des Schluckens, der Motorik oder Sensorik** sollten einer neurologischen Evaluation und/oder neurorehabilitativen Versorgung zugeführt werden.

Bei kritischen Verläufen stellt das Post-Intensive-Care-Syndrom (PICS) eine bekannte und häufige Folge dar, die Einschränkungen auf die gesundheitsbezogene Lebensqualität und Teilhabe zu Folge hat [352]. Diese Patient:innen bedürfen nach klarer Definition von Rehabilitationszielen einer Früh‑/Rehabilitation.

**Kognitive Störungen** beim PICS [353] ebenso wie nach mildem oder moderatem Verlauf betreffen gehäuft Aufmerksamkeits- und Gedächtnis- sowie Exekutivfunktionen [354].

Zudem können in Zusammenhang mit COVID-19 verschiedene weitere spezifische Erkrankungen wie Schlaganfälle, Enzephalomyelitiden, ein Guillain-Barré-Syndrom (GBS), ein Miller Fisher-Syndrom, Hirnnervenneuritiden, Myositiden, eine Myasthenia gravis und Plexopathien auftreten, die alle mit spezifischem Rehabilitationsbedarf einhergehen.

##### Kardiologisch

COVID-19 kann mit schwerwiegenden kardiovaskulären Erkrankungen wie einer Myokarditis, einer Herzinsuffizienz, einem akuten Koronarsyndrom (ACS), Arrhythmien oder venösen Thromboembolien einhergehen [355, 356]. In diesen Fällen kann eine kardiologische Rehabilitation eingeleitet werden. Die Inhalte der kardiologischen Rehabilitation richten sich nach den Hauptindikationen wie Herzinsuffizienz, ACS, Myokarditis und thromboembolischen Erkrankungen (s. S3-Leitlinie zur kardiologischen Rehabilitation 2020 [357]).

Nach Ausloten der individuellen Belastungsgrenzen werden in Abhängigkeit von der zugrunde liegenden kardiologischen Problematik ein multimodales individuell adaptiertes Training bestehend aus Ausdauertraining (auf dem Fahrradergometer und/oder als Gehtraining) sowie ein Krafttraining jeweils mehrmals pro Woche angeboten. Bei schwer Betroffenen nach protrahierten Intensivaufenthalten kann auch ein Training der Inspirationsmuskulatur zur Anwendung kommen. Im Vordergrund steht die Erhöhung der Belastungstoleranz zur Förderung der bestmöglichen Teilhabe. Zusätzlich erfolgen eine diätologische sowie häufig auch eine psychologische Betreuung.

##### Psychiatrisch

Eine multimodale psychiatrische Behandlung ist angezeigt bei klinisch relevanten psychiatrischen Krankheiten im Rahmen von Long COVID, wie z. B. Depression, Angststörung, Zwangsstörung, Psychose oder PTSD. Diese umfasst unter anderem Psychopharmaka, Psychotherapie, Physiotherapie, Sozialarbeit und Arbeit mit dem sozialen Umfeld. Wenn ambulante Rehabilitationsmaßnahmen nicht ausreichen, ist eine stationäre Rehabilitation indiziert.

Empfehlung zum psychologisch/psychotherapeutischen Betreuungsausmaß in den unterschiedlichen Schwerpunkt-Rehabilitationseinrichtungen. *(Stufenmodell der psychologisch/psychotherapeutischen Betreuung in der Rehabilitation: S2k-Leitlinie SARS-CoV‑2, COVID-19 und (Früh‑)Rehabilitation – Living Guideline *[283]*)*

- Somatische Rehabilitationseinrichtung
  - Ausschließlich somatisch begründete Rehabilitation
  - Im Reha-Verlauf auftretende leichte depressive Verstimmung ohne Notwendigkeit einer intensiven psychologischen Betreuung/Psychotherapie
- Somatische Rehabilitationseinrichtung mit VOR-Schwerpunkt (verhaltensmedizinisch orientierte Rehabilitation)
  - Rehabilitation aufgrund vordergründiger somatischer Erkrankung mit begleitender Fähigkeitseinschränkung mit wesentlicher psychischer Komponente
- Psychosomatische Rehabilitationseinrichtung
  - Rehabilitation aufgrund psychischer Erkrankung (PTBS oder persistierende Angststörung nach SARS-CoV-2-Infektion) ohne rehabilitationsbedürftige somatische Folge der Infektion
- Duale Rehabilitation
  - Rehabilitation sowohl aufgrund psychischer als auch somatischer Folgeerkrankung

**Stufenmodell der psychologisch/psychotherapeutischen Betreuung in der Rehabilitation *(nach: S2k-Leitlinie SARS-CoV‑2, COVID-19 und (Früh‑)Rehabilitation – Living Guideline ***[283]***) ***(Tab. 3)

**Table 3:** 

Einrichtung	Patientenbeispiel
Somatische Rehabilitation	Leichte depressive Verstimmung und Verunsicherung bei somatisch begründetem Reha-Verlauf, Bedarf nach Patientenschulung, intensive psychologische Betreuung oder Psychotherapie ist nicht erforderlich
Somatische Rehabilitation mit VOR-Schwerpunkt (verhaltensmedizinisch orientierte Rehabilitation)	Somatische Erkrankung steht im Vordergrund, wird aber von einer wesentlichen psychischen Komponente der Fähigkeitseinschränkung begleitet (z. B. komorbide Depression oder Angststörung)
Psychosomatische Rehabilitation	Im Vordergrund steht die psychische Erkrankung, es besteht aktuell keine Reha-bedürftige somatische Infektionsfolge, z. B. persistierende Depression, Angststörung oder PTBS nach kompliziert verlaufender SARS-CoV-2-Infektion
Duale Rehabilitation	Patienten mit Reha-bedürftigen somatischen Folgeproblemen nach SARS-CoV-2-Infektion (v. a. auf pneumologischem, kardiologischem oder neurologischem Fachgebiet) und gleichzeitig bestehender Reha-bedürftiger psychischer Erkrankung (z. B. Depression, Angststörung)

## Abkürzungen

ACE-Rezeptor: Angiotensin-konvertierendes Enzym-Rezeptor
ACTH: Adrenocorticotropin
AD: Autonome Dysfunktion
ANS: Autonomes Nervensystem
BAL: Bronchoalveoläre Lavage
CDC: Centers for Disease Control and Prevention
COVID: Coronavirus infectious disease
EBV: Epstein-Barr-Virus
FAS: Fatigue Assessment Scale
HSV1/2: Herpes-simplex-Virus 1/2
MIS‑C: Multisystem inflammatory syndrome in children
mMRC: Modified Medical Research Council
NICE: National Institute for Health and Care Excellence
NYHA: New York Heart Association
OH: Orthostatische Hypotonie
PASC: Post-acute sequelae of COVID-19
PEM: Post-Exertional-Malaise
PESE: Post-exertional symptom exacerbation
PICS: Post intensive care syndrome
PIMS: Pädiatrisches inflammatorisches Multisystemsyndrom
POTS: Posturales Tachykardiesyndrom
RKI: Robert Koch-Institut
SARS-CoV‑2: Severe acute respiratory syndrome coronavirus type 2
WHO: World Health Organization

## Interessenkonflikt

S. Rabady, K. Hoffmann, M. Aigner, J. Altenberger, M. Brose, U. Costa, D.-M. Denk-Linnert, S. Gruber, F. Götzinger, R. Helbok, K. Hüfner, R. Koczulla, K. Kurz, B. Lamprecht, S. Leis, J. Löffler, C.A. Müller, H. Rittmannsberger, P.S. Rommer, P. Sator, V. Strenger, W. Struhal, E. Untersmayr, K. Vonbank, J. Wancata, T. Weber, M. Wendler und R.-H. Zwick geben an, dass kein Interessenkonflikt in Zusammenhang mit der Leitlinienerstellung besteht. Angaben zu Kooperationen und Verbindungen der Autor:innen: S. Rabady: Vortragshonorare: ÖGIM, Vielgesundheit.at, MUW, AK Pflegende Angehörige, Arztakademie; Gesellschafterin Fa. GELIAM – Contenterstellung f. „EbM-Guidelines“; unentgeltliche Beratungstätigkeit: Bundesministerium f. Gesundheit (OSR, Leitung AG postvirale Erkrankungen am OSR), GÖG, Förderungen Bundesministerium f. Gesundheit. M. Aigner: Gerot, Jansen-Cilag, Angelini. J. Altenberger: Vortragshonorare/Honorare bzw. Advisory Boards Novartis + Böhringer Ingelheim. F. Götzinger: Mitglied Advisory Board Fa. Biomerieux. R. Helbok: ACASTI, IDORSIA, Fresenius Kabi. K. Hüfner: KRKA. B. Lamprecht: Mitarbeit Advisory Boards AstraZeneca, Chiesi, Menarini; Vortragshonorare AstraZeneca, Boehringer Ingelheim, Chiesi, INSMED, Menarini, Pfizer, Roche, Sanofi. S. Leis: Advisory Board: Abbvie, Lilly, Lundbeck ; Klinische Studie: Biohaven; Vortragshonorare/Reisekosten Abbvie, Lilly, Merz, Neuraxpharm, Novartis. J. Löffler: MSD, AOP, Jensen & Jensen, GSK, Astra-Zeneca, Chiesi, Boehringer. P.S. Rommer: Beratung/Vorträge: Alexian, Allmiral, Amicus, Biogen, Merck, Sanofi, Novartis, Roche, Sandoz, Horizon; Forschungsförderung: Amicus, Roche, Merck. V. Strenger: Advisory Boards/Beratertätigkeit MSC, Pfizer, Vifor; Vortragshonorare/Advisory Boards/Unterstützung Besuch Fortbildungen: Abbvie, Gilead, GSK, MSD, Pfizer. W. Struhal: Manz-Rechtsverlag, Springer, Oxford University Press; Vortragshonorare/Reisekosten: Donau-Universität Krems, ÖGN; Honorarium: Elsevier; Consultancy/Reisekosten: Roche (nicht mehr aktiv); Forschungsförderung: Karl Landsteiner Privatuniversität; Kassier-Stellvertreter, Österreichische Gesellschaft für Neurologie (ÖGN – oegn.at), Past president, European Federation of Autonomic Societies (EFAS – www.efasweb.com), Vize-Präsident, Otto Loewi Gesellschaft (OLG – www.olg.or.at), Chair, e-Communications Sub-Committee, World Federation of Neurology (WFN), Member, Ethics and Quality Task Force, (European Academy of Neurology, EAN – ean.org), Sekretär Österreichische Alzheimer Gesellschaft, Co-Chair, Arbeitsgemeinschaft Autonomes Nervensystem der ÖGN. E. Untersmayr: Desentum Oy; GEKA mbH, Nutrica, AllergoPharma, MacroArray Diagnostics. K. Vonbank: CSL Behring; finanzielle Unterstützung: Astra, CSL Behring, BI. T. Weber: Vortragshonorare/Advisory Boards/Kongressunterstützungen: Boehringer Ingelheim, Merck, Astra Zeneca, Pfizer, BMS, Novartis. R.-H. Zwick: Vortragshonorare Novartis, Chiesi, Boehringer. K. Hoffmann, U. Costa, S. Gruber und R. Koczulla: Vortragshonorare: BI, GSK, AZ, Grifols CSL, Ärztekammern, Hausärzteverband, Chiesi; Mitglied: Fachgesellschaft DGP, DGIM, ERS, W3 Professur Pneumologische Rehabilitation. M. Brose, D.-M. Denk-Linnert, K. Kurz, C.A. Müller, H. Rittmannsberger, P. Sator, J. Wancata und M. Wendler geben an, dass kein Interessenkonflikt besteht.
